# The complex evolution of the metazoan HSP70 gene family

**DOI:** 10.1038/s41598-021-97192-9

**Published:** 2021-09-07

**Authors:** Er-meng Yu, Tatsuki Yoshinaga, Frank L. Jalufka, Hashimul Ehsan, David B. Mark Welch, Gen Kaneko

**Affiliations:** 1 School of Arts and Sciences, University of Houston-Victoria, Victoria, TX USA; 2 Key Laboratory of Tropical and Subtropical Fishery Resource Application & Cultivation, Key Laboratory of Aquatic Animal Immune Technology of Guangdong Province, Pearl River Fisheries Research Institute of CAFS, Guangzhou, China; 3 School of Marine Biosciences, Kitasato University, Kanagawa, Japan; 4 Josephine Bay Paul Center for Comparative Molecular Biology and Evolution, Marine Biological Laboratory, Woods Hole, MA USA

**Keywords:** Molecular evolution, Evolutionary biology

## Abstract

The metazoan 70-kDa heat shock protein (HSP70) family contains several members localized in different subcellular compartments. The cytosolic members have been classified into inducible HSP70s and constitutive heat shock cognates (HSC70s), but their distinction and evolutionary relationship remain unclear because of occasional reports of “constitutive HSP70s” and the lack of cross-phylum comparisons. Here we provide novel insights into the evolution of these important molecular chaperones. Phylogenetic analyses of 125 full-length HSP70s from a broad range of phyla revealed an ancient duplication that gave rise to two lineages from which all metazoan cytosolic HSP70s descend. One lineage (A) contains a relatively small number of genes from many invertebrate phyla, none of which have been shown to be constitutively expressed (i.e., either inducible or unknown). The other lineage (B) included both inducible and constitutive genes from diverse phyla. Species-specific duplications are present in both lineages, and Lineage B contains well-supported phylum-specific clades for Platyhelminthes, Rotifera, Nematoda, Porifera/Cnidaria, and Chordata. Some genes in Lineage B have likely independently acquired inducibility, which may explain the sporadic distribution of “HSP70” or “HSC70” in previous phylogenetic analyses. Consistent with the diversification history within each group, inducible members show lower purifying selection pressure compared to constitutive members. These results illustrate the evolutionary history of the HSP70 family, encouraging us to propose a new nomenclature: “HSP70 + subcellular localization + linage + copy number in the organism + inducible or constitutive, if known.” e.g., HSP70cA1i for cytosolic Lineage A, copy 1, inducible.

## Introduction

The 70-kDa heat shock protein (HSP70) family members play important roles in various cellular processes including heat shock response, folding of newly synthesized proteins, protein transport, and protein degradation. These apparently diverse functions are attributed to their chaperone activity^1,2^, by which they prevent the aggregation and misfolding of target proteins. Typically, HSP70 family members bind to denaturing or newly synthesized proteins by recognizing up to ten hydrophobic amino acid residues exposed to the protein surface because of the misfolding. The release of the HSP70 members, which is triggered by ATP hydrolysis, facilitates the proper folding of the target proteins^3^. The HSP70 family contains organelle-specific members localized in cytosol, endoplasmic reticulum (ER), mitochondria, and chloroplasts^4^, and these organelle-specific types not only perform chaperoning functions in the organelles but are also known to contribute to protein transport across organelle membranes.

HSP70 family members are often upregulated upon various stresses that disrupt protein folding, such as heat treatment, exposure to toxic materials, ultraviolet irradiation, and pathogen attack^5,6^. The upregulation is primarily regulated at the transcription level by transcription factors called heat shock factors (HSFs), particularly HSF1. Under unstressed conditions, HSF1 remains in a monomeric state. Various stresses lead to the formation of HSF1 trimers, which in turn bind to heat shock elements (HSEs) in the promoter region of HSP70 family member genes and promote their transcription. The HSF1-dependent transactivation system is generally conserved among eukaryotes, although species-specific differences in HSF1 function have been reported in many organisms such as the fruit fly *Drosophila melanogaster*^7,8^ and yeast *Saccharomyces cerevisiae*^9^. The expression of HSP70 family member is also regulated by many other mechanisms such as the unfolded protein response, chromatin modification, and other transcription factors depending on the type of stress and subcellular localization^10,11^. HSP70 family member genes have therefore been used as biomarkers of environmental stresses both in laboratory and field experiments^12–15^.

On the other hand, several cytosolic HSP70 family members show constitutive expression patterns. These proteins are involved in the folding of newly synthesized proteins and are traditionally called 70-kDa heat shock cognates (HSC70). However, the distinction between HSP70s and HSC70s remains unclear because of the occasional reports of “constitutive HSP70s,” especially from invertebrates^16–18^. These proteins were named “HSP70” because of their higher sequence similarity to known HSP70s than to HSC70s, but subsequent expression analyses failed to demonstrate their stress inducibility, resulting in assignment of the apparently self-contradicting protein names. Similarly, “stress-inducible HSC70s” have been reported in several animals including shrimps^19^, snails^20^, and fish^21^. This confusion is likely attributable to the poorly understood evolutionary relationship between metazoan HSP70s and HSC70s, which is due to the lack of cross-phylum comparisons. A previous phylogenetic analysis indicated that metazoan HSP70 family members can be classified into invertebrate HSP70s, vertebrate HSP70s, and HSC70s from both vertebrates and invertebrates^22^, but this study comprises only a few phyla. In-depth phylogenetic analysis of HSP70 family members with a broad sampling across phyla would provide further insight into the classification of this important group of molecular chaperones.

In this study, we sought to trace the evolutionary history of metazoan HSP70 family members, with a particular attention to their stress inducibility, using phylogenetic analyses that included Porifera, Cnidaria, Gnathifera, Lophotrochoza, Ecdysozoa, and Chordata. The specific hypothesis was that this broad sampling would provide essential information necessary to understand the evolutionary relationship between metazoan HSP70s and HSC70s. Our results add novel groups of stress-inducible HSP70, providing important insight into the evolutionary history of the metazoan HSP70 family.

## Results

### Phylogenetic analysis of HSP70 family members

We assembled a set of 125 HSP70 family members, including members associated with the mitochondria and ER, and many sequences known only from automated genome annotation as “HSP70” (Supplementary Table S1). Taxa were chosen to represent diverse phyla an included representatives of Porifera (1 species); Cnidaria (3 species from 1 class); Platyhelminthes (4 species from 3 classes); Gnathifera (6 species from two classes of rotifers); Lophotrochozoa (4 species from two classes of annelids, 1 brachiopod, and 6 species from 3 classes of molluscs); Ecdysozoa (6 species from 4 classes of arthropods, 6 species from 2 classes of nematodes, and 1 priapulid); and Chordata (9 species from 3 classes), with sequences from the yeast *Saccharomyces cerevisiae*. When possible, we chose reference or commonly recognized taxa (*e.g., Drosophila melanogaster, Caenorhabditis elegans, Nematostella vectensis*), and taxa with multiple HSP70 family members; we also included all 8 human Heat Shock Protein Family A members for reference (HSPA1A and HSPA1B are identical at the amino acid level).

**Figure 1:**
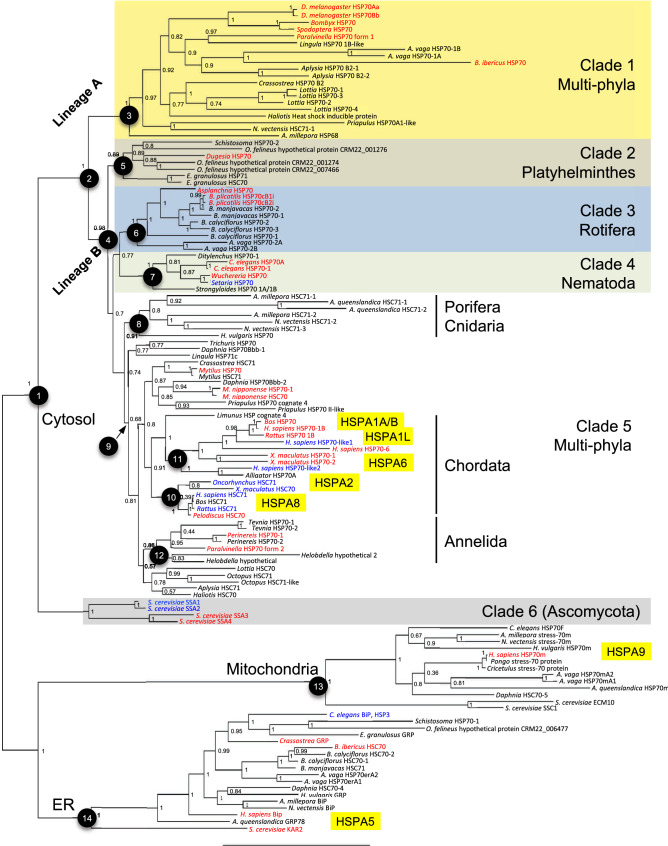
Bayesian consensus tree of HSP70 family members. Nodes discussed in the text are indicated. Numbers on the branches indicate the posterior probability support for each node. Stress-inducible and constitutive genes are shown in red and blue, respectively. No data on the stress inducibility is available for genes shown in black. Approved HGNC names of human HSP70 family members are shown in yellow boxes for reference. The DDBJ/EMBL/GenBank accession numbers and other information are summarized in Supplementary Table S1. Clades 1 to 6 include only cytosolic members of the HSP70 family. Human HSPA1A and HSPA1B have identical sequences at the amino acid level and are shown together.

To assess the robustness of the results we used two different alignment methods (T-Coffee and M-Coffee^23^; M-Coffee itself combines multiple alignment algorithms) to construct consensus trees with maximum-likelihood (RAxML) and Bayesian (MrBayes) approaches. The four trees were in general agreement for major nodes; Fig. 1 shows the Bayesian consensus tree of the M-Coffee alignment. In all analyses HSP70 family members known to be mitochondria- and ER-specific formed two monophyletic groups (nodes 13 and 14 in Fig. 1) in agreement with previous reports^4,24,25^. All family members known to be cytosolic formed a third group (node 1; clades 1–6 in Fig. 1), with yeast cytosolic HSP70s (clade 6) basal to all metazoan cytosolic HSP70s. The tree topology generally supported the species phylogeny. Within the metazoan cytosolic HSP70s (node 2; clades 1–5) there were two large clades (called lineages A and B): Lineage A was well supported and contained of genes from arthropods, annelids, brachiopods, rotifers, molluscs, priapulids, and cnidarians (node 3; clade 1); Lineage B was more poorly resolved and represented by many diverse vertebrate and invertebrate phyla (node 4; clades 2–5). There are two groups of vertebrate cytosolic HSP70s (nodes 10 and 11), which form a monophyletic group. Unexpectedly, we found three additional clusters of cytosolic HSP70 family composed of HSP70 genes from the phylum Platyhelminthes (node 5, clade 2), Rotifera (node 6; clade 3) and Nematoda (node 7; clade 4). This suggests the evolution of three unique subgroups of HSP70, distinct from conventional vertebrate and invertebrate HSP70s. Within the clade 5, Porifera and Cnidaria HSP70s were basal to other HSP70 family members, indicating that this clade has been conserved throughout metazoan evolution.

Figure 1 reveals that in many cases a species with multiple HSP70 family members has two closely related paralogs that are not informative to the deep history of the gene family. We therefore also constructed phylogenetic trees using a reduced set of 48 sequences, eliminating one of a paralog pair unless their inducibility had been previously determined (Supplementary Figs. S1 and S2). The overall topology and clustering patterns were retained, including both of the two HSP70 lineages as well as most of the nodes described above, although several clades were poorly resolved. Thus the analyses leading to the tree shown in Fig. 1 are unlikely to have been adversely effected by the large number of sequences included.

**Figure 2:**
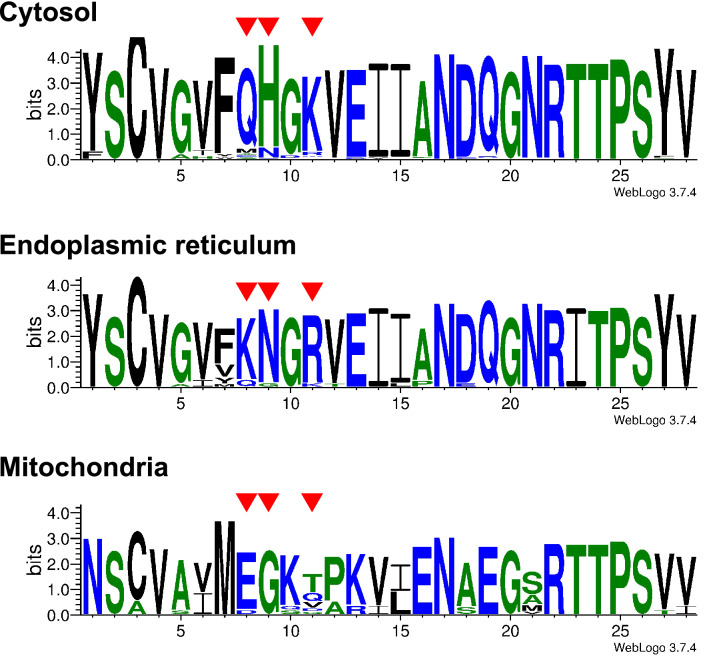
Logo visualization of cytosol-, endoplasmic reticulum (ER)-, and mitochondria-specific sequences near the N terminus of the HSP70 family members. All sequences in Fig. 1 were used to compute the sequence logos (93, 18, and 13 sequences for cytosol-, ER, and mitochondria-specific members, respectively). Compared to the cytosolic form, ER-specific forms have several conserved amino acid substitutions: Q8K, H9N, K11R, and T23I. Similarly, mitochondrial forms have Y1N, G5A, F7M, Q8E, H9G, G10K, K11T/Q/V, V12P/A, E13K/R, I14V, I15L, A16E, D17A, Q18E, and N21S/A/M. Residues that can be useful for discriminating organelle-specific members are shown with red arrows. Position 23 also discriminates ER-specific members from others.

The phylogenetic breadth of our analyses provided the opportunity to search for sequence motifs characteristic of different groups within the HSP70 family. We identified a single region near the N terminus (but after signal peptide motifs) that discriminates between metazoan mitochondrial, ER, and cytosolic HSP70s (Fig. 2), which enables better discrimination for organelle-specific HSP70s together with the well-known C-terminal motifs for cytosolic (EEVD) and ER (HDEL/KDEL) HSP70s (Supplementary Fig. S3). We also found the RARFEEL motif, previously described as possibly cytosolic-specific in conjunction with the EEVD motif^20,26,27^, in several invertebrate genes that are found with high confidence in the ER-associated node 14 (Fig. 1; *B. ibericus* HSC70, *C. gigas* GRP, and *B. manjavacas* HSC71). This result suggests that RARFEEL is not a reliable predictor of cytosol association. Indeed, this motif was identified by a comparison of a small number sequences^28^ and appears to have limited phylogenetic conservation.

**Figure 3:**
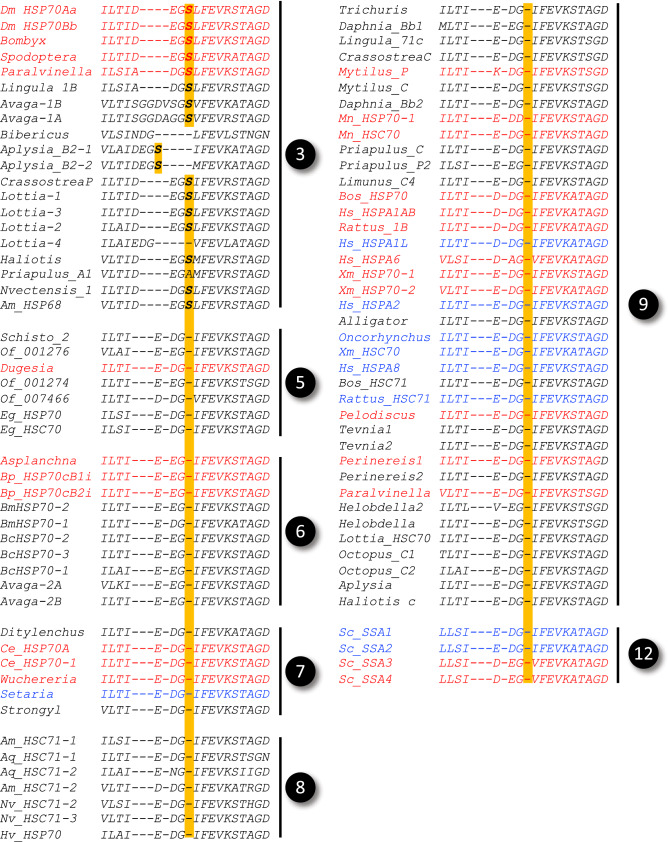
The serine residue specific to node 3. Cytosolic HSP70 family members in Fig. 1 were aligned by M-coffee, and sequences around the node 3-specific serine residue are shown in the same order as Fig. 1. Nodes in the phylogenetic tree (Fig. 1) are indicated on the right margin, and serine residues specific to node 3 are shown in bold with highlights. Stress-inducible and non-inducible genes are shown in red and blue, respectively. No data on the stress inducibility is available for genes shown in black. The accession numbers and other information are summarized in Supplementary Table S1.

Stress-inducible invertebrate HSP70s are reported to have an extra serine residue in their ATPase domain^22,29^. In our analysis this insertion occurs only in Lineage A irrespective of the stress-inducibility, where it is present in all sequences except *B. ibericus* HSP70 and one of four *Lottia* HSP70s; in *Priapulus* HSP70-1 the serine has been replaced by alanine (Fig. 3). These results suggest that the extra serine residue is a characteristic of Lineage A and may be associated with the stress-inducibility of the ancestral HSP70, which will be tested by broader sampling focusing on this residue. The alignment result supports the idea that HSP70s from Nematoda and Rotifera evolved separately from those in Annelida, Mollusca, and Arthropoda, which have been generally recognized as "invertebrate HSP70s".

### Synonymous and nonsynonymous substitution rates

Lastly, we calculated the synonymous and nonsynonymous substitution rates of 34 representative cytosolic HSP70 member genes. We primarily selected genes with known expression patterns (stress-inducible or constitutive) from each clade, hypothesizing that constitutive HSP70 family members have been under stronger purifying selection compared to stress-inducible members. Because inducibility is only known for one member of Clade 2, we did not examine this group. The rotifer sequence *B. ibericus* HSP70 (GU574486) has an insertion at nucleotide position 403 and a nucleotide deletion at position 563, causing a frame shift. Deduced amino acid sequences encoded by nucleotides 403–563 were markedly different from those of other HSP70 family members because of the frame shift, which resulted in the long branch to this sequence in phylogenetic trees (Fig. 1). Because the frame shift greatly affects the synonymous and nonsynonymous substitution rates, we excluded this sequence.

**Figure 4:**
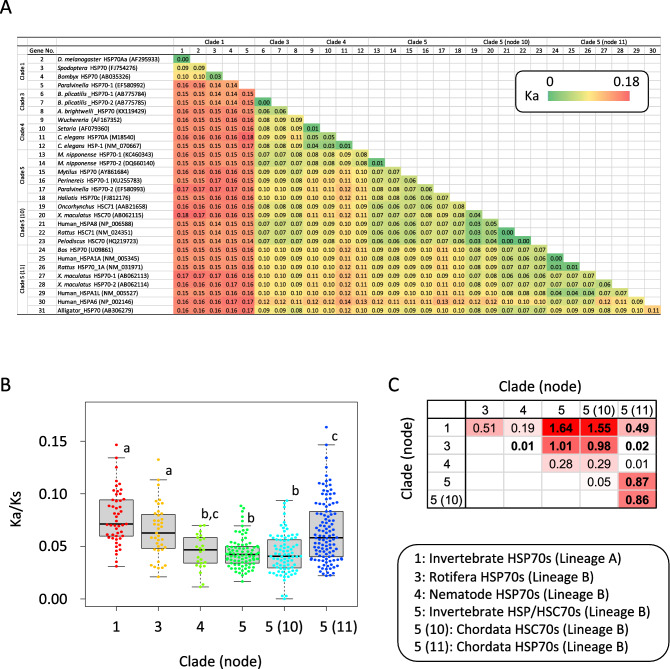
Selection pressure on cytosolic HSP70 family member genes. (**A**) Nonsynonymous substitution rates (Ka values) between 34 HSP70 family member genes. Gene number 1 is the fruit fly HSP70 Bb (AF295957) gene. The substitution rate of fruit fly HSP70 Aa and Bb gene was 00395526, which is shown as 0.00 in the figure. Accession numbers for other genes are indicated in the figure. Clades and nodes correspond to those in Fig. 2. (**B**) Bee swarm boxplots of Ka/Ks values for each clade. Only Ka/Ks values calculated from inter-cluster pairs were averaged. Statistical differences were calculated by Kruskal–Wallis test (chi-squared = 88.704, df = 5, *P* < 2.2e−16) followed by the non-parametric post-hoc tests (pairwise Wilcox test with *P* value adjustment by the Holm method). Clades sharing same letters are not significantly different at the 5% level of significance. One outlier in clade 3 (0.53) is not included in the plot (see supplementary material 1) although this value was used for all statistical analyses. (**C**) Effect sizes (Cohen’s d) for all comparisons. Combinations where significant differences were found in the pairwise Wilcox test are shown in bold.

For the selected 34 sequences representing cytosolic members of the metazoan HSP70 family, we calculated synonymous and nonsynonymous substitution rates (Ks and Ka) from the well conserved regions of the alignment (Supplementary Fig. S4) for all combinations of sequences (Fig. 4A). Synonymous substitution rates (Ks) were saturated in many combinations and only a limited number of combinations produced meaningful results (229 out of 465 combinations, Supplementary Fig. S5). The Ka/Ks values calculated for the 229 combinations were averaged for each clade (Fig. 4B). Overall, Ka/Ks values were markedly lower than 1, indicating that cytosolic HSP70 family member genes have been under purifying selection. The purifying selection pressure appears to have been less in Lineage A (clade 1) than in Lineage B (clades 3–5). The effect size between these lineages ranged from 0.19 to 1.64 (Fig. 4C). Since the effect sizes larger than 0.8 indicate large difference in multiple comparisons, these results support our hypothesis that constitutive HSP70 family members [clade 4, clade 5, and clade 5 (node 10)] have been under stronger purifying selection.

Within Lineage B, the selection pressure was strongest in Nematode HSP70s (clade 3), invertebrate HSC70/HSP70s (clade 5), and vertebrate HSC70s (clade 5, node 10). In particular, vertebrate HSC70s (clade 5, node 10) and HSP70s (clade 5, node 11) showed significant difference in the Ka/Ks with a large effect size of 0.86 despite their close phylogenetic relationship. These results also support the above hypothesis. To confirm how much this finding depends on the sampling, we conducted the same analysis with a larger sample size, including most cytosolic genes used in the phylogenetic analysis (77 genes), and obtained consistent results (Supplementary Figs. S6, S7).

## Discussion

The monophyletic origin of HSC70s was proposed by Nikolaidis and Nei (2004) based on their analysis using *Drosophila* and nematode sequences^25^. A subsequent phylogenetic analysis by Kourtidis et al. (2006) classified metazoan cytosolic HSP70s into three groups: invertebrate HSP70, vertebrate HSP70, and HSC70 including both vertebrate and invertebrate orthologues using data from multiple molluscs plus *D. melanogaster*, *C. elegans*, *Danio rerio*, and *H. sapiens*^22^. Surprisingly, to our knowledge, there has been no significant update on the evolution of metazoan HSP70 family after these small-scale studies despite the continuing expansion of available genomic data. Here we show that there are at least two types of ancestral cytosolic HSP70 family member genes in all analyzed phyla of metazoans, one (node 3; clade 1) giving birth to Lineage A of invertebrate HSP70s, and a second (node 4; clade 5) to Lineage B of both vertebrate and invertebrate HSP70s and all HSC70 genes. This second lineage has further diversified within diverse phyla: Platyhelminthes (node 5, clade 2), Rotifera (node 6; clade 3), Nematoda (node 7; clade 4), and Chordata (nodes 10 and 11; clade 5) (Fig. 1), which has not been identified in the previous reports. The tree topology is generally consistent with the species phylogeny, indicating that the effect of sequence contamination is very limited.

This newly identified evolutionary history of metazoan HSP70s demonstrates that stress inducibility has repeatedly evolved in many lineages of metazoans (i.e., convergent evolution). The multiple acquisition of stress inducibility has been observed in HSP70 family members duplicated within various taxa, such as those in a protist species and its close relative^30^ and in mollusks^22^. To better understand this convergent evolution process, it is useful to discriminate between general stress inducibility and heat inducibility. For example, human mitochondrial HSP70 (mortalin/HSPA9) and ER-associated HSPA5 (BiP/GRP78) are not induced by heat but are induced by radiation^31^ and the unfolded protein response^32^, respectively. Comprehensive promoter analyses will add further mechanistic insights into the process by which HSP70 family members have acquired inducibility.

Another implication of our phylogenetic analysis is the nature of the ancestry HSP70 family members. Inter-clade comparison of the synonymous and nonsynonymous substitution rates shows that while all HSP70 family members are under strong purifying selection, the pressure to conserve amino acid sequence varies across clades. The presence of purifying selection has been reported for mammalian^33^, nematode^25^, molluscan^22^ and protist^30^ HSP70 genes, and our multi-phylum analysis was consistent with these reports. Most dramatically, the canonical HSC70 genes of vertebrates (node 10; clade 5) showed stronger purifying selection pressure than the HSP70 family members in node 11 (clade 5). This concept can be further generalized by thoroughly investigating the saturation rate between each HSP70 gene^34^. These results are in line with the concept that the role of HSC70 proteins, the folding of newly synthesized proteins, is more essential for cell survival compared to the role of the HSP70 cognates (node 11; clade 5). Indeed, cells deficient in the HSC70 gene are non-viable^35^, whereas HSP70 knockout mice are mostly viable and fertile, although they are known to be sensitive to stress^1^. The function of constitutive human HSPA8 is more diverse than other HSP70 family members and relates to many essential processes such as endocytosis and autophagy^36^. From their relative importance it is possible to envision that constitutive expression may be ancestral in the metazoan cytosolic HSP70 family, but because of the presence of inducible and constitutive genes in many clades of metazoan cytosolic HSP70, it is difficult to predict the ancestral state with certainty. In addition, branch lengths between clades are not uniform, suggesting different evolutionary rates and gene conversion may occur between the paralogs found in many species. In this regard our data should be interpreted in a semiquantitative manner.

It is known that HSP70 paralogs in nematode, mammalian, and molluscan have experienced gene conversion events^22,25,33^. In addition to purifying selection, gene conversion also likely contributes to the highly conserved nature of the HSP70 family members found in this study. The tree topology might have been affected by the gene conversion in closely related paralogues and orthologues such as human HSPA1A and HSPA1B^33^. However, gene conversion generally functions to conserve the sequence in a species, and thus the overall topology of the metazoan tree is unlikely to be significantly affected. Gene conversion rate would need to be about 10 times higher than the mutation rate to significantly affect the topological distance according to a genomic simulation study^37^, but the nucleotide identity between sequences is often lower than 70% in our phylogenetic analysis.

**Figure 5:**
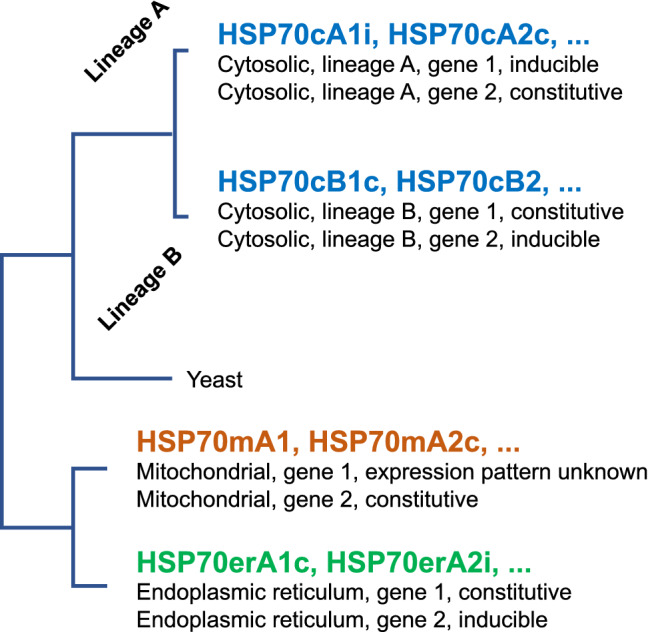
A proposed new nomenclature for HSP70 family members.

Based on the largest-ever cross-phylum phylogenetic analysis of the HSP70 family, we propose a new nomenclature that reflects the phylogenetic relationship of family members rather than assumptions inferred about “cognate” or “constitutive” status (Fig. 5). The proposed name of an HSP70 family member protein consists of “HSP70 + subcellular localization + linage + copy number found in the organism + inducible or constitutive, if known.” For example, HSP70cA1i represents a cytosolic HSP70 in Lineage A, copy 1, inducible; and HSP70mA1 for mitochondrial linage of unknown inducibility. Although the current study found only one lineage in mitochondrial and ER members, the use of “A” in these organelle-specific isoforms would be beneficial. Systematic lineage names such as HSP70cD and HSP70mB can be assigned for novel lineages that may be discovered in future. Gene names are represented by lowercase italics of protein names (e.g., *hsp70cb2i* and *hsp70era1*).

In conclusion, the present study illustrated the evolutionary history of the metazoan HSP70 family, in which stress inducibility does not reflect evolutionary history. We also report the presence of novel signature sequences near the N terminus associated with cellular localization, and a previously unreported conserved serine residue. These findings, with the proposed nomenclature based on molecular evolution, will facilitate classification of new HSP70 family members and will help us understand the evolution and function of the HSP70 family.

## Methods

### Phylogenetic analysis

Metazoan HSP70 family genes were identified by BLAST searches to the NCBI nr database and to annotated and unannotated genome and transcriptome databases. Sequences were chosen to maximize phylogenetic diversity across Metazoa and where possible within phyla, and prioritized sequences where inducibility had been experimentally determined. Reference taxa and widely recognized taxa, and taxa with multiple annotated HSP70 family members were also prioritized. In some cases where there were multiple nearly-identical sequences in a species one representative sequence was chosen, although all human sequences were included for reference.

Alignments were generated with T-Coffee and M-Coffee using the tcoffee server at http://tcoffee.crg.cat with default parameters. The alignment revealed several sequences with extensive amino-terminal or carboxy-terminal extensions beyond the length of well characterized genes from model species. Because these are likely misannotated we excluded these regions from analysis and used the region of the alignment corresponding to human HSPA1A/HSPA1B positions 6-616.

Bayesian analyses were performed using MrBayes (v3.2.6) with two simultaneous runs of 4 chains each for 4 × 10^6^ generations sampling every 100 generations. Evolutionary models were sampled during the run and for all runs with all alignments the WAG model was chosen with a posterior probability of 1. In all cases mcmc diagnostics found the average standard deviation of split frequencies between the runs to be < 0.05, and ESS and PSRF convergence diagnostics indicated trees were sufficiently sampled. Consensus trees were generated by sampling the last 2 × 10^5^ trees. Maximum likelihood analyses were performed using raxml-ng (v0.9.0) using the WAG model and combined ML tree search with 250 bootstrap trees, and default parameters. Trees were visualized with FigTree (v1.4.2).

### Sequence analysis

Sequence logos were computed for the M-coffee alignments of cytosol-, ER, and mitochondria-specific members in Fig. 1 using WebLogo 3.7.4 (http://weblogo.threeplusone.com).

### Synonymous and nonsynonymous substitution rates

R version 4.0.4 was used for the calculation of the synonymous and nonsynonymous substitution rates (the kaks function), Kruskal–Wallis test (kruskal.test function), and post-hoc test (pairwise.wilcox.test function) on the Macintosh platform. Heat maps were created in Microsoft Excel 2016 (Redmond, MA).

## Supplementary Information

Supplementary Information.
