# Supplementary material for: The complex evolution of the metazoan HSP70 gene family
Source: Sci Rep. 2021 Sep 7;11:17794. doi: 10.1038/s41598-021-97192-9 (PMC8423806; doi:10.1038/s41598-021-97192-9)
Supplement: Supplementary file 1 — Supplementary Information. [file 41598_2021_97192_MOESM1_ESM.docx]

**Supplementary Fig. S1.** Maximum likelihood tree of the reduced alignment of HSP70 family members. A bootstrap consensus tree was constructed by the maximum likelihood method using the LG + G model. The bootstrap values from a 1000-replicate analysis are given at the nodes in percentage. Clades in Fig. 1 are indicated in the tree. Stress-inducible and constitutive genes are shown in red and blue, respectively. No data on the stress inducibility is available for genes shown in black.

**Supplementary Fig. S2.** Bayesian consensus tree of the reduced alignment of HSP70 family members. Clades in Fig. 1 are indicated in the tree. Numbers on the branches indicate the posterior probability. Stress-inducible and constitutive genes are shown in red and blue, respectively. No data is available on the stress inducibility for genes shown in black.

Aplysia_HSP70-2 ----------------------------------------------MTKGMKTPAIGIDL 14

Haliotis_HSP70 ----------------------------------------------MAPKGKSPAIGIDL 14

Lottia_HSP70-4 ------------------------------------------------MAPKGPAIGIDL 12

Bplicatilis_HSP70-1 -------------------------------------------------MSKGPAVGIDL 11

Avaga_HSP70erA -----------------------MKIFTALSLIALTTIFTFSSASEDAKKDIGTVIGIDL 37

Bibericus_HSC70 -----------------------MNKLGLFLLLVLSISSYLNAQKDEKKESYGTVIGIDL 37

BcHSC70-2 ----------------------------------------------------GTVIGIDL 8

Celegans_HSP70F MLSARS-------------------FLSSARTIA---RSSLMSARSLSDKPKGHVIGIDL 38

Daphnia_HSP70c5-1 ------------------------------------------------------------ 0

Pongo_HSP70m MISASRAVAARLVGAAASRGPTAARYQDGWNGLSHEAFRIVSRRDYASEAIKGAVVGIDL 60

Hsapiens_HSP70m MISASRAAAARLVGAAASRGPTAARHQDSWNGLSHEAFRLVSRRDYASEAIKGAVVGIDL 60

Aplysia_HSP70-2 GTTYSCVGIF**QH**G**K**VEIIANDQGNR**T**TPSYVAFT-DTERLVGDAAKNQAALNPSNTIFDA 73

Haliotis_HSP70 GTTYSCVGVF**Q**NGQVEIIANDQGNR**T**TPSYVAFT-DTERLIGDAAKNQVALNPQNTVFDA 73

Lottia_HSP70-4 GTTFSCVGVF**QH**GAVEIIANDQGNR**T**TPSYVAFT-DSERLIGDSAKNQVALNPSNTIFDA 71

Bplicatilis_HSP70-1 GTTYSCVGVF**QH**G**K**VEIIANDQGNR**T**TPSYVAFT-DTERLIGDAAKNQVAMNPNNTVFDA 70

Avaga_HSP70erA GTTYSCVGIF**KN**G**R**VEIIANDQGNR**I**TPSYVAFTAEGERLIGDAAKNQLTSNPENTVFDA 97

Bibericus_HSC70 GTTYSCVGVV**KN**G**R**VEIIANDQGNR**I**TPSYVAFTSDGERLIGDAAKNQLTSNPENTIFDA 97

BcHSC70-2 GTTYSCVGVV**KN**G**R**VEIIANDQGNR**I**TPSYVAFTSEGERLIGDAAKNQLTSNPENTIFDA 68

Celegans_HSP70F GTT**N**SCVSI**MEG**K**TPKV**I**E**NAEGVR**T**TPSTVAFTADGERLVGAPAKRQAVTN**SANTLFAT** 98

Daphnia_HSP70c5-1 ---------**MEG**Q**TPKV**I**E**NAEGSR**T**TPSVIAFTKDGERLAGMPAKRQAVTN**AQNTLYAT** 51

Pongo_HSP70m GTT**N**SCV**A**V**MEG**K**QAKV**L**E**NAEGAR**T**TPSVVAFTADGERLVGMPAKRQAVTN**PNNTFYAT** 120

Hsapiens_HSP70m GTT**N**SCV**A**V**MEG**K**QAKV**L**E**NAEGAR**T**TPSVVAFTADGERLVGMPAKRQAVTN**PNNTFYAT** 120

.: ::: * :* * *** :*** : *** * **.* . * **.: :

Aplysia_HSP70-2 KRLIGRKFTDKSVQSDMKHWPFQVAE-VDSRPKIEAEYKGERKLFAPEEVSSMVLSKMKE 132

Haliotis_HSP70 KRLIGRQFDDSAVQKDMKHWPFRVVNSSGSKPKLQVEYKGEKKTFAPEEISSMVLNKMKE 133

Lottia_HSP70-4 KRLIGRKFDDATIQSDMKHWPFKVVK-DGDKPKLQAEFKNELKTFSPEEVSAMVLTKMKE 130

Bplicatilis_HSP70-1 KRLIGRKFDDLTVQADMKHWPFTVIS-DSGRPKIQVEFKGETKSFYPEEVSSMVLTKMKE 129

Avaga_HSP70erA KRLIGREFSDSSVQQDMKHFPFKVVE-KNSKPAIQISTGKEQKLFTPEEISAMVLGKMRE 156

Bibericus_HSC70 KRLIGREFKDSSVQGDMKYWPFKVVD-KNNKPHIKVKSADEEKLFAAEEVSAMVLGKMKE 156

BcHSC70-2 KRLIGREFSDPSVQGDMKYWPFKVVE-KNKKPYIKVKSGDQDKLFAPEEISAMVLGKMKE 127

Celegans_HSP70F **KR**LIGRRYEDPEVQKDLKVVPYKIVKASNGDAWVE----AQGKVYSPSQVGAFVLMKMKE 154

Daphnia_HSP70c5-1 **KR**LIGRKFEDPEVKKDMKTVSYKIVRASNGDAWVE----AQGKLYSPSQIGAFVLTKMKE 107

Pongo_HSP70m **KR**LIGRRYDDPEVQKDIKNVPFKIVRASNGDAWVE----AHGKLYSPSQIGAFVLMKMKE 176

Hsapiens_HSP70m **KR**LIGRRYDDPEVQKDIKNVPFKIVRASNGDAWVE----AHGKLYSPSQIGAFVLMKMKE 176

******.: * :: *:* : : . :: . * : .::.::** **:*

Aplysia_HSP70-2 TAEAYLGQKVTEAVITVPAYFNDSQRQATKDAGAIAGLNVLRMINEPTAAALAYGLDKGQ 192

Haliotis_HSP70 TAEAYLGQQVTDAVITVPAYFNDSQRQATKDAGAIAGLNVLRIINEPTAAALAYGLDKNL 193

Lottia_HSP70-4 TAEAYLGENVTNAVITVPAYFTDSQRSATKDAGVIAGLNVLRIINEPTAAALAYGLDKKT 190

Bplicatilis_HSP70-1 TAEAYLGKKVTDAVVTVPAYFNDSQRQATKDAGAIAGLNVLRIINEPTAAAIAYGLDKKG 189

Avaga_HSP70erA IAEAYLGKKVTHAVVTVPAYFNDAQRQATKDAGTISGLNVLRIINEPTAAAIAYGLDKK- 215

Bibericus_HSC70 IAEAYLGKPVTHAVVTVPAYFNDAQRQATKDAGTIAGMTVMRIINEPTAAVYCLRLGQK- 215

BcHSC70-2 IAEAYLGKNVTHAVVTVPAYFNDAQRQATKDAGTISGMTVMRIINEPTAAAIAYGLDKK- 186

Celegans_HSP70F TAESYLGTTVNNAVVTVPAYFNDSQRQATKDAGQISGLNVLRVINEPTAAALAYGLDKD- 213

Daphnia_HSP70c5-1 TAEAYLGTPVKNAVVTVPAYFNDSQRQATKDAGQISGLNVLRVINEPTAAALAYGMDKS- 166

Pongo_HSP70m TAENYLGHTAKNAVITVPAYFNDSQRQATKDAGQISGLNVLRVINEPTAAALAYGLDKS- 235

Hsapiens_HSP70m TAENYLGHTAKNAVITVPAYFNDSQRQATKDAGQISGLNVLRVINEPTAAALAYGLDKS- 235

** *** ...**:******.*:**.****** *:*:.*:*:*******. . :.:

Aplysia_HSP70-2 SGEKNVLIFDLGGGTFDVSVLTIDEGSMFEVKATAGDTHLGGEDFDNRLVSHFLQEFKRK 252

Haliotis_HSP70 KGEKNVLIFDLGGGTFDVPVLTIDEGSMFEVRSTAGDTHLGGEDFDNRLVEHFLQEFQRK 253

Lottia_HSP70-4 DTEQHILIFDLGGGTFDVSILAIEDG-VFEVLATAGDTHLGGEDFDNRMVNYFTQEFKRK 249

Bplicatilis_HSP70-1 GGEKNILIFDLGGGTFDVSILTIEEG-IFEVKSTAGDTHLGGEDFDNRLVNHFVEEFKRK 248

Avaga_HSP70erA EGEKNILVFDLGGGTFDVSLLTIDNG-VFEVVATNGDTHLGGEDFDQRVMEHFIKLFKKK 274

Bibericus_HSC70 EGEKNILVFDLGGGTFDVSLLTIDNG-VFEVVATNGDTHLGGEDFDNRVMEHFIKLFKKK 274

BcHSC70-2 EGEKNILVFDLGGGTFDVSLLTIDNG-VFEVVATNGDTHLGGEDFDNRVMEHFIKLFKKK 245

Celegans_HSP70F AGDKIIAVYDLGGGTFDVSILEIQKG-VFEVKSTNGDTFLGGEDFDHALVHHLVGEFKKE 272

Daphnia_HSP70c5-1 -DDKIIAVYDLGGGTFDISILEIQKG-VFEVKSTNGDTFLGGEDFDNALVNFLVNEFKRD 224

Pongo_HSP70m -EDKVIAVYDLGGGTFDISILEIQKG-VFEVKSTNGDTFLGGEDFDQALLRHIVKEFKRE 293

Hsapiens_HSP70m -EDKVIAVYDLGGGTFDISILEIQKG-VFEVKSTNGDTFLGGEDFDQALLRHIVKEFKRE 293

:: : ::********: :* *:.* :*** :* ***.*******: :: .: *::.

Aplysia_HSP70-2 HNKDISKNARATRRLRTACERAKRTLSSSSEASIEIDSLFE----GMDYYTKISRARFEE 308

Haliotis_HSP70 TRKDISNNTRAMRRLHTACERAKRTLSSSTEASIEIDSLYE----GVDFYSKISRARFEE 309

Lottia_HSP70-4 FGKDLSKSSRALRRLRTACERAKRTLSSSAEANIEIDALFE----GFDFYSKITRARFED 305

Bplicatilis_HSP70-1 NKKDITSNKRALRRLRTACERAKRTLSSSAQANIEIDSLHE----GVDFYTSITRARFEE 304

Avaga_HSP70erA TGKDVRKDHRAVQKLRREVEKAKRTLSSQHQTKIEIESFFD----NEDFSETLTRAKFEE 330

Bibericus_HSC70 TGKDIRKDNRAVQKLRREVEKAKRTLSTQFDTRIEIESFFD----GEDFSETFTRARFEE 330

BcHSC70-2 TGKDIRKDNRAVQKLRREVEKAKRTLSTQFDTRIEIESFFD----SEDFSETFTRASFEK 301

Celegans_HSP70F QGVDLTKDPQAMQRLREAAEKAKCELSSTTQTDINLPYITMDQSGPKHLNLKLTRAKFEQ 332

Daphnia_HSP70c5-1 QGLDITKDPMAMQRVKEAAEKAKIELSSSMQTDINLPYLTMDSSGPKHMNLKMSRSKLES 284

Pongo_HSP70m TGVDLTKDNMALQRVREAAEKAKCELSSSVQTDINLPYLTMDSSGPKHLNMKLSRAQFEG 353

Hsapiens_HSP70m TGVDLTKDNMALQRVREAAEKAKCELSSSVQTDINLPYLTMDSSGPKHLNMKLTRAQFEG 353

*: .. * :::: *:** **: :: *:: : . .::*: :*

Aplysia_HSP70-2 LCADLFRSTLEPVESALRDAKLDKGKIDEVVLVGGSTRIPKIQKLLSDFFNGKELNKSIN 368

Haliotis_HSP70 LCSDLFRSTLEPVEKALRDAKLDKSCIHDVVLVGGSTRIPKIQKLLQNFMNGKDLNKSIN 369

Lottia_HSP70-4 LCADLFRNTMVPVEKALKDAKLDKSKIDEVVLVGGSTRIPKIQKLLKDFMNGKELNKSIN 365

Bplicatilis_HSP70-1 LCADLFRGTLDPVEKALRDAKMDKSSVNEIVLVGGSTRIPKVQKLLQDFFNGKELNKSIN 364

Avaga_HSP70erA LNMDLFRSTMKPVQKVLEDADMKKSDIAEVVLVGGSTRIPKVQQLVKDFFDGKEPSRGIN 390

Bibericus_HSC70 LNMDLFRNTLKPVQKVLEDAGLKKTEIDEIVLVGGSTRIPKIQQLVKEYFDGKEPSRGIN 390

BcHSC70-2 LNIELFRNTLKPVQKVLEDAGLKKTDIDEIVLVGGSTRIPKVQELVKEFFNGKEPSRGIN 361

Celegans_HSP70F IVGDLIKRTIEPCRKALHDAEVKSSQIADVLLVGGMSRMPKVQATVQEIF-GKVPSKAVN 391

Daphnia_HSP70c5-1 LVGDLIKRTVGPCQKALKDAEVSKNDIGDVLLVGGMTRMPKVQETVKDIF-GRVPSKAVN 343

Pongo_HSP70m IVTDLIRRTIAPCQKAMQDAEVSKSDIGEVILVGGMTRMPKVQQTVQDLF-GRAPSKAVN 412

Hsapiens_HSP70m IVTDLIRRTIAPCQKAMQDAEVSKSDIGEVILVGGMTRMPKVQQTVQDLF-GRAPSKAVN 412

: :*:: *: * ...:.** :.. : :::**** :*:**:* :.: : *: .:.:*

Aplysia_HSP70-2 PDEAVAYGAAVQAAVLTGDKSEAIRDVLLVDVAPLSLGIETAGGVMTSLIKRGSTIPAKT 428

Haliotis_HSP70 PDEAVAYGAAVQAAVLSGDSSDAIKDVLLVDVAPLSLGIETAGGVMTKLIDRNTRIPTKA 429

Lottia_HSP70-4 PDEAVAYGAAVQAAVLIGDKSEKIKDVLLVDVAPLSLGIETAGGVMTRIIERNTRIPTKH 425

Bplicatilis_HSP70-1 PDEAVAYGAAVQAAILTGDKSEAVQDLLLLDVAPLSLGIETAGGVMTALIKRNTTIPTKQ 424

Avaga_HSP70erA PDEAVAYGAAVQAGVLSGEEN--TGDIVLLDVNPLTMGIETVGGVMTKIIPRNTVIPTKK 448

Bibericus_HSC70 PDEAVAYGAAVQGGVLSGEDV--GGEVVLLDVNPLTMGIETVGGVMTKLIPRNTVIPTKN 448

BcHSC70-2 PDEAVAYGAAVQGGVLSGEDV--GGDVVLLDVNPLTMGIETVGGMMTKLISRNTVIPTKK 419

Celegans_HSP70F PDEAVAMGAAIQGAVLAGD----VTDVLLLDVTPLSLGIETLGGIMTKLITRNTTIPTKK 447

Daphnia_HSP70c5-1 PDEAVAVGAAIQGGVLSGG----VTDILLLDVTPLSLGIETLGGVFTKLIQRNTTIPTKK 399

Pongo_HSP70m PDEAVAIGAAIQGGVLAGD----VTDVLLLDVTPLSLGIETLGGVFTKLINRNTTIPTKK 468

Hsapiens_HSP70m PDEAVAIGAAIQGGVLAGD----VTDVLLLDVTPLSLGIETLGGVFTKLINRNTTIPTKK 468

****** ***:*..:* * :::*:** **::**** **::* :* *.: **:*

Aplysia_HSP70-2 SQVFTTYSDNQPGVDIQVFEGERAMTKDNNLLGKFQLSGIPPAPRGVPQIEVTFDVDANG 488

Haliotis_HSP70 SQIFTTYSDNQPGVSIQVFEGERALTKDNNILGKFELTGIPPAPRGVPQIEVTFNIDANG 489

Lottia_HSP70-4 SQIFTTYSDNQPGVSIQVYEGERTMTKDNHPMGNFELSGIPPAPRGVPQIEVSFDIDANG 485

Bplicatilis_HSP70-1 TQTFTTYADNQPGVLIQVYEGERAMTKDNHLLGKFELSGIPPAPRGVPQIEVTFDIDANG 484

Avaga_HSP70erA SQVFSTAAENQPSVTIQVFEGERPMTKDNHVLGKFDLTGIPPAPRGVPQIEVTFEIDVNG 508

Bibericus_HSC70 -PKFSTASDNQPTVTIAVYEGERPMTKDNHLLGKFDLTGIPPAPRGVPQIEVTFEIDANG 507

BcHSC70-2 SQVFSTAADNQPSVTIAVYEGERPMTKDNHLLGKFDLTGIPPAPRGVPQIEVTFEIDVNG 479

Celegans_HSP70F SQVFSTAADGQTQVQIKVFQGEREMATSNKLLGQFSLVGIPPAPRGVPQVEVTFDIDANG 507

Daphnia_HSP70c5-1 SQVFSTAADGQTQVEIKVYQGEREMAADNKILGQFSLIGIPPAPRGVPQIEVTFDIDANG 459

Pongo_HSP70m SQVFSTAADGQTQVEIKVCQGEREMAGDNKLLGQFTLIGIPPAPRGVPQIEVTFDIDANG 528

Hsapiens_HSP70m SQVFSTAADGQTQVEIKVCQGEREMAGDNKLLGQFTLIGIPPAPRGVPQIEVTFDIDANG 528

*:* ::.* * * * :*** :: .*: :*:* * ***********:**:*::*.**

Aplysia_HSP70-2 ILNVTAQDKSTGKSSHITIKNERGRLSQAEIDRMLADADKYKDEDEKQRERVSARNQLET 548

Haliotis_HSP70 ILNVSAVDKSTGKSNNVTISDDQSRLSKADIDRMVSEAERYKEEDEKHKERIAARNHLEN 549

Lottia_HSP70-4 ILNVLAEDKSTGKSNKITITNDKGRLSSEEIEKLVQEAEKYKDEDEKQKRKISARNQLES 545

Bplicatilis_HSP70-1 ILNVSAADKSTGKTNKITITNDKGRLSKEEIDRMVNEAEKYKKDDEEQRDKVAAKNSLES 544

Avaga_HSP70erA ILKVTAEDKGTGNKNNIVINSNTNRLSPDEIDRMIKDSEKFADEDKKVKERVDAKNELES 568

Bibericus_HSC70 ILKVSAEDKGTGNKNNIVINNNQNRLSPEEIERMIKDAEKFADEDKKVKEKVEAKNELES 567

BcHSC70-2 ILKVTAEDKGTGNKNNIVINNNQNRLSPEEIEKMIKDAEKFADEDKKVKEKVEAKNELES 539

Celegans_HSP70F IVNVSARDRGTGKEQQIVIQS-SGGLSKDQIENMIKEAEKNAAEDAKRKELVEVINQAEG 566

Daphnia_HSP70c5-1 IVHVSARDKGTGKEQQIVIQS-SGGLSKDEIENMVRNAEQFAKEDQVKRDRVEAVNHAEG 518

Pongo_HSP70m IVHVSAKDKGTGREQQIVIQS-SGGLSKDDIENMVKNAEKYAEEDRRKKERVEAVNMAEG 587

Hsapiens_HSP70m IVHVSAKDKGTGREQQIVIQS-SGGLSKDDIENMVKNAEKYAEEDRRKKERVEAVNMAEG 587

*::* * *:.**. .::.* . . ** :*:.:: :::: :* : : . * *

Aplysia_HSP70-2 YIFHVKQAV-DA--AGDKLPSPDKDAVLKACNDSLSWLDNNSLADKEEVEDRLKELQKTT 605

Haliotis_HSP70 YVFSVRASV-DE--MGSKQEDVDKQTVSKACEETLRWLDNNSLAEKEEFEQQYKQLEKLC 606

Lottia_HSP70-4 YVFNVKQVL-DNG-GADKICDSDKETVKQCCDETIQWLDNNALAEKDEYEHRLDEVQKVC 603

Bplicatilis_HSP70-1 YCFNMKQTV-EDEKLAAKISADDKKKILDACEEALKWLDSNQTAEKDEFEHKMKEVEKIC 603

Avaga_HSP70erA YAYSLKTQLNDKEKLGGKLSSDDKETIEKAVEEQIKWLESNQGAEVDELKEHKKQLEEIV 628

Bibericus_HSC70 YVYSLKNQLNDKEKLGNKLSEDEKETINSAVDEKIKWLESNSDAEVDDFKEQKKALEEIV 627

BcHSC70-2 YVYSLKNQLSDKEKLGGKLSEEDKEKIQSSVEEKIKWLESNSNAEVEEFKDQKKELEEIV 599

Celegans_HSP70F IIHDTEAKMT---EFADQLPKDECEALRTKIADTKKILDNKDNETPEAIKEACNTLQQQS 623

Daphnia_HSP70c5-1 IVHDTESKME---EFKEQLPAEECSKLREQISAVRDMLVNKDSKTPEEIKKTTNDLQQAS 575

Pongo_HSP70m IIHDTETKME---EFKDQLPADECNKLKEEISKMRELLARKDSETGENIRQAASSLQQAS 644

Hsapiens_HSP70m IIHDTETKME---EFKDQLPADECNKLKEEISKMRELLARKDSETGENIRQAASSLQQAS 644

. . : : : . : * : : .. . :::

Aplysia_HSP70-2 SSVMAKLHTQGQSQGQGSS---------------QGSSGPHGPSV**EEMD** 639

Haliotis_HSP70 SPVMSKLHNKGCGQQ-------------------QQGGSSKGPTV**EEMD** 636

Lottia_HSP70-4 SPIMTKLHGEANGQSTD-PTG--------------RSGGNQGPTV**EEID** 637

Bplicatilis_HSP70-1 SPIITQLYQGAGGAPGGMPGGMPGGMPGGMPGAGPESAGRSGPTI**DEVD** 652

Avaga_HSP70erA TPIMTKLYGQGGAGAGAGPEDVPPPPPHGHD**DDSL**-------------- 663

Bibericus_HSC70 NPIMTKLYQQNSENAGSSDSQT**NDEL**----------------------- 653

BcHSC70-2 NPIMSKLY**QQSG**------------------------------------- 611

Celegans_HSP70F LKLFEAAYKNMAAKNSGGDAQEAKT---AEE-----------PKKEQN- 657

Daphnia_HSP70c5-1 LKLFEMAYKKMAADRESSGGSTNSGDSTGST-----------DKKEDKQ 613

Pongo_HSP70m LKLFEMAYKKMASEREGSGSSGTGEQ---KE-----------DQKEEKQ 679

Hsapiens_HSP70m LKLFEMAYKKMASEREGSGSSGTGEQ---KE-----------DQKEEKQ 679

:: :

**Supplementary Fig. S3.** Alignment of several HSP70 family members with variations in the known cytosolic (EEVD), endoplasmic reticulum (ER; HDEL/KDEL), and mitochondrial (PEAEYEEAKK) motifs. The three motifs are shown in bold. Distinctive amino acid residues in the newly identified motifs are shown in red (cytosolic), blue (ER), and green (mitochondrial). The discrimination of organelle-specific HSP70 family members becomes more evident by using multiple motifs.

Drosophila_Bb_AF295957 ---------------ATGCCTGCTATTGGAATCGATCTGGGCACCACCTACTCCTGCGTG 45

Drosophila_Aa_AF295933 ---------------ATGCCTGCTATTGGAATCGATCTGGGCACCACCTACTCCTGCGTG 45

Spodoptera_FJ754276 ---------------ATGCCAGCCATTGGAATAGATCTGGGTACCACATACTCGTGCGTC 45

Bombyx_AB035326 ---------------ATGCCAGCTATCGGAATCGATTTGGGAACGACATACTCATGTGTT 45

Paralvinella_EF580992 ATGGCACAAGGAAAAACACCGGCAATTGGCATTGATCTTGGCACCACCTATTCCTGTGTT 60

B_plicatilis_HSP70-1_AB775784 ------ATGAGCAAAGGACCCGCTGTAGGCATCGACTTGGGCACCACGTACTCTTGCGTG 54

B_plicatilis_HSP70-2_AB775785 ------ATGAGCAAAGGACCCGCTGTAGGCATCGACTTGGGCACCACGTACTCTTGCGTG 54

A_brightwelli_HSP70_KX119429 ---------ATGGCCAAAACAGCGATCGGTATTGATTTGGGCACGACATATTCGTGTGTG 51

Wuchereria_AF167352 ---------ATGTCAAAGAATGCCATTGGTATCGATCTTGGAACCACGTATTCCTGCGTG 51

Setaria_AF079360 ---------ATGTCAAAGAACGCAATCGGCATTGATCTTGGGACTACTTACTCGTGCGTG 51

C_elegans_HSP70A_M18540 ------ATGAGTAAGCATAACGCTGTTGGAATCGATTTGGGAACTACCTACTCCTGCGTG 54

C_elegans_HSP-1_NM_070667 ------ATGAGTAAGCATAACGCTGTTGGAATCGATTTGGGAACTACCTACTCCTGCGTG 54

M_nipponense_KC460343 ------ATGGCTAAAAGTGCAGCTGTCGGGATTGACCTGGGTACCACCTACTCATGTGTA 54

M_nipponense_DQ660140 ------ATGGCTAAAAGTGCAGCTGTCGGGATTGACCTGGGTACCACCTACTCATGTGTA 54

Mytilus_HSP70_AY861684 ---ATGGCAAAAACAGGACCAGCAATTGGAATAGATCTTGGAACTACATACTCCTGTGTT 57

Perinereis_KU255783 ---------ATGGCAGCACCTGCAGTAGGTATTGATCTCGGCACCACATACTCCTGCGTT 51

Paralvinella_HSP70-2_EF580993 ------ATGGCGAAGGCAAGTGCTGTGGGTATCGACCTTGGAACCACGTACTCTTGCGTT 54

Haliotis_FJ812176 ------ATGGCCAAGGCACCAGCTATTGGTATAGATCTGGGGACCACCTACTCGTGTGTA 54

Oncorhynchus_HSC71_AAB21658 ------ATGTCTAAGGGACCAGCAGTCGGCATCGATCTCGGGACCACCTACTCCTGCGTG 54

X_maculatus_HSC70_AB062115 ------ATGTCCAAGGGACCAGCTGTAGGCATTGACCTGGGCACTACCTACTCTTGTGTG 54

Human_HSPA8_HSC71_NP_006588 ------ATGTCCAAGGGACCTGCAGTTGGTATTGATCTTGGCACCACCTACTCTTGTGTG 54

Rattus_HSC71_NM_024351 ------ATGTCTAAGGGACCTGCAGTTGGCATTGATCTTGGCACCACCTACTCCTGTGTG 54

Pelodiscus_HSC70_HQ219723 ------ATGTCTAAGGGACCAGCAGTTGGAATTGATCTTGGCACCACATACTCCTGTGTT 54

Bos_HSP70_U09861 ------ATGGCGAAAAACATGGCTATCGGCATCGACCTGGGCACCACCTACTCCTGCGTA 54

Human_HSPA1A_NM_005345 ------ATGGCCAAAGCCGCGGCGATCGGCATCGACCTGGGCACCACCTACTCCTGCGTG 54

Rattus_HSP70_1A_NM_031971 ------ATGGCCAAGAAAACAGCGATCGGCATCGACCTGGGCACCACCTACTCGTGCGTG 54

X_maculatus_HSP70-1_AB062113 ATGTCATCAGCTAAGGGAATATCCATTGGCATCGATCTGGGCACCACCTACTCTTGTGTC 60

X_maculatus_HSP70-2_AB062114 ATGTGTGCAGCTAAAAACGTGGCCATCGGCATCGACCTGGGCACCACCTACTCCTGCGTG 60

Human_HSPA1L_NM_005527 ATGGCTACTGCCAAGGGAATCGCCATAGGCATCGACCTGGGCACCACCTACTCCTGTGTG 60

Human_HSPA6_NP_002146 ATGCAGGCCCCACGGGAGCTCGCGGTGGGCATCGACCTGGGCACCACCTACTCGTGCGTG 60

Alligator_HSP70_AB306279 ---ATGTCGGGCAAAGGGCCTGCGATCGGCATCGACTTGGGCACCACGTACTCCTGCGTG 57

* * ** ** ** * ** ** ** ** ** ** **

**Drosophila_Bb_AF295957 GGTGTCTACCAGCATGGCAAGGTTGAGATTATCGCCAATGACCAGGGCAACCGCACCACG 105**

**Drosophila_Aa_AF295933 GGTGTCTACCAGCATGGCAAGGTGGAGATTATCGCCAACGACCAGGGCAACCGCACCACG 105**

**Spodoptera_FJ754276 GGCGTGTGGCAGCACGGCAACGTGGAGATCATCGCCAACGACCAGGGCAACCGCACCACA 105**

**Bombyx_AB035326 GGAGTATGGCAGCACGGGAACGTGGAGATCATCGCGAACGACCAGGGCAACCGTACCACA 105**

**Paralvinella_EF580992 GGTGTCTTCCAACATGGAAAAGTGGAGATTATTGCCAACGACCAAGGAAATAGGACGACA 120**

**B_plicatilis_HSP70-1_AB775784 GGCGTATTCCAGCACGGCAAAGTGGAAATAATCGCCAACGACCAAGGCAACCGCACAACC 114**

**B_plicatilis_HSP70-2_AB775785 GGCGTATTCCAGCACGGCAAAGTGGAAATAATCGCCAACGACCAAGGCAACCGCACAACC 114**

**A_brightwelli_HSP70_KX119429 GGCGTGTTCCAACATGGTAAAGTTGAGATCATTGCCAACGACCAAGGTAATAGAACAACG 111**

**Wuchereria_AF167352 GGTGTGTTCATGCACGGCAAAGTGGAAATCATTGCTAATGATCAAGGTAATCGTACAACA 111**

**Setaria_AF079360 GGTGTTTTCATGCACGGTAAAGTGGAAATCATTGCTAACGATCAAGGTAATCGAACAACA 111**

**C_elegans_HSP70A_M18540 GGAGTTTTCATGCACGGAAAGGTAGAAATCATTGCCAACGATCAAGGAAACCGTACAACT 114**

**C_elegans_HSP-1_NM_070667 GGAGTTTTCATGCACGGAAAGGTAGAAATCATTGCCAACGATCAAGGAAACCGTACAACT 114**

**M_nipponense_KC460343 GGTGTGTTCCAGCATGGAAAGGTAGAAATCATTGCCAATGATCAGGGCAACCGAACCACT 114**

**M_nipponense_DQ660140 GGTGTGTTCCAGCATGGAAAGGTAGAAATCATTGCCAATGATCAGGGCAACCGAACCACT 114**

**Mytilus_HSP70_AY861684 GGAGTATTCCAGCATGGCAAAGTTGAAATCATTGCCAATGATCAAGGAAACAGAACAACC 117**

**Perinereis_KU255783 GGGGTTTTCCAGCACGGGAAAGTGGAAATCATTGCAAACGACCAGGGTAACAGGACCACG 111**

**Paralvinella_HSP70-2_EF580993 GGTGTATTCCAGCATGGCAAGGTGGAAATCGTAGCTAATGATCAGGGAAACCGAACGACG 114**

**Haliotis_FJ812176 GGGGTATTTCAACACGGAAAGGTAGAAATCATTGCCAATGACCAAGGAAACAGAACAACA 114**

**Oncorhynchus_HSC71_AAB21658 GGTGTGTTCCAGCATGGCAAGGTTGAAATCATTGCCAACGACCAAGGCAACAGGACCACT 114**

**X_maculatus_HSC70_AB062115 GGAGTGTTTCAGCACGGGAAAGTTGAGATCATTGCTAATGACCAGGGGAACAGGACCACG 114**

**Human_HSPA8_HSC71_NP_006588 GGTGTTTTCCAGCACGGAAAAGTCGAGATAATTGCCAATGATCAGGGAAACCGAACCACT 114**

**Rattus_HSC71_NM_024351 GGTGTCTTCCAGCATGGAAAGGTGGAAATAATTGCCAATGACCAGGGTAACCGCACCACG 114**

**Pelodiscus_HSC70_HQ219723 GGGGTCTTCCAACATGGCAAGGTGGAGATCATTGCCAATGATCAGGGCAACAGGACCACG 114**

**Bos_HSP70_U09861 GGGGTGTTCCAGCACGGCAAGGTGGAGATCATCGCCAACGACCAGGGCAACCGCACCACC 114**

**Human_HSPA1A_NM_005345 GGGGTGTTCCAACACGGCAAGGTGGAGATCATCGCCAACGACCAGGGCAACCGCACCACC 114**

**Rattus_HSP70_1A_NM_031971 GGCGTGTTCCAGCACGGCAAGGTGGAGATCATCGCCAACGACCAGGGCAACCGCACGACC 114**

**X_maculatus_HSP70-1_AB062113 GGGGTCTTCCAGCATGGAAAAGTGGAAATTATCGCCAATGATCAAGGCAACAGGACAACT 120**

**X_maculatus_HSP70-2_AB062114 GGGGTTTTCCAGCACGGAAAAGTGGAAATCATCGCCAACGACCAGGGCAACAGGACCACC 120**

**Human_HSPA1L_NM_005527 GGGGTGTTCCAGCACGGCAAGGTGGAGATCATCGCCAACGACCAGGGCAACCGCACCACC 120**

**Human_HSPA6_NP_002146 GGCGTGTTTCAGCAGGGCCGCGTGGAGATCCTGGCCAACGACCAGGGCAACCGCACCACG 120**

**Alligator_HSP70_AB306279 GGCGTCTTCCAGCATGGCAAGGTGGAGATCATTGCCAACGACCAGGGCAACCGCACCACG 117**

**** ** * ** ** ** ** ** * ** ** ** ** ** ** * ** ****

**Drosophila_Bb_AF295957 CCGTCCTACGTGGCTTTCACAGACTCGGAACGCCTCATTGGTGATCCGGCCAAGAACCAG 165**

**Drosophila_Aa_AF295933 CCGTCCTACGTGGCTTTCACAGATTCGGAACGCCTCATCGGCGATCCGGCTAAGAACCAG 165**

**Spodoptera_FJ754276 CCATCCTATGTGGCGTTCACGGACACGGAGCGCCTCATCGGAGACGCAGCCAAGAACCAG 165**

**Bombyx_AB035326 CCATCGTACGTCGCGTTCACGGACACGGAGCGTCTCATCGGCGACGCAGCCAAGAACCAG 165**

**Paralvinella_EF580992 CCAAGTTATGTGGCATTTACAGATACCGAGAGACTGATAGGTGATGCGGCAAAGAACCAA 180**

**B_plicatilis_HSP70-1_AB775784 CCCTCCTACGTAGCGTTCACCGACACCGAGCGCTTGATCGGCGACGCGGCCAAAAACCAA 174**

**B_plicatilis_HSP70-2_AB775785 CCCTCCTACGTAGCGTTCACCGACACCGAGCGCTTGATCGGCGACGCGGCCAAAAACCAA 174**

**A_brightwelli_HSP70_KX119429 CCGAGTTACGTGGCGTTCACTGATACTGAACGTTTGATCGGAGACGCGGCCAAGAACCAA 171**

**Wuchereria_AF167352 CCATCGTATGTGGCATTTACCGATACTGAACGTCTTATTGGCGATGCAGCCAAAAATCAG 171**

**Setaria_AF079360 CCATCATATGTGGCATTTACCGATACTGAACGTCTTATTGGTGATGCAGCCAAAAATCAG 171**

**C_elegans_HSP70A_M18540 CCATCATATGTGGCTTTCACCGACACCGAGCGTCTCATCGGAGATGCTGCCAAGAATCAA 174**

**C_elegans_HSP-1_NM_070667 CCATCATATGTGGCTTTCACCGACACCGAGCGTCTCATCGGAGATGCTGCCAAGAATCAA 174**

**M_nipponense_KC460343 CCATCATATGTTGCTTTCACCGATACAGAGCGTCTTATTGGTGATGCTGCCAAGAACCAA 174**

**M_nipponense_DQ660140 CCATCATATGTTGCTTTCACCGACACAGAGCGTCTTATTGGTGATGCTGCCAAGAACCAA 174**

**Mytilus_HSP70_AY861684 CCAAGCTATGTCGCCTTCACAGACACCGAAAGATTAATAGGTGATGCTGCCAAGAACCAA 177**

**Perinereis_KU255783 CCCAGTTATGTGGCATTCACAGACTCCGAGCGTCTCATTGGAGATGCCGCAAAGAACCAA 171**

**Paralvinella_HSP70-2_EF580993 CCAAGTTATGTGGCGTTTACGGATTCCGAACGTATTATTGGAGATGGAGCCAAGAACCAA 174**

**Haliotis_FJ812176 CCCAGCTATGTTGCCTTCACCGACACAGAGCGTCTCATTGGTGACGCTGCAAAGAACCAG 174**

**Oncorhynchus_HSC71_AAB21658 CCAAGCTACGTTGCCTTCACTGACTCTGAGAGGCTCATCGGTGATGCTGCCAAGAATCAG 174**

**X_maculatus_HSC70_AB062115 CCCAGTTATGTGGCCTTTACTGACACAGAGAGGCTGATTGGAGATGCAGCCAAGAACCAG 174**

**Human_HSPA8_HSC71_NP_006588 CCAAGCTATGTCGCCTTTACGGACACTGAACGGTTGATCGGTGATGCCGCAAAGAATCAA 174**

**Rattus_HSC71_NM_024351 CCGAGCTATGTTGCTTTCACCGACACAGAACGATTAATTGGGGATGCGGCCAAGAATCAG 174**

**Pelodiscus_HSC70_HQ219723 CCAAGTTACGTTGCCTTCACAGATACAGAGAGGTTGATCGGTGATGCTGCAAAGAACCAA 174**

**Bos_HSP70_U09861 CCCAGCTACGTGGCCTTCACCGATACCGAGCGGCTCATCGGCGATGCGGCCAAGAACCAG 174**

**Human_HSPA1A_NM_005345 CCCAGCTACGTGGCCTTCACGGACACCGAGCGGCTCATCGGGGATGCGGCCAAGAACCAG 174**

**Rattus_HSP70_1A_NM_031971 CCCAGCTACGTGGCCTTCACCGACACCGAGCGGCTCATCGGGGACGCCGCCAAGAACCAG 174**

**X_maculatus_HSP70-1_AB062113 CCCAGCTACGTGGCCTTTACCGACACAGAGAGGCTCATCGGAGATGCTGCCAAGAACCAA 180**

**X_maculatus_HSP70-2_AB062114 CCCAGCTACGTGGCCTTCACCGACACCGAGAGGCTGATCGGGGACGCGGCCAAGAACCAG 180**

**Human_HSPA1L_NM_005527 CCCAGCTACGTGGCCTTCACAGACACCGAGCGGCTCATTGGGGATGCGGCCAAGAACCAG 180**

**Human_HSPA6_NP_002146 CCCAGCTACGTGGCCTTCACCGACACCGAGCGGCTGGTCGGGGACGCGGCCAAGAGCCAG 180**

**Alligator_HSP70_AB306279 CCCAGCTACGTGGCCTTCACGGACACGGAGCGCCTCATCGGCGACGCGGCCAAGAACCAG 177**

**** ** ** ** ** ** ** * ** * * * ** ** ** ** * ****

**Drosophila_Bb_AF295957 GTGGCCATGAACCCCAGAAACACAGTGTTTGACGCCAAGCGACTCATCGGCCGAAAATAC 225**

**Drosophila_Aa_AF295933 GTGGCCATGAACCCCAGAAACACAGTGTTTGACGCCAAGCGACTCATCGGCCGAAAATAC 225**

**Spodoptera_FJ754276 GTCGCCCTCAACCCCAACAACACTGTGTTCGACGCCAAGCGACTGATCGGAAGGAAATTC 225**

**Bombyx_AB035326 GTCGCCTTGAACCCTAACAACACCGTGTTCGACGCGAAGGGGCTGATCGGGAGGAAATTC 225**

**Paralvinella_EF580992 GTGGCGCTGAATCCGAGCAACACGGTATTTGATGCGAAGAGGCTGATTGGTCGAAGATTT 240**

**B_plicatilis_HSP70-1_AB775784 GTGGCCATGAACCCCAATAACACGGTGTTCGACGCCAAGCGTCTCATCGGCCGCAAGTTC 234**

**B_plicatilis_HSP70-2_AB775785 GTGGCCATGAACCCCAATAACACGGTGTTCGACGCCAAGCGTCTCATCGGCCGCAAGTTC 234**

**A_brightwelli_HSP70_KX119429 GTGGCCATGAATCCGACGAACACAGTGTTCGACGCGAAACGTCTCATCGGACGCAGATTC 231**

**Wuchereria_AF167352 GTTGCAATGAATCCGCACAACACTGTCTTCGACGCTAAACGACTGATTGGTCGTAAGTTT 231**

**Setaria_AF079360 GTTGCGATGAATCCGCACAATACTGTTTTCGATGCGAAACGATTGATCGGCCGTAAATTT 231**

**C_elegans_HSP70A_M18540 GTTGCCATGAACCCACATAACACTGTTTTCGATGCCAAACGTCTTATTGGACGCAAGTTC 234**

**C_elegans_HSP-1_NM_070667 GTTGCCATGAACCCACATAACACTGTTTTCGATGCCAAACGTCTTATTGGACGCAAGTTC 234**

**M_nipponense_KC460343 GTTGCTATGAATCCCAACAACACTGTATTTGATGCCAAGAGGCTCATTGGTCGTAAGTTT 234**

**M_nipponense_DQ660140 GTTGCTATGAATCCCAACAACACTGTATTTGATGCCAAGAGGCTCATTGGTCGTAAGTTT 234**

**Mytilus_HSP70_AY861684 GTGGCAATGAACCCAGTCAACACGGTTTTTGATGCCAAGAGATTAATTGGTAGAAAGTTT 237**

**Perinereis_KU255783 GTGGCCATGAACCCCGAGAACACAGTCTTCGACGCCAAACGTTTGATCGGACGCAAGTTT 231**

**Paralvinella_HSP70-2_EF580993 GTGGCCATGAACCCCAGTAACACGGTGTTCGATGCGAAACGTCTCATTGGTCGTAGGCTC 234**

**Haliotis_FJ812176 GTTGCCATGAACCCAGAGAACACTATCTTCGATGCCAAACGTCTGATTGGTAGAAGATTT 234**

**Oncorhynchus_HSC71_AAB21658 GTTGCCATGAACCCCTGCAACACAGTATTCGATGCTAAGAGACTGATTGGCCGCAGGTTT 234**

**X_maculatus_HSC70_AB062115 GTGGCCCTCAACCCCAACAACACCGTTTTTGATGCAAAGCGGCTTATTGGGCGTCGTTTT 234**

**Human_HSPA8_HSC71_NP_006588 GTTGCAATGAACCCCACCAACACAGTTTTTGATGCCAAACGTCTGATTGGACGCAGATTT 234**

**Rattus_HSC71_NM_024351 GTTGCAATGAACCCCACCAACACAGTTTTTGATGCCAAACGTCTGATCGGACGTAGGTTC 234**

**Pelodiscus_HSC70_HQ219723 GTTGCAATGAATCCTACCAACACGGTTTTTGATGCAAAGCGGCTGATCGGTCGTAGATTT 234**

**Bos_HSP70_U09861 GTGGCGCTGAACCCGCAGAACACGGTGTTCGACGCGAAGCGGCTGATCGGCCGCAAGTTC 234**

**Human_HSPA1A_NM_005345 GTGGCGCTGAACCCGCAGAACACCGTGTTTGACGCGAAGCGGCTGATTGGCCGCAAGTTC 234**

**Rattus_HSP70_1A_NM_031971 GTGGCGCTGAACCCGCAGAACACCGTGTTCGACGCGAAGCGGCTGATCGGCCGCAAGTTC 234**

**X_maculatus_HSP70-1_AB062113 GTTGCCATGAACCCCACCAACACGATCTTTGATGCCAAACGGCTCATTGGAAGAAGGTTT 240**

**X_maculatus_HSP70-2_AB062114 GTGGCCCTGAACCCCAGCAACACGGTGTTTGACGCCAAGAGGCTGATCGGCAGAAAGTTT 240**

**Human_HSPA1L_NM_005527 GTAGCAATGAATCCCCAGAACACTGTTTTTGATGCTAAACGTCTGATCGGCAGGAAATTT 240**

**Human_HSPA6_NP_002146 GCGGCCCTGAACCCCCACAACACCGTGTTCGATGCCAAGCGGCTGATCGGGCGCAAGTTC 240**

**Alligator_HSP70_AB306279 GTGGCCATGAACCCCACCAACACCATCTTCGACGCCAAGCGCCTCATCGGCCGCAAGTAC 237**

*** ** * ** ** ** ** * ** ** ** ** * * ** ** ***

**Drosophila_Bb_AF295957 GACGATCCCAAAATCGCAGAGGACATGAAGCACTGGCCTTTCAAAGTTGTAAGCGACG-- 283**

**Drosophila_Aa_AF295933 GACGACCCCAAGATCGCAGAGGACATGAAGCACTGGCCTTTCAAGGTGGTAAGCGACG-- 283**

**Spodoptera_FJ754276 GATGACCCCAAGATCCAGGCAGACATGAAGCACTGGCCCTTCAGGGTGGTCAGCGACT-- 283**

**Bombyx_AB035326 GACGACCCCAAGATTCAGCAGGACATGAAGCACTGGCCCTTCAAAGTAATCAACGACT-- 283**

**Paralvinella_EF580992 GATGACGACAATGTCCAGAAGGACATCAAACATTGGCCTTTTAAAGTACTTAACGACG-- 298**

**B_plicatilis_HSP70-1_AB775784 GACGACCTGACCGTCCAGGCCGACATGAAGCACTGGCCCTTCACGGTGATCAGCGACA-- 292**

**B_plicatilis_HSP70-2_AB775785 GACGACCCGACCGTCCAGGCCGACATGAAGCACTGGCCCTTCACGGTGATCAGCGACA-- 292**

**A_brightwelli_HSP70_KX119429 GACGACTCCACTGTCCAGTCCGACATGAAACACTGGCCGTTCACTGTCGTCAATGAAG-- 289**

**Wuchereria_AF167352 GATGATGGATCAGTGCAATCGGATATGAAACATTGGCCATTTAAAGTAGTGAATGCTGGT 291**

**Setaria_AF079360 GATGATGGATCAGTACAGTCAGACATGAAACATTGGCCGTTTAAAGTTATGAATGCTGGT 291**

**C_elegans_HSP70A_M18540 GACGATCCAGCAGTTCAGTCTGACATGAAGCATTGGCCATTCAAGGTCATCTCTGCCGAA 294**

**C_elegans_HSP-1_NM_070667 GACGATCCAGCAGTTCAGTCTGACATGAAGCATTGGCCATTCAAGGTCATCTCTGCCGAA 294**

**M_nipponense_KC460343 GAAGATCATGTAGTGCAGTCCGACATGAAACATTGGCCCTTTACAGTCATTAATGACA-- 292**

**M_nipponense_DQ660140 GAAGATCATGTAGTGCAGTCCGACATGAAACATTGGCCCTTTACAGTCATTAATGACA-- 292**

**Mytilus_HSP70_AY861684 GATGACGCTACAGTACAATCAGACATGAAGCATTGGCCTTTCACTGTTGTCAATGATG-- 295**

**Perinereis_KU255783 GACGACTCAGCCGTTCAGTCAGACAAAAAGCATTGGCCATTCGATGTGGTCAGTGAGG-- 289**

**Paralvinella_HSP70-2_EF580993 GATGATTCGTCCGTCCAATCAGATATGAAGTTCTGGCCCTTCAAGGTCATCAGTGAGA-- 292**

**Haliotis_FJ812176 GATGAGACAAATGTTCAATCAGACATGAAGCACTGGCCATTCAATGTGTTGAGTGATG-- 292**

**Oncorhynchus_HSC71_AAB21658 GATGATGGAGTTGTTCAATCGGACATGAAGCATTGGCCCTTTGAAGTTATCAATGATT-- 292**

**X_maculatus_HSC70_AB062115 GACGATAGTATTGTCCAATCAGACATGAAACATTGGCCCTTCACCATCATCAATGATA-- 292**

**Human_HSPA8_HSC71_NP_006588 GATGATGCTGTTGTCCAGTCTGATATGAAACATTGGCCCTTTATGGTGGTGAATGATG-- 292**

**Rattus_HSC71_NM_024351 GATGATGCTGTTGTTCAGTCTGACATGAAGCACTGGCCCTTCATGGTGGTGAACGATG-- 292**

**Pelodiscus_HSC70_HQ219723 GATGATGCTGTTGTCCAGTCGGACATGAAACATTGGCCATTCACTGTGGTGAATGATG-- 292**

**Bos_HSP70_U09861 GGAGACCCGGTGGTGCAGTCGGACATGAAGGAGTGGCCTTTCCGCGTCATCAACGACG-- 292**

**Human_HSPA1A_NM_005345 GGCGACCCGGTGGTGCAGTCGGACATGAAGCACTGGCCTTTCCAGGTGATCAACGACG-- 292**

**Rattus_HSP70_1A_NM_031971 GGCGACCCGGTGGTGCAGTCGGACATGAAGCACTGGCCCTTCCAGGTGGTGAACGACG-- 292**

**X_maculatus_HSP70-1_AB062113 GATGACCTGGTGGTCCAGTCTGACATGAAACTCTGGCCGTTCAAGGTGATCAACGACA-- 298**

**X_maculatus_HSP70-2_AB062114 GAAGAGCCAGTGGTGCAGGCCGACATGAAGCACTGGCCCTTCGAGGTGCTTTCAGACG-- 298**

**Human_HSPA1L_NM_005527 AATGATCCTGTTGTACAAGCAGATATGAAACTTTGGCCTTTTCAAGTGATTAATGAAG-- 298**

**Human_HSPA6_NP_002146 GCGGACACCACGGTGCAGTCGGACATGAAGCACTGGCCCTTCCGGGTGGTGAGCGAGG-- 298**

**Alligator_HSP70_AB306279 GACGACCCCACGGTGCAGGCGGACATGAAGCACTGGCCCTTCCACGTAGTGTCCGAGG-- 295**

**** * ** * ** ***** ** * * ***

**Drosophila_Bb_AF295957 -GCGGAAAGCCCAAGATCGGGGTGGAGTATAAGGGTGAGTCCAAGAGATTTGCTCCCGAG 342**

**Drosophila_Aa_AF295933 -GCGGAAAGCCCAAGATCGGGGTGGAGTATAAGGGTGAGTCCAAGAGATTTGCCCCCGAG 342**

**Spodoptera_FJ754276 -GTGGCAAACCGAAGATCCAAGTGGAGTTCAAGGGTGAAACGAAACGGTTCGCGCCCGAG 342**

**Bombyx_AB035326 -GCGGCAAACCGAAAATACAGATCGAGTTCAAAGGTGAGACGAAACGATTTGCGCCAGAA 342**

**Paralvinella_EF580992 -GTGGAAAGCCAAAGATTGACGTAGAATACAAAAGTGAGAGGAAACGTTTTACTCCAGAA 357**

**B_plicatilis_HSP70-1_AB775784 -GCGGCCGGCCCAAAATCCAGGTCGAGTTCAAAGGCGAGACCAAGAGCTTCTACCCGGAA 351**

**B_plicatilis_HSP70-2_AB775785 -GCGGCCGGCCCAAAATCCAGGTCGAGTTCAAAGGCGAGACCAAGAGCTTCTACCCGGAA 351**

**A_brightwelli_HSP70_KX119429 -GCGGAAAACCAAAAATCCAAGTCGAGTTCAAAGGCGAGAAAAAGACCTTCTTCCCTGAA 348**

**Wuchereria_AF167352 GGTGGTAAACCTAAAGTGCAGGTTGAATACAAGGGTGAGACAAAGACCTTTACTCCAGAA 351**

**Setaria_AF079360 GGAGGCAAGCCAAAAGTTCAAGTGGAGTATAAGGGGGAGACGAAAACTTTCACGCCCGGA 351**

**C_elegans_HSP70A_M18540 GGAGCTAAGCCAAAGGTCCAAGTTGAGTACAAAGGAGAGAACAAGATCTTCACTCCAGAA 354**

**C_elegans_HSP-1_NM_070667 GGAGCTAAGCCAAAGGTCCAAGTTGAGTACAAAGGAGAGAACAAGATCTTCACTCCAGAA 354**

**M_nipponense_KC460343 -GCACGAAGCCCAAAATTCAAGTTGATTACAAAGGAGAGACCAAAACCTTTTTCCCAGAA 351**

**M_nipponense_DQ660140 -GCACGAAGCCCAAAATTCAAGTTGATTACAAAGGAGAGACCAAAACCTTTTTCCCAGAA 351**

**Mytilus_HSP70_AY861684 -CCTCCAAACCTAAAGTTACGGTAGACTACAAAGGAGAAACAAAAACATTTTTCCCTGAG 354**

**Perinereis_KU255783 -GTGGCAAACCAAAGATCTCTGTTGATTACAAGGGGGAGAAGAAATCATTTTACCCAGAA 348**

**Paralvinella_HSP70-2_EF580993 -ATGGCAAGCCAAAAATCCAAGTGGAGTACAAGGGGGAGTTGAAGACCTTCTATCCCGAA 351**

**Haliotis_FJ812176 -GAGGCAAACCCAAGATCCAAGTAAATTACAAAGATGAACCAAAAACTTTCTACCCTGAA 351**

**Oncorhynchus_HSC71_AAB21658 -CTACTCGGCCTAAGCTCCAAGTTGAATACAAAGGAGAGACTAAGTCCTTCTACCCAGAA 351**

**X_maculatus_HSC70_AB062115 -GCTCGAGGCCCAAAGTGAAAGTGGAGTACAAAGGGGAGACGAAGACCTTCTACCCAGAG 351**

**Human_HSPA8_HSC71_NP_006588 -CTGGCAGGCCCAAGGTCCAAGTAGAATACAAGGGAGAGACCAAAAGCTTCTATCCAGAG 351**

**Rattus_HSC71_NM_024351 -CAGGCAGGCCCAAGGTCCAAGTCGAATACAAAGGGGAGACAAAAAGTTTCTATCCTGAG 351**

**Pelodiscus_HSC70_HQ219723 -CTGGCAGGCCGAAAGTCCAGGTTGAGTACAAAGGGGAGACCAAGAGCTTCTATCCAGAG 351**

**Bos_HSP70_U09861 -GAGACAAGCCTAAGGTGCAGGTGAGCTACAAAGGGGAGACCAAGGCGTTCTACCCGGAG 351**

**Human_HSPA1A_NM_005345 -GAGACAAGCCCAAGGTGCAGGTGAGCTACAAGGGGGAGACCAAGGCATTCTACCCCGAG 351**

**Rattus_HSP70_1A_NM_031971 -GCGACAAGCCCAAGGTGCAGGTGAACTACAAGGGCGAGAACCGGTCGTTCTACCCGGAG 351**

**X_maculatus_HSP70-1_AB062113 -ACGGAAAGCCCAAAGTCCAGGTGGAATATAAAGGAGAAATTAAGACATTCTGTCCTGAA 357**

**X_maculatus_HSP70-2_AB062114 -GAGGCAGGCCCAAAATTCAGGTGGAGTACAAAGGGGAGAACAAAGCCTTCTTCCCTGAG 357**

**Human_HSPA1L_NM_005527 -GAGGCAAGCCCAAAGTCCTTGTGTCCTACAAAGGGGAGAATAAAGCTTTCTACCCTGAG 357**

**Human_HSPA6_NP_002146 -GCGGCAAGCCCAAGGTGCGCGTATGCTACCGCGGGGAGGACAAGACGTTCTACCCCGAG 357**

**Alligator_HSP70_AB306279 -GCGGCAAGCCCAAGGTGCAGGTGGAGTATAAGGGCGAGGCCAAGACCTTCTTCCCCGAA 354**

**** ** * * * ** ** ** ***

**Drosophila_Bb_AF295957 GAGATCAGTTCGATGGTGCTGACCAAGATGAAGGAGACGGCGGAGGCGTATCTGGGCGAG 402**

**Drosophila_Aa_AF295933 GAGATCAGCTCGATGGTGCTGACCAAGATGAAGGAGACGGCGGAGGCATATCTGGGCGAG 402**

**Spodoptera_FJ754276 GAGATCAGCAGCATGGTGCTGACGAAGATGAAGGAGACGGCGGAAGCTTACCTCGGAACG 402**

**Bombyx_AB035326 GAAATTAGCAGCATGGTGCTGACAAAAATGAAGGAGACGGCGGAAGCCTATCTGGGAAGT 402**

**Paralvinella_EF580992 GAAATCAGCTCGATGATACTTACCAAGATGAAAGAGACGGCCGAGGCCTATCTCGGTAAT 417**

**B_plicatilis_HSP70-1_AB775784 GAAGTCTCATCCATGGTACTGACCAAAATGAAGGAGACGGCCGAGGCCTACCTGGGCAAA 411**

**B_plicatilis_HSP70-2_AB775785 GAAGTCTCATCCATGGTACTGACCAAAATGAAGGAGACGGCCGAGGCCTACCTGGGCAAA 411**

**A_brightwelli_HSP70_KX119429 GAAATCTCATCAATGGTCTTAATTAAAATGAAAGAGATCGCCGAGGCTTATTTGGGAAAG 408**

**Wuchereria_AF167352 GAGATTTCGTCGATGGTTTTGGTTAAAATGAAGGAGACGGCTGAAGCTTTTCTGGGTCAT 411**

**Setaria_AF079360 GAAATTTCTTCAATGGTTTTAGTTAAAATGAAAGAAACAGCTGAGGCATTTCTTGGTCAC 411**

**C_elegans_HSP70A_M18540 GAGATCTCCTCAATGGTTCTGCTGAAGATGAAGAAGACTGCCGAGGCTTTCCTTGAACCG 414**

**C_elegans_HSP-1_NM_070667 GAGATCTCCTCAATGGTTTTGCTGAAGATGAAGGAGACTGCCGAGGCTTTCCTTGGAACC 414**

**M_nipponense_KC460343 GAGATCTCCTCAATGGTGCTTATTAAGATGAAAGAAACTGCAGAAGCCTTTTTGGGTGGT 411**

**M_nipponense_DQ660140 GAGATCTCCTCAATGGTGCTTATTAAGATGAAAGAAACTGCAGAAGCCTTTTTGGGTGGT 411**

**Mytilus_HSP70_AY861684 GAAATTTCATCAATGGTGCTGGTAAAAATGAAAGAAACTGCAGAAGCATATCTAGGAAAG 414**

**Perinereis_KU255783 GAGATCTCCTCCATGGTATTGGTTAAGATGAAGGAAACAGCCGAGGCTTACATCGGCAAG 408**

**Paralvinella_HSP70-2_EF580993 GAAATTTCCTCCATGGTACTTTTGAAAATGAAAGAAACTGCTGAAGCCTATCTTGGAAAG 411**

**Haliotis_FJ812176 GAGATTTCCTCCATGGTGCTAACAAAGATGAAGGAAACTGCAGAACAGTATTTGGGAAAG 411**

**Oncorhynchus_HSC71_AAB21658 GAAATTTCATCTATGGTTCTGGTCAAGATGAAGGAGATTGCTGAGGCCTACCTTGGGAAA 411**

**X_maculatus_HSC70_AB062115 GAGATCTCCTCCATGGTGCTGCTGAAGATGAAGGAGATTGCTGAGGCATACCTTGGCAAG 411**

**Human_HSPA8_HSC71_NP_006588 GAGGTGTCTTCTATGGTTCTGACAAAGATGAAGGAAATTGCAGAAGCCTACCTTGGGAAG 411**

**Rattus_HSC71_NM_024351 GAAGTGTCTTCAATGGTTCTGACAAAAATGAAGGAAATTGCAGAAGCTTACCTTGGAAAG 411**

**Pelodiscus_HSC70_HQ219723 GAAATATCTTCCATGGTTTTGACAAAGATGAAAGAGATAGCAGAAGCATATCTTGGGAAG 411**

**Bos_HSP70_U09861 GAGATCTCGTCGATGGTGCTGACCAAGATGAAGGAGATCGCCGAGGCGTACCTGGGCCAC 411**

**Human_HSPA1A_NM_005345 GAGATCTCGTCCATGGTGCTGACCAAGATGAAGGAGATCGCCGAGGCGTACCTGGGCTAC 411**

**Rattus_HSP70_1A_NM_031971 GAGATCTCGTCCATGGTGCTGACCAAGATGAAGGAGATCGCCGAGGCGTACCTGGGCCAC 411**

**X_maculatus_HSP70-1_AB062113 GAGATTTCCTCGATGGTCCTGGTTAAAATGAGAGAAATAGCTGAGGCCTTTCTGGGACAG 417**

**X_maculatus_HSP70-2_AB062114 GAGATCTCCTCCATGGTCCTGGTGAAGATGAAGGAAATCGCCGAGGCCTACCTGGGCCAC 417**

**Human_HSPA1L_NM_005527 GAAATCTCTTCGATGGTATTGACTAAGTTGAAGGAGACTGCTGAGGCCTTTTTGGGCCAC 417**

**Human_HSPA6_NP_002146 GAGATCTCGTCCATGGTGCTGAGCAAGATGAAGGAGACGGCCGAGGCGTACCTGGGCCAG 417**

**Alligator_HSP70_AB306279 GAGATCAGCTCCATGGTGCTGACTAAGATGAAGGAGATCGCCGAAGCCTACCTGGGCCGC 414**

**** * *** * * ** *** * * ** ** * * ***

**Drosophila_Bb_AF295957 AGCATCACGGATGCAGTCATCACAGTTCCAGCTTACTTCAACGACTCTCAGCGCCAGGCT 462**

**Drosophila_Aa_AF295933 AGCATCACAGACGCAGTCATCACAGTTCCAGCCTACTTCAACGACTCCCAGCGCCAGGCT 462**

**Spodoptera_FJ754276 ACAGTACGCGACGCAGTGATCACAGTGCCGGCGTACTTCAACGACTCTCAGCGCCAGGCC 462**

**Bombyx_AB035326 ACAGTGCGGGATGCGGTAGTCACAGTTCCGGCATACTTCAACGACTCCCAGCGTCAGGCC 462**

**Paralvinella_EF580992 AAAGTGTTAGATGCCGTCATAACTGTCCCCGCTTACTTCAACGACTCTCAGAGACAAGCT 477**

**B_plicatilis_HSP70-1_AB775784 AAGGTCACGGACGCAGTGGTGACCGTGCCCGCCTACTTCAACGACTCGCAGCGCCAAGCG 471**

**B_plicatilis_HSP70-2_AB775785 AAGGTCACGGACGCAGTGGTGACCGTGCCCGCCTACTTCAACGACTCGCAGCGCCAAGCG 471**

**A_brightwelli_HSP70_KX119429 AAAGTAAACGACGCCGTGATCACAGTCCCGGCCTATTTCAACGACTCTCAAAGACAGGCC 468**

**Wuchereria_AF167352 GCGGTCAAGGATGCTGTTATTACAGTCCCCGCATATTTTAATGACTCACAGCGACAAGCC 471**

**Setaria_AF079360 GCAGTCAAGGATGCTGTGATCACAGTTCCAGCTTATTTCAATGACTCACAGCGACAGGCA 471**

**C_elegans_HSP70A_M18540 ACCGTCAAGGATGCCGTTGTCACTGTCCCGACTTACTTCAACGACTCGCAGCGTCAAGCC 474**

**C_elegans_HSP-1_NM_070667 ACCGTCAAGGATGCCGTTGTCACTGTCCCAGCTTACTTCAACGACTCGCAGCGTCAAGCC 474**

**M_nipponense_KC460343 ACTGTAAAGGATGCTGTCGTCACAGTCCCTGCCTATTTCAATGACTCCCAGCGTCAGGCC 471**

**M_nipponense_DQ660140 ACTGTAAAGGATGCTGTCGTCACAGTCCCTGCCTATTTCAATGACTCCCAGCGTCAGGCC 471**

**Mytilus_HSP70_AY861684 TTAGTCAACAACAGTGTCATTACAGTCCCAGCTTATTTTAATGATTCACAAGGACAAGCA 474**

**Perinereis_KU255783 ACTGTTCTAAATGCCGTCGTAACAGTGCCTGCCTACTTCAACGATTCTCAGCGCCAAGCA 468**

**Paralvinella_HSP70-2_EF580993 AACGTACAGAGCGCTGTGATCACAGTACCAGCCTATTTCAATGACAGCCAGCGTCAGGCC 471**

**Haliotis_FJ812176 ACTATAACAGACGCTGTTGTAACAGTCCCAGCTTACTTCAACGACTCTCAGCGACAGGCC 471**

**Oncorhynchus_HSC71_AAB21658 ACTGTCAACAATGCTGTTGTTACCGTACCTGCCTACTTCAATGACTCCCAGCGCCAGGCA 471**

**X_maculatus_HSC70_AB062115 ACTATAACAAATGCCGTGGTGACTGTACCTGCCTACTTCAATGACTCTCAGCGTACGGCC 471**

**Human_HSPA8_HSC71_NP_006588 ACTGTTACCAATGCTGTGGTCACAGTGCCAGCTTACTTTAATGACTCTCAGCGTCAGGCT 471**

**Rattus_HSC71_NM_024351 ACTGTTACCAATGCCGTGGTCACCGTGCCAGCTTACTTCAATGACTCTCAGCGACAGGCA 471**

**Pelodiscus_HSC70_HQ219723 ACTGTTACTAATGCAGTGGTCACGGTACCAGCCTACTTCAATGACTCCCAGCGCCAGGCC 471**

**Bos_HSP70_U09861 CCGGTGACCAACGCGGTGATCACCGTGCCGGCCTACTTCAACGACTCGCAGCGGCAGGCC 471**

**Human_HSPA1A_NM_005345 CCGGTGACCAACGCGGTGATCACCGTGCCGGCCTACTTCAACGACTCGCAGCGCCAGGCC 471**

**Rattus_HSP70_1A_NM_031971 CCGGTGACCAACGCGGTGATCACCGTGCCCGCCTACTTCAACGACTCGCAGCGGCAGGCC 471**

**X_maculatus_HSP70-1_AB062113 AGGGTGTCGAATGCGGTCATCACAGTGCCAGCTTACTTTAATGATTCCCAAAGGCAAGCC 477**

**X_maculatus_HSP70-2_AB062114 AAGGTGTCCAACGCCGTGATCACGGTCCCCGCCTATTTCAACGACTCCCAGCGACAGGCC 477**

**Human_HSPA1L_NM_005527 CCTGTCACCAATGCAGTGATTACCGTGCCAGCCTATTTCAATGACTCTCAACGTCAGGCT 477**

**Human_HSPA6_NP_002146 CCCGTGAAGCACGCAGTGATCACCGTGCCCGCCTATTTCAATGACTCGCAGCGCCAGGCC 477**

**Alligator_HSP70_AB306279 AAGGTGCAGAACGCCGTCATCGCGGTGCCCGCCTACTTCAACGACTCGCAGCGCCAGGCC 474**

*** ** * * ** ** * ** ** ** ** ** * ****

**Drosophila_Bb_AF295957 ACCAAAGACGCCGGTCACATCGCCGGCCTGAATGTGCTCCGCATCATCAATGAGCCCACG 522**

**Drosophila_Aa_AF295933 ACCAAAGACGCCGGTCACATCGCCGGCCTGAATGTGCTCCGCATCATCAATGAGCCCACG 522**

**Spodoptera_FJ754276 ACCAAGGACGCGGGAGCCATCGCCGGGCTGAACGTGCTCAGGATCATCAACGAGCCCACA 522**

**Bombyx_AB035326 ACCAAGGACGCCGGAGCCATCGCCGGCCTGAACGTGCTTCGCATCATCAACGAGCCCACA 522**

**Paralvinella_EF580992 ACAAAAGATGCTGGTCTCATCTCCGGATTGAACGTTCTCCGAGTGATTAACGAGCCAACA 537**

**B_plicatilis_HSP70-1_AB775784 ACCAAGGACGCGGGCGCCATCGCCGGCCTCAACGTGCTGCGCATCATCAACGAGCCCACT 531**

**B_plicatilis_HSP70-2_AB775785 ACCAAGGACGCGGGCGCCATCGCCGGCCTCAACGTGCTGCGCATCATCAACGAGCCCACT 531**

**A_brightwelli_HSP70_KX119429 ACAAAAGACGCCGGCGCCATTTCAGGTCTGAACGTGTTACGTATCATAAACGAGCCAACA 528**

**Wuchereria_AF167352 ACTAAAGATTCCGGTGCAATTGCTGGTCTAAATGTCTTGCGTATTATCAACGAACCAACA 531**

**Setaria_AF079360 ACGAAAGATTCGGGTGCCATTGCGGGTTTGAACGTTCTGCGTATTATCAACGAACCAACC 531**

**C_elegans_HSP70A_M18540 ACCAAGGATGCCGGAGCCATCGCTGGACTCAACGTTCTCCGTATCATCAACGAGCCAACC 534**

**C_elegans_HSP-1_NM_070667 ACCAAGGATGCCGGAGCCATCGCTGGACTCAACGTTCTCCGTATCATCAACGAGCCAACC 534**

**M_nipponense_KC460343 ACCAAAGATGCTGGTACAATCTCTGGTCTCAATGTGCTCCGTATCATCAATGAGCCCACT 531**

**M_nipponense_DQ660140 ACCAAAGATGCTGGTACAATCTCTGGTCTCAATGTGCTCCGTATCATCAATGAGCCCACT 531**

**Mytilus_HSP70_AY861684 ACAAAGGATGCTGGTACCATCTCTGGAATGAATGTTCTACGTATTATCAATGAGCCAACT 534**

**Perinereis_KU255783 ACCAAAGATGCCGGAACTATCTCTGGCTTGAATGTATTACGTATCATCAATGAACCAACG 528**

**Paralvinella_HSP70-2_EF580993 ACCAAAGATGCTGGTACCATCTCTGGTATGAATGTGCTGCGTATCATCAATGAGCCCACA 531**

**Haliotis_FJ812176 ACTAAAGATGCAGGGACAATCTCTGGTCTCAATGTTTTACGTATCATCAATGAGCCTACT 531**

**Oncorhynchus_HSC71_AAB21658 ACCAAAGATGCTGGTACCATCTCGGGGCTGAATGTGCTGCGTATCATCAATGAGCCAACT 531**

**X_maculatus_HSC70_AB062115 ACCAAAGACGCTGGGACCATTTCTGGGCTCAATGTTCTGCGTATCATTAACGAGCCAACG 531**

**Human_HSPA8_HSC71_NP_006588 ACCAAAGATGCTGGAACTATTGCTGGTCTCAATGTACTTAGAATTATTAATGAGCCAACT 531**

**Rattus_HSC71_NM_024351 ACAAAAGATGCTGGAACTATTGCTGGCCTCAACGTACTTCGAATTATCAATGAGCCAACT 531**

**Pelodiscus_HSC70_HQ219723 ACAAAAGATGCTGGAACCATTGCAGGTCTTAATGTGCTCAGGATCATCAATGAACCAACT 531**

**Bos_HSP70_U09861 ACCAAGGACGCGGGGGTGATCGCGGGGCTGAACGTGCTGAGGATCATCAACGAGCCCACG 531**

**Human_HSPA1A_NM_005345 ACCAAGGATGCGGGTGTGATCGCGGGGCTCAACGTGCTGCGGATCATCAACGAGCCCACG 531**

**Rattus_HSP70_1A_NM_031971 ACCAAGGACGCGGGCGTGATCGCGGGTCTGAACGTGCTGCGGATCATCAACGAGCCCACG 531**

**X_maculatus_HSP70-1_AB062113 ACAAAGGATGCTGGAGTGATCTCCGGACTAAATGTTCTCCGCATCATAAATGAGCCGACA 537**

**X_maculatus_HSP70-2_AB062114 ACCAAAGACGCAGGCGTCATTGCGGGACTCAACGTCTTGAGGATCATCAACGAGCCCACG 537**

**Human_HSPA1L_NM_005527 ACTAAGGATGCAGGTGTGATTGCTGGACTTAATGTGCTAAGAATCATCAATGAGCCCACG 537**

**Human_HSPA6_NP_002146 ACCAAGGACGCGGGGGCCATCGCGGGGCTCAACGTGTTGCGGATCATCAATGAGCCCACG 537**

**Alligator_HSP70_AB306279 ACCAAGGACGCGGGCACCATCACGGGCCTCAACGTGATGCGCATCATCAACGAGCCCACG 534**

**** ** ** * ** ** * ** * ** ** * * * ** ** ** ** ****

**Drosophila_Bb_AF295957 GCGGCAGCATTGGCCTACGGACTGGACAAGAACCTCAA------GGGTGAGCGCAATGTG 576**

**Drosophila_Aa_AF295933 GCGGCAGCACTGGCCTACGGACTGGACAAGAACCTCAA------GGGTGAGCGCAATGTG 576**

**Spodoptera_FJ754276 GCCGCTGCGCTCGCCTACGGCCTCGACAAGAACCTCAA------GGGCGAGAGGAACGTC 576**

**Bombyx_AB035326 GCCGCCGCGCTCGCGTACGGTCTCGACAAGAACCTCAA------GGGTGAGAGAAATGTT 576**

**Paralvinella_EF580992 GCAGCCGCTCTGGCTTATGGCTTGGATAAGAACCTATC------TGGAGAGAAGAACGTC 591**

**B_plicatilis_HSP70-1_AB775784 GCGGCCGCCATCGCCTACGGCCTTGACAAAAAGGGCGG------CGGCGAAAAAAACATC 585**

**B_plicatilis_HSP70-2_AB775785 GCGGCCGCCATCGCCTACGGCCTTGACAAAAAGGGCGG------CGGCGAAAAAAACATC 585**

**A_brightwelli_HSP70_KX119429 GCAGCTGCGATCGCTTACGGTCTTGATAAAAAAGTCGG------CGGTGAGAAAAACGTG 582**

**Wuchereria_AF167352 GCAGCTGCTATTGCTTATGGTTTGGACAAGAAGGGCCA------TGGTGAACGCAACGTT 585**

**Setaria_AF079360 GCAGCAGCTATTGCATATGGGTTGGACAAGAAGGGCCA------TGGTGAGCGTAATGTC 585**

**C_elegans_HSP70A_M18540 GCTGCAGCTATCGCTTACGGACTTGACAAGAAGGGACA------CGGAGAACGCAACGTT 588**

**C_elegans_HSP-1_NM_070667 GCTGCAGCTATCGCTTACGGACTTGACAAGAAGGGACA------CGGAGAACGCAACGTT 588**

**M_nipponense_KC460343 GCTGCTGCCATCGCATATGGGCTGGACAAGAAAGTTGG------TGGAGAACGTAATGTT 585**

**M_nipponense_DQ660140 GCTGCTGCCATCGCATATGGGCTGGACAAGAAAGTTGG------TGGAGAACGTAATGTT 585**

**Mytilus_HSP70_AY861684 GCAGCTGCTATTGCTTATGGTTTGGATAAGAAAGTAGG------TGGAGAAAGAAATGTA 588**

**Perinereis_KU255783 GCCGCCGCCATCGCTTACGGTCTTGATAAGAAGGTGGG------AGGAGAGAGAAATGTC 582**

**Paralvinella_HSP70-2_EF580993 GCTGCCGCCATTGCTTACGGTTTGGACAAGAAGGTTGG------TGGAGAGCGTCATGTG 585**

**Haliotis_FJ812176 GCCGCAGCTATTGCATACGGTCTTGACAAGAAGGTTGG------TGGTGAACGCAACGTT 585**

**Oncorhynchus_HSC71_AAB21658 GCTGCTGCCATTGCCTACGGCCTGGACAAGAAGGTCGG------TGCTGAAAGGAATGTC 585**

**X_maculatus_HSC70_AB062115 GCTGCTGCCATTGCTTACGGTTTGGACAAAAAGGTCGG------GTGCGAGAAAAACGTC 585**

**Human_HSPA8_HSC71_NP_006588 GCTGCTGCTATTGCTTACGGCTTAGACAAAAAGGTTGG------AGCAGAAAGAAACGTG 585**

**Rattus_HSC71_NM_024351 GCTGCTGCTATTGCCTATGGCTTAGATAAGAAGGTCGG------GGCTGAAAGGAATGTG 585**

**Pelodiscus_HSC70_HQ219723 GCTGCTGCTATTGCTTATGGCTTGGACAAGAAGGTTGG------AGCGGAAAGGAATGTC 585**

**Bos_HSP70_U09861 GCCGCCGCCATCGCCTACGGCCTGGACAGGACGGGCAA------GGGGGAGCGCAACGTG 585**

**Human_HSPA1A_NM_005345 GCCGCCGCCATCGCCTACGGCCTGGACAGAACGGGCAA------GGGGGAGCGCAACGTG 585**

**Rattus_HSP70_1A_NM_031971 GCGGCCGCCATCGCCTACGGGCTGGACCGGACCGGCAA------GGGCGAGCGCAACGTG 585**

**X_maculatus_HSP70-1_AB062113 GCTGCAGCCATTGCCTACGGCCTTGATAAAGGCAAAAG------AGGAGAGCGCAATGTC 591**

**X_maculatus_HSP70-2_AB062114 GCGGCCGCCATCGCCTACGGTCTGGACAAAGGCAAGTC------GGGAGAGAGAAACGTC 591**

**Human_HSPA1L_NM_005527 GCTGCTGCCATTGCCTATGGTTTAGATAAAGGAGGTCA------AGGAGAACGACATGTC 591**

**Human_HSPA6_NP_002146 GCAGCTGCCATCGCCTATGGGCTGGACCGGCGGGGCGC------GGGAGAGCGCAACGTG 591**

**Alligator_HSP70_AB306279 GCGGCCGCCATCGCCTACGGGCTGGACAAGAAGGGCGCGCGCGCCGGCGAGAAGAACGTG 594**

**** ** ** * ** ** ** * ** ** * ***

**Drosophila_Bb_AF295957 CTTATCCTCGACTTGGGCGGCGGCACCTTCGATGTCTCCATCCTGACCATCGACGAGGGA 636**

**Drosophila_Aa_AF295933 CTTATCTTCGACTTGGGCGGCGGCACCTTCGATGTCTCCATCCTGACCATCGACGAGGGA 636**

**Spodoptera_FJ754276 CTCATTTTCGACCTCGGCGGCGGAACGTTCGACGTGTCGATCCTGACCATCGACGAAGGT 636**

**Bombyx_AB035326 CTTATCTTCGATCTGGGTGGAGGAACCTTCGACGTGTCCATCCTGACTATCGACGAGGGA 636**

**Paralvinella_EF580992 TTGATATTTGACCTCGGAGGAGGAACGTTCGATGTGTCGATTCTCTCGATTGCCGATGGT 651**

**B_plicatilis_HSP70-1_AB775784 CTGATCTTTGACCTGGGCGGCGGCACCTTCGACGTCTCCATCCTGACCATCGAAGAGGG- 644**

**B_plicatilis_HSP70-2_AB775785 CTGATCTTTGACCTGGGCGGCGGCACCTTCGACGTCTCCATCCTGACCATCGAAGAGGG- 644**

**A_brightwelli_HSP70_KX119429 TTAATCTTCGACTTGGGCGGCGGTACTTTCGATGTGTCGATCTTGACCATTGAAGAAGG- 641**

**Wuchereria_AF167352 CTTATCTTTGATCTTGGTGGTGGTACATTTGATGTGTCTATCCTAACTATCGAGGATGG- 644**

**Setaria_AF079360 CTTATATTTGACCTTGGTGGTGGCACATTTGATGTGTCTATCCTGACTATAGAGGATGG- 644**

**C_elegans_HSP70A_M18540 CTTATCTTCGATCTTGGAGGTGGTACCTTCGATGTCTCCATTCTTACCATTGAGGACGG- 647**

**C_elegans_HSP-1_NM_070667 CTTATCTTCGATCTTGGAGGTGGTACCTTCGATGTCTCCATTCTTACCATTGAGGACGG- 647**

**M_nipponense_KC460343 CTGATTTTTGATTTAGGTGGTGGCACTTTTGATGTATCTATTCTAACTATTGAAGATGA- 644**

**M_nipponense_DQ660140 CTGATTTTTGATTTAGGTGGTGGCACTTTTGATGTATCTATTTTAACTATTGAAGATGG- 644**

**Mytilus_HSP70_AY861684 CTCATCTTTGACTTGGGTGGTGGAACTTTTGATGTGTCAATCCTTACAATTAAGGATGG- 647**

**Perinereis_KU255783 CTGATTTTTGATCTCGGTGGTGGAACCTTCGATGTGTCCATCTTGACCATTGAGGATGG- 641**

**Paralvinella_HSP70-2_EF580993 CTGATCTTCGATCTGGGTGGAGGCACCTTTGATGTGTCCGTTCTGACCATAGAGGATGG- 644**

**Haliotis_FJ812176 CTCATCTTTGATCTTGGCGGTGGTACATTTGATGTGTCTATCTTGACAATCGAAGATGG- 644**

**Oncorhynchus_HSC71_AAB21658 CTTATCTTTGATCTGGGTGGCGGCACCTTTGACGTGTCCATCTTGACCATCGAGGATGG- 644**

**X_maculatus_HSC70_AB062115 CTCATCTTCGACCTGGGCGGCGGCACTTTCGACGTCTCCATCCTGACCATCGAGGACGG- 644**

**Human_HSPA8_HSC71_NP_006588 CTCATCTTTGACCTGGGAGGTGGCACTTTTGATGTGTCAATCCTCACTATTGAGGATGG- 644**

**Rattus_HSC71_NM_024351 CTCATTTTTGACTTGGGAGGTGGCACTTTTGATGTGTCAATCCTCACTATCGAGGATGG- 644**

**Pelodiscus_HSC70_HQ219723 CTTATCTTTGACCTTGGGGGTGGCACTTTCGATGTTTCAATCCTCACTATTGAGGATGG- 644**

**Bos_HSP70_U09861 CTCATCTTTGATCTGGGAGGGGGCACGTTCGACGTGTCCATCCTGACGATCGACGACGG- 644**

**Human_HSPA1A_NM_005345 CTCATCTTTGACCTGGGCGGGGGCACCTTCGACGTGTCCATCCTGACGATCGACGACGG- 644**

**Rattus_HSP70_1A_NM_031971 CTCATCTTCGACCTGGGGGGCGGCACGTTCGACGTGTCCATCCTGACGATCGACGACGG- 644**

**X_maculatus_HSP70-1_AB062113 CTCATCTTTGATCTTGGTGGAGGCACTTTTGACGTGTCCATCCTGACCATTGAGGATGG- 650**

**X_maculatus_HSP70-2_AB062114 CTGATCTTTGACCTGGGCGGAGGCACCTTCGACGTGTCCGTCCTGACCATCGAGGACGG- 650**

**Human_HSPA1L_NM_005527 CTGATTTTTGATCTGGGTGGAGGCACATTTGATGTGTCAATTCTGACCATAGATGATGG- 650**

**Human_HSPA6_NP_002146 CTCATTTTTGACCTGGGTGGGGGCACCTTCGATGTGTCGGTTCTCTCCATTGACGCTGG- 650**

**Alligator_HSP70_AB306279 CTGATCTTCGACCTGGGCGGCGGCACCCTCGACGTGTCCATCCTCACCATCGAGGACGG- 653**

*** ** * ** * ** ** ** ** * ** ** ** * * * ** * ***

**Drosophila_Bb_AF295957 TCTCTGTTCGAGGTGCGCTCCACAGCCGGAGACACACACTTGGGCGGCGAGGACTTTGAC 696**

**Drosophila_Aa_AF295933 TCTCTGTTCGAGGTGCGCTCCACCGCCGGAGACACGCACTTGGGCGGCGAGGACTTTGAC 696**

**Spodoptera_FJ754276 TCATTGTTCGAAGTGAGGGCGACGGCGGGCGACACGCACCTCGGAGGCGAGGACTTCGAC 696**

**Bombyx_AB035326 TCGTTGTTCGAAGTGAAGTCCACAGCCGGCGACACGCACCTGGGAGGCGAAGACTTTGAT 696**

**Paralvinella_EF580992 TCGCTGTTCGAGGTGAAGTCGACGGCCGGAGACACGCATCTCGGTGGCGAGGACTTCGAC 711**

**B_plicatilis_HSP70-1_AB775784 --CATCTTCGAGGTCAAGTCCACTGCCGGCGACACTCATTTAGGCGGCGAGGACTTCGAC 702**

**B_plicatilis_HSP70-2_AB775785 --CATCTTCGAGGTCAAGTCCACTGCCGGCGACACTCATTTAGGCGGCGAGGACTTCGAC 702**

**A_brightwelli_HSP70_KX119429 --TATCTTCGAGGTAAAATCCACGGCCGGAGACACACATTTGGGCGGCGAGGACTTTGAT 699**

**Wuchereria_AF167352 --TATTTTCGAAGTCAAATCTACAGCTGGAGATACTCATCTTGGAGGAGAAGATTTCGAT 702**

**Setaria_AF079360 --TATTTTCGAAGTGAAATCTACTGCTGGTGACACTCATCTTGGAGGCGAAGATTTCGAT 702**

**C_elegans_HSP70A_M18540 --AATCTTCGAAGTCAAGTCTACCGCTGGAGACACTCATCTTGGAGGAGAGGACTTCGAT 705**

**C_elegans_HSP-1_NM_070667 --AATCTTCGAAGTCAAGTCTACCGCTGGAGACACTCATCTTGGAGGAGAGGACTTCGAT 705**

**M_nipponense_KC460343 --CATTTTTGAAGTGAAGTCTACTGCTGGTGACACACATTTAGGTGGTGAAGATTTTGAT 702**

**M_nipponense_DQ660140 --CATTTTTGAAGTGAAGTCTACTGCTGGTGACACACATTTAGGTGGTGAAGATTTTGAT 702**

**Mytilus_HSP70_AY861684 --TATTTTTGAAGTTAAATCAACCTCTGGTGATACCCACTTGGGTGGTGAAGACTTTGAC 705**

**Perinereis_KU255783 --TATCTTTGAAGTGAAGTCAACAGCCGGAGACACCCATTTGGGAGGAGAGGATTTCGAC 699**

**Paralvinella_HSP70-2_EF580993 --CATCTTTGAAGTGAAATCCACATCCGGTGACACTCACTTGGGTGGTGAGGACTTCGAC 702**

**Haliotis_FJ812176 --TATCTTTGAGGTGAAATCTACAGCTGGAGACACCCACTTGGGTGGTGAGGACTTTGAC 702**

**Oncorhynchus_HSC71_AAB21658 --CATCTTTGAGGTCAAGTCCACTGCTGGAGACACTCATCTGGGTGGAGAAGACTTTGAC 702**

**X_maculatus_HSC70_AB062115 --CATCTTTGAGGTGAAGGCCACGGCGGGCGACACCCACCTGGGCGGCGAGGACTTCGAC 702**

**Human_HSPA8_HSC71_NP_006588 --AATCTTTGAGGTCAAGTCTACAGCTGGAGACACCCACTTGGGTGGAGAAGATTTTGAC 702**

**Rattus_HSC71_NM_024351 --AATTTTTGAAGTCAAATCAACAGCTGGAGACACCCACTTGGGCGGAGAAGACTTTGAC 702**

**Pelodiscus_HSC70_HQ219723 --CATCTTTGAAGTGAAGTCAACTGCTGGTGATACCCACTTAGGTGGGGAGGACTTTGAT 702**

**Bos_HSP70_U09861 --CATCTTCGAGGTGAAGGCCACGGCCGGGGACACGCACCTGGGCGGGGAGGACTTCGAC 702**

**Human_HSPA1A_NM_005345 --CATCTTCGAGGTGAAGGCCACGGCCGGGGACACCCACCTGGGTGGGGAGGACTTTGAC 702**

**Rattus_HSP70_1A_NM_031971 --CATCTTCGAGGTGAAGGCCACGGCGGGCGACACGCACCTGGGCGGGGAGGACTTCGAC 702**

**X_maculatus_HSP70-1_AB062113 --CATATTTGAGGTGAAATCCACGGCCGGGGACACACATCTCGGCGGAGAGGATTTTGAC 708**

**X_maculatus_HSP70-2_AB062114 --CATCTTTGAGGTGAAGGCCACGGCCGGAGACACTCACTTGGGAGGAGAGGACTTTGAC 708**

**Human_HSPA1L_NM_005527 --GATTTTTGAGGTAAAGGCCACTGCTGGGGACACTCACCTGGGTGGGGAGGACTTTGAC 708**

**Human_HSPA6_NP_002146 --TGTCTTTGAGGTGAAAGCCACTGCTGGAGATACCCACCTGGGAGGAGAGGACTTCGAC 708**

**Alligator_HSP70_AB306279 --CATCTTCGAGGTGAAGTCGACGGCGGGCGACACGCACCTGGGCGGCGAGGACTTCGAC 711**

*** ** ** ** * ** * ** ** ** ** * ** ** ** ** ** ****

**Drosophila_Bb_AF295957 AACCGGCTAGTCACCCACCTGGCGGAGGAGTTCAAGCGCAAGTACAAGAAGGATCTGCGC 756**

**Drosophila_Aa_AF295933 AACCGGCTAGTCACTCATCTGGCGGACGAGTTCAAGCGCAAGTACAAGAAGGATCTGCGC 756**

**Spodoptera_FJ754276 AACAGATTGGTGAACCATCTCGCGGACGAGTTCAAGCGCAAGTACAAGAAGGACATGCGC 756**

**Bombyx_AB035326 AACCGACTAGTCAACCATCTTGCGGAAGAGTTCAAGCGCAAGTACAAGAAGGATCTGCGC 756**

**Paralvinella_EF580992 AACAGACTGGTGAACCATTTCATCAAGGAATTCTTTAGAAAGTACAAGAAAGACATCAGC 771**

**B_plicatilis_HSP70-1_AB775784 AACCGACTGGTCAACCATTTTGTCGAGGAGTTCAAGCGCAAGAACAAAAAGGACATCACC 762**

**B_plicatilis_HSP70-2_AB775785 AACCGACTGGTCAACCATTTTGTCGAGGAGTTCAAGCGCAAGAACAAAAAGGACATCACC 762**

**A_brightwelli_HSP70_KX119429 AACAGACTCGTGAACCATTTCGTCGAAGAATTCAAGAGAAAACATAAAAAAGACATCACC 759**

**Wuchereria_AF167352 AATCGCATGGTGAATCATTTCGTTGCGGAATTCAAGCGGAACGACAAAAAGGATCTTGCG 762**

**Setaria_AF079360 AACCGCATGGTGAATCATTTCGTTGCGGAATTCAAGCGTAAACACAAGAAGGATTTGGCT 762**

**C_elegans_HSP70A_M18540 AACCGCATGGTGAACCACTTCTGTGCCGAGTTCAAGCGCAAGCACAAGAAGGATCTTGCT 765**

**C_elegans_HSP-1_NM_070667 AACCGCATGGTGAACCACTTCTGTGCCGAGTTCAAGCGCAAGCACAAGAAGGATCTTGCT 765**

**M_nipponense_KC460343 AACAGAACGGTTAACCATTTCATTCAAGAATTCAAGAGAAAATACAAGAAAGATCCTTCC 762**

**M_nipponense_DQ660140 AACAGAATGGTTAACCATTTCATTCAAGAATTCAAGAGAAAATACAAGAAAGATCCTTCC 762**

**Mytilus_HSP70_AY861684 AACAGAATGGTCAATCATTTCATTCAAGAATTCAAACGCAAGCACAAAAAAGACATTAGT 765**

**Perinereis_KU255783 AACAGAATGGTCAACCACTTCATTCAGGAATTCAAGCGCAAGTTCAAGAAAGACATCTCT 759**

**Paralvinella_HSP70-2_EF580993 AACCGTATGGTGAACCACTTCATTCAGGAATTTAAGAGAAAGTTCAAGAAGGACATCTCC 762**

**Haliotis_FJ812176 AATCGTATGGTGAACCACTTCATCCAAGAGTTCAAAAGGAAACACAAGAAGGATATCTCT 762**

**Oncorhynchus_HSC71_AAB21658 AACCGCATGGTCAACCACTTCATCGCGGAGTTCAAACGCAAGTACAAGAAAGACATCAGC 762**

**X_maculatus_HSC70_AB062115 AACCGCATGGTGAACCACTTCATCTCCGAGTTCAAGCGCAAGTACAAGAAGGACATCAGC 762**

**Human_HSPA8_HSC71_NP_006588 AACCGAATGGTCAACCATTTTATTGCTGAGTTTAAGCGCAAGCATAAGAAGGACATCAGT 762**

**Rattus_HSC71_NM_024351 AACCGAATGGTCAACCATTTCATTGCTGAGTTTAAGCGAAAGCACAAGAAGGACATCAGT 762**

**Pelodiscus_HSC70_HQ219723 AACCGTATGGTCAATCATTTCATTGCTGAATTCAAGCGTAAGCACAAGAAGGATATTACT 762**

**Bos_HSP70_U09861 AACAGGCTGGTGAACCACTTCGTGGAGGAGTTCAAGAGGAAGCACAAGAAGGACATCAGC 762**

**Human_HSPA1A_NM_005345 AACAGGCTGGTGAACCACTTCGTGGAGGAGTTCAAGAGAAAACACAAGAAGGACATCAGC 762**

**Rattus_HSP70_1A_NM_031971 AACCGGCTGGTGAGCCACTTCGTGGAGGAGTTCAAGAGGAAGCACAAGAAGGACATCAGC 762**

**X_maculatus_HSP70-1_AB062113 AACCGAATGGTCAGCCACTTTGTGGAGGAGTTTAAAAGAAAGCACAAGAAGGACATCAGC 768**

**X_maculatus_HSP70-2_AB062114 AACCGCATGGTCAACCACTTTGTGGAGGAGTTCAAGAGGAAACACAAGAAGGACATCAGC 768**

**Human_HSPA1L_NM_005527 AACAGGCTTGTGAGCCACTTCGTGGAGGAGTTCAAGAGGAAACACAAAAAGGACATCAGC 768**

**Human_HSPA6_NP_002146 AACCGGCTCGTGAACCACTTCATGGAAGAATTCCGGCGGAAGCATGGGAAGGACCTGAGC 768**

**Alligator_HSP70_AB306279 AACCGCATGGTGACGCACTTCGTGGAGGAGTTCAAGCGCAAGCACAAGCGCGACATCGGC 771**

**** * ** * ** * ** ** * ** ****

**Drosophila_Bb_AF295957 TCCAACCCTCGCGCCCTACGACGCCTCAGAACAGCAGCTGAACGGGCCAAGCGCACACTC 816**

**Drosophila_Aa_AF295933 TCCAACCCTCGCGCCCTACGACGCCTCAGAACAGCAGCTGAACGGGCCAAGCGCACACTC 816**

**Spodoptera_FJ754276 ATGAATCCTCGCGCACTGCGCCGCCTCCGCACAGCCGCCGAGCGCGCTAAGCGCACGCTG 816**

**Bombyx_AB035326 CTGAACTCTCGCGCACTCCGACGCCTCCGCACGGCCGCTGAGCGCGCCAAGAGGACACTC 816**

**Paralvinella_EF580992 AACAACCCTCGAGCCATCAGACGACTGAGAACGGCTTGTGAACGAGCCAAGAGGACATTA 831**

**B_plicatilis_HSP70-1_AB775784 TCGAACAAGCGAGCGCTGCGCCGACTCCGCACTGCTTGCGAGAGGGCCAAGCGCACTCTG 822**

**B_plicatilis_HSP70-2_AB775785 TCGAACAAGCGAGCGCTGCGCCGACTCCGCACTGCTTGCGAGAGGGCCAAGCGCACTCTG 822**

**A_brightwelli_HSP70_KX119429 GGAAACAAACGTGCCATTCGAAGATTGAGAACGGCCTGTGAAAGAGCAAAGAGAACTTTG 819**

**Wuchereria_AF167352 TCGAATCCTCGTGCGCTTCGTCGTTTACGTACTGCATGCGAAAGAGCAAAACGAACATTG 822**

**Setaria_AF079360 TCAAACCCTCGTGCCCTTCGTCGCTTACGTACTGCATGCGAAAGAGCAAAAAGAACACTC 822**

**C_elegans_HSP70A_M18540 TCCAACCCACGTGCTCTTCGTCGTCTTCGTACCGCCTGCGAGCGCGCAAACGAGACTCTT 825**

**C_elegans_HSP-1_NM_070667 TCCAACCCACGTGCTCTTCGTCGTCTTCGTACCGCCTGCGAGCGCGCAAAGAGAACTCTT 825**

**M_nipponense_KC460343 GAAAACAAGCGTGCTCTACGTCGTTTACGTACTGCTTGTGAGCGTGCCAAGCGTACTCTT 822**

**M_nipponense_DQ660140 GAAAACAAGCGTGCTCTACGTCGTTTACGTACTGCTTGTGAGCGCGCCAAGCGTACTCTT 822**

**Mytilus_HSP70_AY861684 GAAAACAAGCGTGCTGTCCGACGACTTAGAACTGCTTGTGAAAGGGCAAAGAGAACCCTT 825**

**Perinereis_KU255783 GACAACAAGAGGGCTGTAAGACGTCTCCGAACAGCCTGTGAGCGTGCGAAGAGGACCCTG 819**

**Paralvinella_HSP70-2_EF580993 GACAACAAGCGTGCTGTTCGTCGTCTGCGTACAGCATGTGAACGCGCGAAACGTACCTTG 822**

**Haliotis_FJ812176 GACAACAAGAGGGCTGTGAGACGTCTGAGAACCGCATGCGAGAGAGCAAAGAGGACACTT 822**

**Oncorhynchus_HSC71_AAB21658 GACAACAAGAGGGCTGTTCGCCGTCTCCGCACCGCATGTGAGAGGGCAAAGCGCACCCTG 822**

**X_maculatus_HSC70_AB062115 GACAACAAGAGGGCGGTGCGTCGCCTGCGCACGGCCTGCGAGCGGGCCAAGCGCACGCTG 822**

**Human_HSPA8_HSC71_NP_006588 GAGAACAAGAGAGCTGTAAGACGCCTCCGTACTGCTTGTGAACGTGCTAAGCGTACCCTC 822**

**Rattus_HSC71_NM_024351 GAGAACAAGAGAGCTGTCAGGCGTCTCCGCACTGCCTGTGAGCGGGCCAAGCGCACCCTC 822**

**Pelodiscus_HSC70_HQ219723 GAGAACAAGAGAGCAGTTCGCCGGCTACGCACAGCATGTGAACGTGCTAAGCGTACTCTC 822**

**Bos_HSP70_U09861 CAGAACAAGCGGGCCGTGAGGCGGCTGCGCACCGCATGCGAGCGGGCCAAGAGAACCTTG 822**

**Human_HSPA1A_NM_005345 CAGAACAAGCGAGCCGTGAGGCGGCTGCGCACCGCCTGCGAGAGGGCCAAGAGGACCCTG 822**

**Rattus_HSP70_1A_NM_031971 CAGAACAAGCGCGCGGTGCGGCGACTGCGCACGGCGTGCGAGAGGGCCAAGAGGACGCTG 822**

**X_maculatus_HSP70-1_AB062113 CAGAACAAGAGAGCAGTGAGGAGACTGCGCACAGCTTGTGAGAGAGCAAAGAGGACCTTG 828**

**X_maculatus_HSP70-2_AB062114 CAGAACAAGAGAGCCTTGAGGAGGCTGCGCACAGCTTGCGAGAGGGCCAAGAGGACCCTG 828**

**Human_HSPA1L_NM_005527 CAGAACAAGCGAGCCGTGAGGCGGCTGCGCACCGCCTGCGAGAGGGCCAAGAGGACCCTG 828**

**Human_HSPA6_NP_002146 GGGAACAAGCGTGCCCTGCGCAGGCTGCGCACAGCCTGTGAGCGCGCCAAGCGCACCCTG 828**

**Alligator_HSP70_AB306279 GGCAACAAGCGGGCGGTGCGGCGGCTGCGCACGGCGTGCGAGCGGGCCAAGCGCACGCTG 831**

**** * ** * * * * * ** ** ** * ** ** ** ***

**Drosophila_Bb_AF295957 TCCTCTAGCACGGAGGCCACCATCGAGATCGACGCATTGTTTGAGGGCCAAGACTTCTAC 876**

**Drosophila_Aa_AF295933 TCCTCCAGCACGGAGGCCACCATCGAGATTGACGCACTGTTTGAGGGCCAAGACTTCTAC 876**

**Spodoptera_FJ754276 TCGTCCAGCCCCGAAGCCACCATCGAGATCGACGCGCTCTACGAGGGAATCGACTTCTAC 876**

**Bombyx_AB035326 TCATCCAGCACCGAGGCCACCATCGAGATTGACGCTCTGTATGAGGGCATCGACTTCTAC 876**

**Paralvinella_EF580992 TCGACCAGTACACAATCGAATATAGAAATCGACTCTCTCTACGAAGGAATCGACTTCTAT 891**

**B_plicatilis_HSP70-1_AB775784 TCCTCGTCGGCCCAGGCCAACATCGAAATAGACTCGTTGCACGAGGGCGTCGACTTCTAC 882**

**B_plicatilis_HSP70-2_AB775785 TCCTCGTCGGCCCAGGCCAACATCGAAATAGACTCGTTGCACGAGGGCGTCGACTTCTAC 882**

**A_brightwelli_HSP70_KX119429 TCATCGTCTGCCCAGGCCAACATTGAGATTGACTCGTTGCATGAGGGAGTTGACTTTTAC 879**

**Wuchereria_AF167352 TCTAGTTCATCTCAAGCGAGTATCGAAATTGATTCGTTGTTCGAGGGGATCGATTTCTAC 882**

**Setaria_AF079360 TCTAGTTCGTCACAAGCTAGTATCGAAATTGATTCGCTATTCGAGGGCATTGATTTCTAC 882**

**C_elegans_HSP70A_M18540 TCGTCGTCTTGCCAGGCTTCGATTGAGATCGATTCTCTCTTCGAAGGAATTGACTTCTAC 885**

**C_elegans_HSP-1_NM_070667 TCGTCGTCTTCCCAGGCTTCAATTGAGATCGATTCTCTCTTCGAGGGAATTGACTTCTAC 885**

**M_nipponense_KC460343 TCAGCCTCTGCTCAAGCTAGTGTTGAGATTGATTCTCTGTATGAAGGTATTGATTTTTAC 882**

**M_nipponense_DQ660140 TCAGCCTCTGCTCAAGCTAGTGTTGAGATTGATTCTCTGTATGAAGGTATTGATTTTTAC 882**

**Mytilus_HSP70_AY861684 TCTTCAAGCACACAAGCAAGTGTTGAGATTGACTCTCTGTTTGAAGGAGTTGACTTTTAT 885**

**Perinereis_KU255783 TCCTCCAGCACCCAAGCCAGTATTGAAATTGATTCCTTGTATGAAGGTGTCGACTTCTAC 879**

**Paralvinella_HSP70-2_EF580993 TCCAGCAGCACACAGGCAAGCATTGAAATAGATTCCCTCTATGAGGGCATAGACTATTAC 882**

**Haliotis_FJ812176 TCCTCCAGCACCCAGGCCAGCATAGAGATCGACTCCCTGTTTGAAGGAGTAGATTACTAC 882**

**Oncorhynchus_HSC71_AAB21658 TCCTCCAGCACCCAGGCCAGCATCGAGATCGACTCTTTGTACGAGGGAATCGACTTCTAC 882**

**X_maculatus_HSC70_AB062115 TCTTCCAGCACGCAGGCCAGCATCGAGATCGACTCCCTGTACGAGGGCATCGACTTCTAC 882**

**Human_HSPA8_HSC71_NP_006588 TCTTCCAGCACCCAGGCCAGTATTGAGATCGATTCTCTCTATGAAGGAATCGACTTCTAT 882**

**Rattus_HSC71_NM_024351 TCCTCCAGCACCCAGGCCAGTATTGAGATTGATTCTCTCTATGAGGGAATTGACTTCTAC 882**

**Pelodiscus_HSC70_HQ219723 TCTTCTAGCACTCAGGCCAGTATTGAAATTGACTCGCTCTATGAGGGCATTGATTTTTAC 882**

**Bos_HSP70_U09861 TCGTCCAGCACCCAGGCCAGCCTGGAGATCGACTCCCTGTTCGAGGGCATCGACTTCTAC 882**

**Human_HSPA1A_NM_005345 TCGTCCAGCACCCAGGCCAGCCTGGAGATCGACTCCCTGTTTGAGGGCATCGACTTCTAC 882**

**Rattus_HSP70_1A_NM_031971 TCGTCCAGCACCCAGGCCAGCCTGGAGATCGACTCTCTGTTCGAGGGCATCGACTTCTAC 882**

**X_maculatus_HSP70-1_AB062113 TCCTCCAGCACTCAGGCAAGCATTGAGATTGACTCACTCTTTGAGGGCATTGACTTTTAC 888**

**X_maculatus_HSP70-2_AB062114 TCCTCCAGCTCCCAGGCCAGCATCGAGATCGACTCTCTGTTCGAGGGTGTGGACCTGTAC 888**

**Human_HSPA1L_NM_005527 TCGTCCAGCACCCAGGCCAACCTAGAAATTGATTCACTTTATGAAGGCATTGACTTCTAT 888**

**Human_HSPA6_NP_002146 TCCTCCAGCACCCAGGCCACCCTGGAGATAGACTCCCTGTTCGAGGGCGTGGACTTCTAC 888**

**Alligator_HSP70_AB306279 AGCTCCTCCACGCAGGCCAGCATCGAGATCGACTCGCTGTTCGAGGGCATCGACTTCTAC 891**

*** * * ** ** ** * * ** ** ** ****

**Drosophila_Bb_AF295957 ACCAAAGTAAGCCGTGCCAGGTTTGAGGAGCTGTGCGCGGACCTCTTCCGCAACACCCTG 936**

**Drosophila_Aa_AF295933 ACCAAAGTGAGCCGCGCCAGGTTTGAGGAGCTGTGCGCGGACCTCTTCCGCAACACCCTG 936**

**Spodoptera_FJ754276 ACTCGCGTGTCCCGCGCGCGCTTCGAGGAGCTCAACGCTGACCTGTTCCGAGGCACCCTC 936**

**Bombyx_AB035326 ACGCGAGTCTCCCGCGCCCGCTTCGAGGAACTGAACGCGGACCTGTTCAGGGGAACTCTG 936**

**Paralvinella_EF580992 ACAACTATTACCAGAGCCAGATTCGAAGAGCTCTGCTCCGATCTGTTCAGGTCAACTTTG 951**

**B_plicatilis_HSP70-1_AB775784 ACGAGCATCACCAGAGCTCGCTTTGAAGAGCTGTGCGCCGATTTGTTCCGCGGCACTCTC 942**

**B_plicatilis_HSP70-2_AB775785 ACGAGCATCACCAGAGCTCGCTTTGAAGAGCTGTGCGCCGATTTGTTCCGCGGCACCCTC 942**

**A_brightwelli_HSP70_KX119429 ACAAACATCACCAGAGCCAGATTCGAAGAGCTCAACGCTGATTTATTCCGCGGCTGTTTA 939**

**Wuchereria_AF167352 ACCAATATCACTCGTGCTCGCTTTGAGGAATTATGTGCTGATCTTTTCCGTTCAACTATG 942**

**Setaria_AF079360 ACTAATATTACTCGTGCTCGCTTTGAAGAACTTTGTGCTGATCTTTTCCGCTCAACAATG 942**

**C_elegans_HSP70A_M18540 ACCAACATCACTCGTGCTCGTTTCGAGGAGCTCTGCGCTGATCTCTTCAGATCCACCATG 945**

**C_elegans_HSP-1_NM_070667 ACCAACATCACTCGTGCTCGTTTCGAGGAGCTCTGCGCTGATCTCTTCAGATCCACCATG 945**

**M_nipponense_KC460343 ACTTCTATTACTCGTGCCAGGTTTGAGGAACTTTGTGGTGACTTGTTCCGTGGCACCCTT 942**

**M_nipponense_DQ660140 ACTTCTATTACTCGTGCCAGGTTTGAGGAACTTTGTGGTGACTTGTTCCGTGGCACCCTG 942**

**Mytilus_HSP70_AY861684 ACAAGCATCACAAGAGCCAGGTTTGAGGAATTGAATGCAGATCTTTTCAGAGGAACCATG 945**

**Perinereis_KU255783 ACAACCATCACCAGGGCTCGTTTTGAGGAGCTGTGTGCCGATCTCTTCCGAGGCACTCTC 939**

**Paralvinella_HSP70-2_EF580993 ACAACAATCACTAGAGCTAGATTTGAGGAGCTGAATGCTGACCTATTCCGTGGTACGCTG 942**

**Haliotis_FJ812176 ACCAGCATCACTAGAGCCAGGTTTGAGGAGCTCAATGCTGATCTTTTCCGTGGCACACTA 942**

**Oncorhynchus_HSC71_AAB21658 ACCTCCATCACCAGGGCTCGCTTTGAGGAGCTCAATGCAGACCTTTTCCGTGGCACCCTT 942**

**X_maculatus_HSC70_AB062115 ACGTCCATCACCAGGGCGCGCTTTGAGGAGCTGAACGCGGACCTGTTCCGCGGAACGCTG 942**

**Human_HSPA8_HSC71_NP_006588 ACCTCCATTACCCGTGCCCGATTTGAAGAACTGAATGCTGACCTGTTCCGTGGCACCCTG 942**

**Rattus_HSC71_NM_024351 ACCTCCATTACCCGTGCTCGATTTGAGGAGTTGAATGCTGACCTGTTCCGTGGCACACTG 942**

**Pelodiscus_HSC70_HQ219723 ACCTCAATTACCAGAGCTCGTTTTGAAGAGTTAAATGCTGATCTGTTCCGTGGCACTCTG 942**

**Bos_HSP70_U09861 ACGTCCATCACCAGGGCGCGGTTCGAGGAGCTGTGCTCCGACCTGTTCCGGAGCACCCTA 942**

**Human_HSPA1A_NM_005345 ACGTCCATCACCAGGGCGAGGTTCGAGGAGCTGTGCTCCGACCTGTTCCGAAGCACCCTG 942**

**Rattus_HSP70_1A_NM_031971 ACGTCCATCACGCGGGCGCGGTTCGAGGAGCTGTGCTCGGACCTGTTCCGCGGCACGCTG 942**

**X_maculatus_HSP70-1_AB062113 ACCTCAATCACAAGGGCACGCTTCGAGGAGCTCAACTCAGACCTCTTTAGGGGAACACTG 948**

**X_maculatus_HSP70-2_AB062114 ACCTCCATCACCAGGGCTCGCTTTGAGGAGCTGTGCTCCGACCTGTTCAGGGGAACCTTG 948**

**Human_HSPA1L_NM_005527 ACATCCATCACCAGAGCTCGATTTGAAGAGTTGTGTGCAGACCTGTTTAGGGGTACCCTG 948**

**Human_HSPA6_NP_002146 ACGTCCATCACTCGTGCCCGCTTTGAGGAACTGTGCTCAGACCTCTTCCGCAGCACCCTG 948**

**Alligator_HSP70_AB306279 ACGTCCATCACGCGGGCGCGCTTCGAGGAGCTGAACGCCGACCTGTTCCGCGGCACGCTG 951**

**** * * ** * ** ** ** * ** * ** * ***

**Drosophila_Bb_AF295957 CAGCCTGTGGAGAAGGCCCTCAACGATGCCAAGATGGACAAGGGTCAGATCCACGACATC 996**

**Drosophila_Aa_AF295933 CAGCCTGTGGAGAAGGCCCTCAACGATGCCAAGATGGATAAGGGTCAGATCCACGACATC 996**

**Spodoptera_FJ754276 GAGCCGGTCGAGAAAGCGCTCAAGGACGCCAAGATGGACAAGAGCCAGATCCACGACGTC 996**

**Bombyx_AB035326 GAACCCGTCGAGAAGGCACTCAAGGATGCTAAACTCGACAAGAGTCAGATCCACGACGTG 996**

**Paralvinella_EF580992 GAGCCAGTAGAGAAAGCTCTCCGAGATGCCAAGATGGACAAGTCCAAGATCGACGAAGTG 1011**

**B_plicatilis_HSP70-1_AB775784 GACCCAGTGGAGAAGGCGCTGCGAGACGCCAAAATGGACAAGTCAAGTGTGAACGAGATT 1002**

**B_plicatilis_HSP70-2_AB775785 GACCCAGTGGAGAAGGCGCTGCGAGACGCCAAAATGGACAAGTCAAGTGTGAACGAGATT 1002**

**A_brightwelli_HSP70_KX119429 GACCCAGTCGAGAAATCTTTGAGAGACGCTAAACTCGACAAAGCCCAGATCCATGAAATT 999**

**Wuchereria_AF167352 GATCCAGTTGAGAAAGCACTCCGTGATGCTAAGATGGACAAGGCCCAAGTCCATGACATT 1002**

**Setaria_AF079360 GATCCAGTTGAGAAAGCATTGCGTGATGCCAAGATGGATAAGGCACAAGTTCATGATATT 1002**

**C_elegans_HSP70A_M18540 GACCCAGTCGAGAAGTCTCTCCGTGACGCCAAGATGGACAAGAGCCAAGTTCATGACATC 1005**

**C_elegans_HSP-1_NM_070667 GACCCAGTCGAGAAGTCTCTCCGTGACGCCAAGATGGACAAGAGCCAAGTTCATGACATC 1005**

**M_nipponense_KC460343 GAGCCAGTTGAGAAGTCTCTGAGAGATGCAAAAATGGACAAAGCCCAAATCTATGACATT 1002**

**M_nipponense_DQ660140 GAGCCAGTTGAGAAGTCTCTGAGAGATGCAAAAATGGACAAAGCCCAAATCCATGACATT 1002**

**Mytilus_HSP70_AY861684 GAACCAGTTGAAAAAGCTCTACGCGATGCCAAACTAGACAAGGCTGCTGTCCATGAAATT 1005**

**Perinereis_KU255783 GAGCCAGTAGAGAAGTCCATCCGTGATGCCAAGATGGGAAAAGATGCCATCCACGACATC 999**

**Paralvinella_HSP70-2_EF580993 GAACCTGTAGAAAAGTCTCTGCGTGATGCCAAGCTGGACAAGTCCAGCATCCATGACATC 1002**

**Haliotis_FJ812176 GAGCCAGTGGAGAAGGCTTTACGTGATGCCAAGGCAGACAAGGTCTCCATCCATGACATA 1002**

**Oncorhynchus_HSC71_AAB21658 GACCCAGTGGAGAAATCCCTCCGCGACGCCAAGATGGACAAAGCCCAGGTACACGACATC 1002**

**X_maculatus_HSC70_AB062115 GAGCCCGTGGAGAAGTCTCTCCGAGACGCCAAGATGGACAAGGCTCAGATCCACGAGATT 1002**

**Human_HSPA8_HSC71_NP_006588 GACCCAGTAGAGAAAGCCCTTCGAGATGCCAAACTAGACAAGTCACAGATTCATGATATT 1002**

**Rattus_HSC71_NM_024351 GACCCTGTAGAGAAGGCCCTTCGAGATGCCAAACTAGACAAGTCACAGATCCATGATATT 1002**

**Pelodiscus_HSC70_HQ219723 GATCCTGTGGAGAAGGCCCTGCGGGATGCCAAACTAGACAAATCACAGATCCATGACATT 1002**

**Bos_HSP70_U09861 GAGCCCGTGGAGAAGGCGCTACGCGACGCCAAGCTGGACAAGGCGCAGATCCACGACCTG 1002**

**Human_HSPA1A_NM_005345 GAGCCCGTGGAGAAGGCTCTGCGCGACGCCAAGCTGGACAAGGCCCAGATTCACGACCTG 1002**

**Rattus_HSP70_1A_NM_031971 GAGCCCGTGGAGAAGGCCCTGCGCGACGCCAAGCTGGACAAGGCGCAGATCCACGACCTG 1002**

**X_maculatus_HSP70-1_AB062113 GAGCCAGTTGAGAAGGCCCTACAAGATGCTAAGCTGGATAAATCCAAGATCCACGAAGTT 1008**

**X_maculatus_HSP70-2_AB062114 GAGCCTGTGGAGAAGGCTTTGAGGGACGCCAAGATGGACAAGGGCCAGATCCATGACGCG 1008**

**Human_HSPA1L_NM_005527 GAGCCTGTAGAAAAAGCGCTTCGGGATGCCAAGATGGATAAGGCTAAAATCCATGACATT 1008**

**Human_HSPA6_NP_002146 GAGCCGGTGGAGAAGGCCCTGCGGGATGCCAAGCTGGACAAGGCCCAGATTCATGACGTC 1008**

**Alligator_HSP70_AB306279 GAGCCCGTGGAGAAGGCGCTGCGCGACGCCAAGCTGGACAAGGGGCAGGTGCAGGAGATC 1011**

*** ** ** ** ** * * ** ** ** * ** * * ****

**Drosophila_Bb_AF295957 GTGCTCGTCGGCGGATCCACTCGCATTCCCAAGGTGCAAAGTCTGCTGCAGGAGTTCTTC 1056**

**Drosophila_Aa_AF295933 GTGCTCGTCGGCGGATCCACTCGCATTCCCAAGGTGCAAAGTCTGCTGCAGGACTTCTTC 1056**

**Spodoptera_FJ754276 GTTCTCGTCGGAGGCTCGACCAGAATCCCGAAGGTGCAGAGTCTGCTGCAAAACTTCTTC 1056**

**Bombyx_AB035326 GTCCTCGTCGGAGGCTCCACCCGCATTCCGAAAGTACAAACTATGCTCCAAAACTTCTTC 1056**

**Paralvinella_EF580992 GTTCTGGTCGGAGGATCGACTAGAATCCCGAAGATCCAGAAACTGCTACAGGAGTTCATG 1071**

**B_plicatilis_HSP70-1_AB775784 GTGTTGGTGGGCGGCTCGACCCGCATTCCAAAAGTTCAAAAGCTGCTCCAGGACTTTTTC 1062**

**B_plicatilis_HSP70-2_AB775785 GTGTTGGTGGGCGGCTCGACCCGCATTCCAAAAGTTCAAAAGCTGCTCCAGGACTTTTTC 1062**

**A_brightwelli_HSP70_KX119429 GTTTTAGTCGGTGGATCGACCAGAATCCCGAAAGTGCAAAAATTATTACAAGACTTTTTC 1059**

**Wuchereria_AF167352 GTGCTAGTAGGTGGATCAACCCGTATCCCGAAAGTGCAGAAGCTCTTGTCAGATTTCTTT 1062**

**Setaria_AF079360 GTACTGGTGGGTGGGTCAACTCGTATCCCGAAAGTGCAGAAACTGCTCTCGGATTTCTTC 1062**

**C_elegans_HSP70A_M18540 GTCCTTGTCGGAGGATCCACTCGTATCCCAAAGGTCCAGAAACTTTTGTCCGATCTCTTC 1065**

**C_elegans_HSP-1_NM_070667 GTCCTTGTCGGAGGATCCACTCGTATCCCAAAGGTCCAGAAACTTTTGTCCGATCTCTTC 1065**

**M_nipponense_KC460343 GTCCTTGTTGGAGGTTCCACCAGAATTCCAAAGATTCAGAAGCTGTTACAGGATTTCTTC 1062**

**M_nipponense_DQ660140 GTCCTTGTTGGAGGTTCCACCAGAATTCCAAAGATTCAGAAGCTGTTACAGGATTTCTTC 1062**

**Mytilus_HSP70_AY861684 GTCTTGGTAGGTGGATCAACCAGAATTCCAAAAATCCAGAAGTTACTTCAGGACTTTTTT 1065**

**Perinereis_KU255783 GTGTTGGTCGGTGGTTCCACCCGTATCCCCAAGATCCAGAAACTCCTTCAGGATTTCTTC 1059**

**Paralvinella_HSP70-2_EF580993 GTACTGGTTGGTGGCTCCACCAGAATTCCCAAGATCCAAAAACTGCTGCAGGACTTTTTC 1062**

**Haliotis_FJ812176 GTGCTGGTTGGGGGATCTACACGTATCCCCAAGATCCAGAAACTCCTCCAAGACTTCTTC 1062**

**Oncorhynchus_HSC71_AAB21658 GTCCTGGTCGGAGGCTCCACTCGTATCCCCAAGATCCAGAAACTGCTCCAAGATTTCTTC 1062**

**X_maculatus_HSC70_AB062115 GTCTTGGTGGGAGGCTCGACCCGCATCCCAAAGATCCAAAAGCTCCTGCAGGACTTCTTT 1062**

**Human_HSPA8_HSC71_NP_006588 GTCCTGGTTGGTGGTTCTACTCGTATCCCCAAGATTCAGAAGCTTCTCCAAGACTTCTTC 1062**

**Rattus_HSC71_NM_024351 GTCCTGGTGGGTGGTTCTACCAGAATCCCCAAGATCCAGAAACTTCTGCAAGACTTCTTC 1062**

**Pelodiscus_HSC70_HQ219723 GTACTGGTTGGTGGATCAACCCGTATTCCCAAGATTCAGAAACTCCTGCAGGATTTCTTC 1062**

**Bos_HSP70_U09861 GTCCTGGTGGGGGGCTCCACCCGCATCCCCAAGGTGCAGAAGCTGCTGCAGGACTTCTTC 1062**

**Human_HSPA1A_NM_005345 GTCCTGGTCGGGGGCTCCACCCGCATCCCCAAGGTGCAGAAGCTGCTGCAGGACTTCTTC 1062**

**Rattus_HSP70_1A_NM_031971 GTGCTGGTGGGCGGCTCGACGCGCATCCCCAAGGTGCAGAAGCTGCTGCAGGACTTCTTC 1062**

**X_maculatus_HSP70-1_AB062113 GTCCTAGTTGGTGGCTCTACAAGAATCCCCAAAATCCAAAAGCTCCTGCAGGACTTCTTC 1068**

**X_maculatus_HSP70-2_AB062114 GTCCTGGTGGGAGGCTCCACCCGAATCCCCAAGATCCAGAAGCTGCTGCAGGACTTCTTC 1068**

**Human_HSPA1L_NM_005527 GTTTTAGTAGGGGGCTCCACCCGCATCCCCAAGGTGCAGCGGCTGCTTCAGGACTACTTC 1068**

**Human_HSPA6_NP_002146 GTCCTGGTGGGGGGCTCCACACGCATCCCCAAGGTGCAGAAGTTGCTGCAGGACTTCTTC 1068**

**Alligator_HSP70_AB306279 GTGCTGGTGGGCGGCTCGACGCGCATCCCCAAGATCCAGAAGCTGCTGCAGGACTTCTTC 1071**

**** * ** ** ** ** ** * ** ** ** * ** * * * ***

**Drosophila_Bb_AF295957 CACGGCAAGAACCTCAACCTATCCATCAACCCAGACGAGGCAGTGGCATACGGAGCTGCT 1116**

**Drosophila_Aa_AF295933 CACGGCAAGAACCTCAACCTATCCATCAACCCAGACGAGGCAGTGGCATACGGAGCTGCT 1116**

**Spodoptera_FJ754276 TGCGGCAAGAAGCTGAATTTGTCCATCAACCCGGACGAGGCGGTCGCGTATGGCGCGGCC 1116**

**Bombyx_AB035326 TGTGGGAAGAAACTGAACTTATCCATCAATCCGGACGAAGCCGTAGCCTACGGTGCAGCA 1116**

**Paralvinella_EF580992 AACGGGAAAGAGCTGAACAAGTCGATCAATCCCGATGAAGCAGTGGCCTATGGAGCGGCC 1131**

**B_plicatilis_HSP70-1_AB775784 AATGGCAAAGAGCTGAACAAGTCGATTAACCCGGACGAGGCGGTGGCGTACGGCGCGGCC 1122**

**B_plicatilis_HSP70-2_AB775785 AATGGCAAAGAGCTGAACAAGTCGATTAACCCGGACGAGGCGGTGGCGTACGGCGCGGCC 1122**

**A_brightwelli_HSP70_KX119429 AATGGAAAAGAGTTGAACAAGAGCATTAACCCAGACGAGGCCGTGGCCTACGGTGCTGCC 1119**

**Wuchereria_AF167352 TCTGGCAAAGAATTGAATAAAAGCATCAATCCGGACGAGGCCGTGGCGTATGGTGCAGCA 1122**

**Setaria_AF079360 TCTGGCAAAGAATTGAACAAAAGTATCAACCCTGATGAGGCTGTGGCATATGGTGCTGCT 1122**

**C_elegans_HSP70A_M18540 TCAGGAAAGGAATTGAACAAGTCCATCAACCCAGATGAGGCGTTAGCCTACGGAGCTGCC 1125**

**C_elegans_HSP-1_NM_070667 TCAGGAAAGGAATTGAACAAGTCCATCAACCCAGATGAGGCCGTTGCCTACGGAGCTGCC 1125**

**M_nipponense_KC460343 AATGGAAAGGAGCTCAACAAATCCATCAATCCTGATGAAGCTGTAGCTTATGGTGCAGCT 1122**

**M_nipponense_DQ660140 AATGGAAAGGAGCTCAACAAATCCATCAATCCTGATGAAGCTGTAGCTTATGGTGCAGCT 1122**

**Mytilus_HSP70_AY861684 CAAGGCAAAGAATTGAACAAATCCATTAACCCTGATGAAGCTGTAGCATACGGTGCAGCT 1125**

**Perinereis_KU255783 AACGGCAAAGAATTGAACAAATCTATCAACCCTGATGAGGCTGTCGCATACGGAGCTGCC 1119**

**Paralvinella_HSP70-2_EF580993 AACGGCAAAGAACTAAACAAGTCGATCAACCCTGATGAAGCTGTTGCATATGGTGCAGCT 1122**

**Haliotis_FJ812176 AACGGCAAGGAGCTATGCAAGAGCATCAACCCTGATGAGGCTGTTGCTTATGGTGCTGCC 1122**

**Oncorhynchus_HSC71_AAB21658 AACGGCAAAGAGCTCAACAAAAGCATCAACCCCGACGAAGCTGTGGCCTATGGCGCAGCT 1122**

**X_maculatus_HSC70_AB062115 AACGGGAAGGAACTCAACAAGAGCATTAACCCTGACGAGGCGGTGGCGTACGGTGCAGCT 1122**

**Human_HSPA8_HSC71_NP_006588 AATGGAAAAGAACTGAATAAGAGCATCAACCCTGATGAAGCTGTTGCTTATGGTGCAGCT 1122**

**Rattus_HSC71_NM_024351 AATGGAAAAGAGCTGAATAAGAGCATTAACCCCGATGAAGCTGTTGCCTATGGTGCAGCT 1122**

**Pelodiscus_HSC70_HQ219723 AATGGGAAAGAGCTGAACAAGAGCATCAATCCTGATGAAGCTGTGGCTTACGGTGCAGCT 1122**

**Bos_HSP70_U09861 AACGGGCGCGACCTCAACAAGAGCATCAACCCCGACGAGGCGGTGGCGTACGGGGCGGCG 1122**

**Human_HSPA1A_NM_005345 AACGGGCGCGACCTGAACAAGAGCATCAACCCCGACGAGGCTGTGGCCTACGGGGCGGCG 1122**

**Rattus_HSP70_1A_NM_031971 AACGGGCGCGACCTGAACAAGAGCATCAATCCGGACGAGGCGGTGGCCTACGGGGCGGCG 1122**

**X_maculatus_HSP70-1_AB062113 AATGGTAGAGAACTAAACAAGAGCATCAACCCTGATGAAGCGGTGGCCTATGGTGCAGCA 1128**

**X_maculatus_HSP70-2_AB062114 AACGGCCGGGAGCTGAACAAGAGCATCAACCCGGACGAGGCGGTGGCTTACGGCGCCGCC 1128**

**Human_HSPA1L_NM_005527 AATGGACGTGATCTCAACAAGAGCATCAACCCTGATGAGGCCGTAGCATATGGGGCTGCG 1128**

**Human_HSPA6_NP_002146 AACGGCAAGGAGCTGAACAAGAGCATCAACCCTGATGAGGCTGTGGCCTATGGGGCTGCT 1128**

**Alligator_HSP70_AB306279 AATGGCAAGGAGCTGAACAAGAGCATCAACCCCGACGAGGCGGTGGCGTACGGCGCGGCG 1131**

**** * * ** ** ** ** ** ** * ** ** ** ** ****

**Drosophila_Bb_AF295957 GTGCAGGCCGCTATCCTCAGCGGAGACCAGAGCGGCAAGATCCAGGACGTGCTGCTGGTG 1176**

**Drosophila_Aa_AF295933 GTGCAGGCCGCTATCCTCAGCGGAGACCAGAGCGGCAAGATCCAGGACGTGCTGCTGGTG 1176**

**Spodoptera_FJ754276 GTCCAGGCGGCCATCCTGAGTGGCGAACAAGACTCGAAGATCCAGGACGTGTTGCTGGTG 1176**

**Bombyx_AB035326 GTCCAGGCAGCCATCCTGAGCGGCGAGACCGACTCCAAGATACAGGACGTCTTGTTGGTC 1176**

**Paralvinella_EF580992 GTTCAAGCAGCTATCCTGACAGGTTCTGAACACTCGGCCGTTAAAGATCTACTCTTGGTA 1191**

**B_plicatilis_HSP70-1_AB775784 GTCCAGGCGGCCATTTTGACCGGCGACAAGTCCGAGGCCGTCCAGGACCTGCTGCTGCTT 1182**

**B_plicatilis_HSP70-2_AB775785 GTCCAGGCGGCCATTTTGACCGGCGACAAGTCCGAGGCCGTCCAGGACCTGCTGCTGCTT 1182**

**A_brightwelli_HSP70_KX119429 GTCCAAGCCGCTATTTTAACGGGTGATAAATCCGAAGCCGTTCAAGATTTATTGTTATTA 1179**

**Wuchereria_AF167352 GTGCAGGCAGCTATTCTTTCTGGTGATAAGTCGGAAGCCGTACAAGATTTGTTACTGCTT 1182**

**Setaria_AF079360 GTCCAGGCAGCTATCCTCTCTGGTGATAAGTCAGAAGCCGTACAAGATTTGTTATTCGTC 1182**

**C_elegans_HSP70A_M18540 GTCCAAGCCGCTATCCTCTCCGGAGACAAGTCTGAGGCTGTCCAGGATCTTCTTCTTCTT 1185**

**C_elegans_HSP-1_NM_070667 GTCCAAGCCGCTATCCTCTCCGGAGACAAGTCTGAGGCTGTCCAGGATCTTCTTCTTCTT 1185**

**M_nipponense_KC460343 GTACAGGCAGCCATATTGTGTGGTGACAAGTCTGAAGCAGTTCAAGATTTGTTGCTTTTG 1182**

**M_nipponense_DQ660140 GTACAGGCAGCCATATTGTGTGGTGACAAGTCTGAAGCAGTTCAAGATTTGTTGCTTTTG 1182**

**Mytilus_HSP70_AY861684 GTGCAGGCAGCCATTTTGTCAGGTGACAAGTCAGAAGAGGTACAAGACTTGTTATTGTTA 1185**

**Perinereis_KU255783 GTTCAGGCTGCCATCTTGCACGGTGACAAGTCAGAAGAGGTCCAAGACTTGCTTTTGCTT 1179**

**Paralvinella_HSP70-2_EF580993 GTCCAGGCTGCCATCCTTCATGGAGACAAGTCCGAGGCAGTTCAGGATCTGCTGCTACTG 1182**

**Haliotis_FJ812176 GTCCAGGCTGCTATCCTCCATGGTGACAAGTCCGAGGAAGTCCAAGACCTGCTACTGCTG 1182**

**Oncorhynchus_HSC71_AAB21658 GTCCAGGCAGCCATACTCTCAGGTGACAAGTCTGAGAATGTCCAGGACCTGCTTTTGCTG 1182**

**X_maculatus_HSC70_AB062115 GTCCAGGCGGCCATCTTGTGCGGAGACAAATCCGAGATTGTTCAGGACCTGCTTCTGTTG 1182**

**Human_HSPA8_HSC71_NP_006588 GTCCAGGCAGCCATCTTGTCTGGAGACAAGTCTGAGAATGTTCAAGATTTGCTGCTCTTG 1182**

**Rattus_HSC71_NM_024351 GTCCAGGCAGCCATTCTATCTGGAGACAAGTCTGAGAATGTTCAGGATTTGCTGCTCTTG 1182**

**Pelodiscus_HSC70_HQ219723 GTTCAGGCAGCAATCTTGTCTGGGGACAAGTCTGAGAATGTACAAGACCTGCTACTGTTG 1182**

**Bos_HSP70_U09861 GTGCAGGCGGCCATCCTGATGGGGGACAAGTCGGAGAACGTGCAGGACCTGCTGTTGCTG 1182**

**Human_HSPA1A_NM_005345 GTGCAGGCGGCCATCCTGATGGGGGACAAGTCCGAGAACGTGCAGGACCTGCTGCTGCTG 1182**

**Rattus_HSP70_1A_NM_031971 GTGCAGGCGGCCATCCTGATGGGGGACAAGTCGGAGAACGTGCAGGACCTGCTGCTGCTG 1182**

**X_maculatus_HSP70-1_AB062113 GTCCAGGCTGCTATCCTCATGGGTGACACCTCAGAAAATGTCCAAGATTTACTGCTCCTG 1188**

**X_maculatus_HSP70-2_AB062114 GTCCAGGCCGCCATCCTGACGGGAGACACGTCGGGGAACGTCCAGGACCTGCTGCTGCTG 1188**

**Human_HSPA1L_NM_005527 GTACAAGCAGCCATCCTGATGGGGGACAAGTCTGAGAAGGTACAGGACCTGCTGCTGCTG 1188**

**Human_HSPA6_NP_002146 GTGCAGGCGGCCGTGTTGATGGGGGACAAATGTGAGAAAGTGCAGGATCTCCTGCTGCTG 1188**

**Alligator_HSP70_AB306279 GTGCAGGCCGCCATCCTCATGGGCGACAAGTCGGAGAACGTGCAGGACCTGCTGCTGCTG 1191**

**** ** ** ** * * ** * * ** * * * ***

**Drosophila_Bb_AF295957 GACGTGGCCCCACTTTCATTGGGAATTGAGACCGCTGGAGGTGTAATGACCAAGCTGATC 1236**

**Drosophila_Aa_AF295933 GACGTGGCCCCACTTTCATTGGGAATTGAGACCGCTGGAGGTGTAATGACCAAGCTGATC 1236**

**Spodoptera_FJ754276 GACGTGGCCCCTCTGTCCCTCGGCATCGAAACCGCCGGCGGCGTGATGACGAAGATCATC 1236**

**Bombyx_AB035326 GACGTGGCTCCTCTGTCCCTCGGTATTGAGACAGCCGGCGGCGTGATGACGAAGATCATC 1236**

**Paralvinella_EF580992 GACGTGACGCCATTATCTTTAGGTCTGGAGACGGCTGGAGGCGTGATGACGAAACTGGTG 1251**

**B_plicatilis_HSP70-1_AB775784 GACGTGGCGCCTCTATCGCTGGGCATTGAGACGGCCGGCGGCGTCATGACCGCCCTCATC 1242**

**B_plicatilis_HSP70-2_AB775785 GACGTGGCGCCTCTATCGCTGGGCATTGAGACCGCCGGCGGCGTCATGACCGCCCTCATC 1242**

**A_brightwelli_HSP70_KX119429 GACGTTGCTCCTTTGTCTCTTGGTATCGAGACTGCCGGCGGTGTTATGACATCATTGATC 1239**

**Wuchereria_AF167352 GACGTCGCACCGCTTTCACTTGGTATTGTAACAGCTGGGGGTGTTATGACTGCGCTTATC 1242**

**Setaria_AF079360 GACGTCGCACCGCTTTCACTTGGTATCGAGACTGCTGGTGGTGTTATGACTGCTCTCATT 1242**

**C_elegans_HSP70A_M18540 GACGTTGCCCCACTTTCCCTTGGTATTGAGACCGCTGGAGGAGTCATGACTGCTCTCATC 1245**

**C_elegans_HSP-1_NM_070667 GACGTTGCCCCACTTTCCCTTGGTATTGAGACCGCTGGAGGAGTCATGACTGCTCTCATC 1245**

**M_nipponense_KC460343 GATGTAACACCACTGTCTCTTGGTATTGAAACTGCTGGTGGTGTCATGACTGCCCTTATA 1242**

**M_nipponense_DQ660140 GATGTAACACCTTTGTCTCTTGGTATTGAAACTGCTGGAGGTGTTATGACAGCACTTATT 1242**

**Mytilus_HSP70_AY861684 GATGTTACCCCACTGTCTCTTGGTATTGAAACAGCTGGAGGTGTGATGACAGCTCTAATC 1245**

**Perinereis_KU255783 GATGTGACCCCCTTGTCCCTTGGTATCGAGACCGCGGGTGGCGTCATGACCTCCTTGATC 1239**

**Paralvinella_HSP70-2_EF580993 GATGTGACACCTCTGTCTCTGGGTATAGAGACAGCCGGTGGTGTGATGACGTCGCTGATC 1242**

**Haliotis_FJ812176 GATGTCACTCCACTGTCACTTGGTATCGAGACTGCTGGCGGTGTGATGACTGTCCTCATC 1242**

**Oncorhynchus_HSC71_AAB21658 GACGTCACACCCCTCTCCCTGGGTATTGAGACCGCTGGAGGTGTCATGACCGTCCTGATC 1242**

**X_maculatus_HSC70_AB062115 GACGTCACGCCCTTGTCTCTGGGCATCGAGACGGCAGGCGGAGTCATGACCGTGCTGATC 1242**

**Human_HSPA8_HSC71_NP_006588 GATGTCACTCCTCTTTCCCTTGGTATTGAAACTGCTGGTGGAGTCATGACTGTCCTCATC 1242**

**Rattus_HSC71_NM_024351 GATGTCACTCCTCTTTCCCTTGGGATTGAAACTGCTGGTGGAGTCATGACTGTCCTCATC 1242**

**Pelodiscus_HSC70_HQ219723 GATGTCACTCCTCTGTCTCTGGGTATTGAAACAGCAGGTGGTGTCATGACAGTCCTGATC 1242**

**Bos_HSP70_U09861 GACGTGGCTCCCCTGTCGCTGGGACTGGAGACGGCCGGAGGCGTGATGACCGCCCTGATC 1242**

**Human_HSPA1A_NM_005345 GACGTGGCTCCCCTGTCGCTGGGGCTGGAGACGGCCGGAGGCGTGATGACTGCCCTGATC 1242**

**Rattus_HSP70_1A_NM_031971 GACGTGGCGCCGCTGTCGCTGGGTCTGGAGACCGCGGGCGGCGTGATGACGGCGCTCATC 1242**

**X_maculatus_HSP70-1_AB062113 GATGTGGCACCCTTGTCGCTGGGCATTGAGACAGCCGGTGGCGTTATGACACCTCTTATC 1248**

**X_maculatus_HSP70-2_AB062114 GACGTGGCTCCTCTGTCCCTGGGGATCGAGACGGCCGGAGGAGTGATGACTGCCCTGATC 1248**

**Human_HSPA1L_NM_005527 GACGTGGCTCCCCTGTCCCTGGGGCTGGAGACGGCTGGGGGCGTGATGACTGCCCTGATA 1248**

**Human_HSPA6_NP_002146 GATGTGGCTCCCCTGTCTCTGGGGCTGGAGACAGCAGGTGGGGTGATGACCACGCTGATC 1248**

**Alligator_HSP70_AB306279 GACGTGACGCCGCTGTCGCTCGGCATCGAGACGGCGGGCGGCGTCATGACGGCGCTCATC 1251**

**** ** * ** * ** * ** * * ** ** ** ** ** ***** * ***

**Drosophila_Bb_AF295957 GAGCGCAACTGTCGCATTCCGTGCAAGCAGACTAAGACGTTCTCCACGTACTCGGACAAC 1296**

**Drosophila_Aa_AF295933 GAGCGCAACTGCCGCATTCCGTGCAAGCAGACTAAGACATTCTCCACATACGCGGACAAC 1296**

**Spodoptera_FJ754276 GAGCGCAACTGTAAGATCCCGTGCAAGCAGTCGCAGACGTTCACCACGTACTCGGACAAC 1296**

**Bombyx_AB035326 GAACGAAACTCAAAGATTCCGTGCAAACAGTCTCAGACATTCACGACGTACTCAGACAAC 1296**

**Paralvinella_EF580992 GAACGTAACACGACCATTCCTCACAAGACGTCTAAGACATTCACCACCTACTCTGATAAT 1311**

**B_plicatilis_HSP70-1_AB775784 AAACGCAACACCACCATACCCACCAAGCAGACACAGACGTTCACCACTTATGCGGACAAC 1302**

**B_plicatilis_HSP70-2_AB775785 AAACGCAACACCACCATACCCACCAAGCAGACACAGACGTTCACCACTTATGCGGACAAC 1302**

**A_brightwelli_HSP70_KX119429 AAGAGAAACACCACAATCCCGACTAAACAAACCCAAGTTTTCACTACTTATTCAGACAAC 1299**

**Wuchereria_AF167352 AAGCGAAATACCACTATTCCCACGAAAACCTCCCAAACTTTTACAACATACTCCGACAAC 1302**

**Setaria_AF079360 AAGCGAAACACTACCATCCCGACGAAAACGTCGCAAACCTTTACCACCTACTCTGATAAT 1302**

**C_elegans_HSP70A_M18540 AAGAGAAACACCACCATCCCAACCAAGACCGCTCAGACCTTCACAACCTATTCTGATAAC 1305**

**C_elegans_HSP-1_NM_070667 AAGAGAAACACCACCATCCCAACCAAGACCGCTCAGACCTTCACAACCTATTCTGATAAC 1305**

**M_nipponense_KC460343 AAAAGAAACACCACCATTCCAACCAAACAGACCCAGACCTTTACCACCTATTCTGACAAC 1302**

**M_nipponense_DQ660140 AAGCGTAACACAACAATCCCAACCAAGCAGACTCAGACATTCACCACTTATTCAGACAAT 1302**

**Mytilus_HSP70_AY861684 AAACGTAATACAACCATTCCAACAAAACAGACACAAACCTTCACTACCTACTCTGACAAT 1305**

**Perinereis_KU255783 AAGAGGAACACAACTATCCCCACCAAGCAGACACAGACCTTCACAACCTATTCCGACAAC 1299**

**Paralvinella_HSP70-2_EF580993 AAGCGCAACACGACCATTCCAACAAAACAGACCCAAACCTTCACCACCTACTCGGACAAT 1302**

**Haliotis_FJ812176 AAGAGGAACACAACCATCCCCACCAAACAGACCCAGACCTTCACAACATACTCAGACAAT 1302**

**Oncorhynchus_HSC71_AAB21658 AAACGTAACACCACCATCCCAACCAAGCAGACTCAGACCTTCACCACCTACTCAGACAAC 1302**

**X_maculatus_HSC70_AB062115 AAGAGGAACACCACCATCCCCACCAAGCAGACCCAGACCTTCACAACTTACTCCGACAAC 1302**

**Human_HSPA8_HSC71_NP_006588 AAGCGTAATACCACCATTCCTACCAAGCAGACACAGACCTTCACTACCTATTCTGACAAC 1302**

**Rattus_HSC71_NM_024351 AAGCGCAATACCACCATTCCCACCAAGCAGACCCAGACTTTCACCACCTACTCTGACAAC 1302**

**Pelodiscus_HSC70_HQ219723 AAGCGGAACACTACAATTCCCACCAAGCAGACTCAGACATTCACCACATACTCTGACAAC 1302**

**Bos_HSP70_U09861 AAGCGCAACTCCACCATCCCCACGAAGCAGACGCAGATCTTCACCACCTACTCGGACAAC 1302**

**Human_HSPA1A_NM_005345 AAGCGCAACTCCACCATCCCCACCAAGCAGACGCAGATCTTCACCACCTACTCCGACAAC 1302**

**Rattus_HSP70_1A_NM_031971 AAGCGCAACTCCACCATCCCCACCAAGCAGACGCAGACCTTCACCACCTACTCGGACAAC 1302**

**X_maculatus_HSP70-1_AB062113 AAGCGAAACACCACCATCCCCACAAAGCAGACTCAGATCTTCTCCACATACTCAGATAAC 1308**

**X_maculatus_HSP70-2_AB062114 AAACGCAACACCACCGTCCCCACCAAGCAGACCCAGGTGTTCAGCACCTACTCCGACAAC 1308**

**Human_HSPA1L_NM_005527 AAGCGCAACTCCACCATCCCCACCAAGCAGACACAGATTTTCACCACCTACTCTGACAAC 1308**

**Human_HSPA6_NP_002146 CAGAGGAACGCCACTATCCCCACCAAGCAGACCCAGACTTTCACCACCTACTCGGACAAC 1308**

**Alligator_HSP70_AB306279 AAGCGCAACACCACCATCCCCACCAAGCAGACGCAGACCTTCACCACCTACTCGGACAAC 1311**

*** * ** * ** ** * * ** ** ** * ** ****

**Drosophila_Bb_AF295957 CAGCCCGGAGTCTCCATCCAGGTGTATGAGGGCGAACGTGCGATGACGAAGGACAACAAT 1356**

**Drosophila_Aa_AF295933 CAGCCCGGAGTCTCCATCCAGGTGTATGAGGGCGAACGTGCGATGACGAAGGACAACAAT 1356**

**Spodoptera_FJ754276 CAGCCAGCGGTCACGATCCAAGTGTACGAGGGCGAGCGAGCGATGACCAAGGACAACAAC 1356**

**Bombyx_AB035326 CAGCCGGCCGTCACCATCCAGGTGTACGAGGGAGAGAGAGCGATGACAAAGGACAACAAC 1356**

**Paralvinella_EF580992 CAGCCTGCAGTGACCATCCAAGTGTACGAGGGCGAGCGAGCGATGACCAAAGACAACAAC 1371**

**B_plicatilis_HSP70-1_AB775784 CAGCCCGGCGTGTTGATTCAAGTGTACGAGGGCGAGAGGGCCATGACCAAGGACAATCAT 1362**

**B_plicatilis_HSP70-2_AB775785 CAGCCCGGCGTGTTGATTCAAGTGTACGAGGGCGAGAGGGCCATGACCAAGGACAATCAT 1362**

**A_brightwelli_HSP70_KX119429 CAGCCGGGAGTGTTGATTCAAGTCTACGAAGGCGAAAGAGCCATGACTAAAGACAACAAT 1359**

**Wuchereria_AF167352 CAGCCTGGTGTCCTTATTCAGGTTTACGAAGGTGAACGTGCTTTGACCAAAGACAATAAC 1362**

**Setaria_AF079360 CAGCCAGGTGTTCTTATTCAGGTTTATGAAGGCGAACGTGCTATGACCAAGGACAATAAT 1362**

**C_elegans_HSP70A_M18540 CAACCAGGAGTGTTGATCCAGGTTTACGAAGGAGAACGTGCCATGACCAAGGACAACAAC 1365**

**C_elegans_HSP-1_NM_070667 CAACCAGGAGTGTTGATCCAGGTTTACGAAGGAGAACGTGCCATGACCAAGGACAACAAC 1365**

**M_nipponense_KC460343 CAGCCTGGAGTGCTCATTCAAGTTTACGAAGGAGAACGTGCCATGACAAAAGACAATAAC 1362**

**M_nipponense_DQ660140 CAGCCTGGTGTGTTAATTCAAGTTTATGAAGGAGAGCGTGCAATGACAAAAGATAACAAC 1362**

**Mytilus_HSP70_AY861684 CAGCCTGGTGTATTAATCCAGGTTTATGAAGGAGAGAGAGCTATGACCAAGGACAACAAC 1365**

**Perinereis_KU255783 CAGCCAGGTGTGTTGATCCAAGTTTACGAGGGTGAGCGTGCCATGACCAAGGACAACAAC 1359**

**Paralvinella_HSP70-2_EF580993 CAGCCCGGTGTCTTGATCCAGGTGTACGAGGGAGAGCGTGCCATGACCAAAGACAACAAC 1362**

**Haliotis_FJ812176 CAACCTGGTGTGCTGATTCAGGTATTCGAAGGTGAGAGAGCCATGACAAAGGATAACAAC 1362**

**Oncorhynchus_HSC71_AAB21658 CAGCCTGGTGTGCTCATTCAGGTGTATGAGGGTGAGAGGGCCATGACCAAGGACAACAAC 1362**

**X_maculatus_HSC70_AB062115 CAGCCTGGCGTGCTCATTCAGGTGTTTGAGGGCGAAAGGGCAATGACCAAAGATAACAAC 1362**

**Human_HSPA8_HSC71_NP_006588 CAGCCTGGTGTGCTTATTCAGGTTTATGAAGGCGAGCGTGCCATGACAAAGGATAACAAC 1362**

**Rattus_HSC71_NM_024351 CAGCCAGGTGTACTCATCCAGGTGTATGAAGGTGAAAGGGCCATGACCAAGGACAACAAC 1362**

**Pelodiscus_HSC70_HQ219723 CAGCCTGGTGTACTCATTCAGGTCTACGAAGGGGAGAGAGCCATGACAAAAGACAACAAC 1362**

**Bos_HSP70_U09861 CAGCCGGGCGTGCTGATCCAGGTGTACGAGGGCGAGAGGGCCATGACGCGGGACAACAAC 1362**

**Human_HSPA1A_NM_005345 CAACCCGGGGTGCTGATCCAGGTGTACGAGGGCGAGAGGGCCATGACGAAAGACAACAAT 1362**

**Rattus_HSP70_1A_NM_031971 CAGCCCGGGGTGCTGATCCAGGTGTACGAGGGCGAGAGGGCCATGACGCGCGACAACAAC 1362**

**X_maculatus_HSP70-1_AB062113 CAGCCAGGAGTCCTGATTCAGGTCTATGAAGGTGAGAGAGCCATGACAAAGGACAACAAT 1368**

**X_maculatus_HSP70-2_AB062114 CAGCCCGGGGTCCTGATCCAGGTCTACGAAGGGGAGAGAGCCATGACCAAGGACAACAAC 1368**

**Human_HSPA1L_NM_005527 CAACCCGGGGTGCTGATCCAGGTGTATGAGGGCGAGAGGGCCATGACAAAGGACAACAAC 1368**

**Human_HSPA6_NP_002146 CAGCCTGGGGTCTTCATCCAGGTGTATGAGGGTGAGAGGGCCATGACCAAGGACAACAAC 1368**

**Alligator_HSP70_AB306279 CAGAGCAGCGTGCTGGTGCAGGTGTACGAGGGCGAGCGCGCCATGACCAAGGACAACAAC 1371**

**** ** * ** ** * ** ** ** * ** **** ** ** ***

**Drosophila_Bb_AF295957 GCATTGGGCACCTTCGATCTGTCCGGCATTCCACCTGCACCAAGGGGTGTGCCCCAGATA 1416**

**Drosophila_Aa_AF295933 GCATTGGGCACCTTCGATCTGTCCGGCATTCCACCTGCACCAAGGGGTGTGCCCCAGATA 1416**

**Spodoptera_FJ754276 CTGCTGGGCACGTTCGACTTGACCGGCATCCCGCCCGCGCCTCGCGGTGTCCCTAAGATC 1416**

**Bombyx_AB035326 CTACTGGGCACGTTCGACCTGACGGGGATCCCGCCCGCGCCGCGAGGAGTGCCCAAGATC 1416**

**Paralvinella_EF580992 CTCCTCGGACGATTTGAGCTCAGCGGTATTCCGCCAGCTCCACGCGGAATTCCCAAGATC 1431**

**B_plicatilis_HSP70-1_AB775784 TTGTTGGGCAAGTTTGAGCTGTCGGGCATACCGCCGGCGCCCCGCGGCGTGCCCCAAATC 1422**

**B_plicatilis_HSP70-2_AB775785 TTGTTGGGCAAGTTTGAGCTGTCGGGCATACCGCCGGCGCCCCGCGGCGTGCCCCAAATC 1422**

**A_brightwelli_HSP70_KX119429 TTACTCGGTAAATTTGAGCTTACTGGCATTCCACCGGCGCCAAGAGGTGTTCCTCAAATC 1419**

**Wuchereria_AF167352 TTGCTTGGTAAATTCGAGTTGTCTGGGATTCCGCCTGCTCCTCGCGGTGTGCCGCAGATT 1422**

**Setaria_AF079360 TTGCTCGGTAAATTTGAGTTGTCTGGAATCCCGCCTGCTCCTCGTGGTGTGCCACAAATA 1422**

**C_elegans_HSP70A_M18540 TTGCTCGGAAAGTTCGAGCTCTCCGGAATCCCACCAGCACCACGCGGAGTCCCACAAATC 1425**

**C_elegans_HSP-1_NM_070667 TTGCTCGGAAAGTTCGAGCTCTCCGGAATCCCACCAGCACCACGCGGAGTCCCACAAATC 1425**

**M_nipponense_KC460343 ATTTTGGGTAAGTTTGAGCTGACTGGTATTCCCCCAGCGCCAAGGGGAGTGCCACAAATC 1422**

**M_nipponense_DQ660140 CTGCTAGGTAAATTTGAACTGTCTGGTATTCCTCCAGCTCCACGAGGAGTGCCACAAATT 1422**

**Mytilus_HSP70_AY861684 TTGCTTGGAAAGTTTGAATTAACTGGAATACCTCCAGCACCAAGAGGTGTGCCCCAGATT 1425**

**Perinereis_KU255783 CTCTTGGGTAAATTCGAGCTGTCCGGCATTCCCCCTGCTCCTCGTGGCGTTCCCCAGATC 1419**

**Paralvinella_HSP70-2_EF580993 CTGCTGGGAAAGTTCGAGCTGACTGGCATTCCACCTGCTCCTCGTGGTGTGCCACAGATA 1422**

**Haliotis_FJ812176 ATCTTGGGCAAGTTTGAGCTGACTGGAATTCCCCCAGCACCCAGAGGTGTCCCACAGATT 1422**

**Oncorhynchus_HSC71_AAB21658 CTGTTGGGCAAGTTTGAGCTGACTGGAATCCCCCCTGCACCTCGCGGTGTTCCTCAGATT 1422**

**X_maculatus_HSC70_AB062115 CTGCTCGGGAAATTTGAGCTGACGGGAATACCGCCAGCTCCTCGGGGGGTCCCTCAGATT 1422**

**Human_HSPA8_HSC71_NP_006588 CTGCTTGGCAAGTTTGAACTCACAGGCATACCTCCTGCACCCCGAGGTGTTCCTCAGATT 1422**

**Rattus_HSC71_NM_024351 CTGCTTGGGAAGTTTGAGCTCACAGGCATACCTCCAGCACCCCGTGGGGTTCCTCAGATT 1422**

**Pelodiscus_HSC70_HQ219723 TTGCTGGGCAAGTTTGAGCTGACTGGCATTCCTCCAGCTCCAAGAGGTGTACCTCAAATT 1422**

**Bos_HSP70_U09861 CTGCTGGGGCGCTTCGAGCTGAGCGGCATCCCGCCGGCCCCGCGGGGGGTGCCCCAGATC 1422**

**Human_HSPA1A_NM_005345 CTGTTGGGGCGCTTCGAGCTGAGCGGCATCCCTCCGGCCCCCAGGGGCGTGCCCCAGATC 1422**

**Rattus_HSP70_1A_NM_031971 CTGCTGGGGCGCTTCGAGTTGAGCGGCATCCCGCCGGCTCCCAGGGGCGTGCCCCAGATC 1422**

**X_maculatus_HSP70-1_AB062113 CTCCTGGGGAAGTTTGAGCTCTCTGGTCTTCCTCCTGCTCCCAGAGGCGTGCCACAGATC 1428**

**X_maculatus_HSP70-2_AB062114 CTGCTGGGCAGGTTTGAGCTGACGGGAATCCCGCCCGCTCCACGAGGGGTCCCGCAGATC 1428**

**Human_HSPA1L_NM_005527 CTGCTGGGGCGGTTTGACCTGACTGGAATCCCTCCAGCACCCAGGGGAGTTCCTCAGATC 1428**

**Human_HSPA6_NP_002146 CTGCTGGGGCGTTTTGAACTCAGTGGCATCCCTCCTGCCCCACGTGGAGTCCCCCAGATA 1428**

**Alligator_HSP70_AB306279 CTGCTGGGCAAGTTCGACCTGACCGGCATCCCGCCCGCGCCGCGCGGCGTGCCGCAGATC 1431**

*** ** ** ** * ** * ** ** ** ** * ** * ** * ****

**Drosophila_Bb_AF295957 GAAGTAACCTTCGACTTGGACGCCAATGGAATCCTGAACGTCAGCGCCAAGGAGATGAGC 1476**

**Drosophila_Aa_AF295933 GAAGTTACCTTCGACTTGGACGCCAATGGAATCCTGAACGTCAGCGCCAAGGAGATGAGC 1476**

**Spodoptera_FJ754276 GACGTCACTTTCGACCTGGACGCGAACGGCATTCTCAACGTGTCGGCCAAAGAGAACAGC 1476**

**Bombyx_AB035326 GACGTGACGTTCGACATGGACGCTAACGGCATCCTGAACGTGTCGGCCAAGGAGAACAGC 1476**

**Paralvinella_EF580992 GACGTCACATTCGACATCGACGCCAACGGAATCCTACACGTGACGGCCAAAGACCAAAGT 1491**

**B_plicatilis_HSP70-1_AB775784 GAGGTCACATTTGACATTGATGCCAATGGTATTCTGAATGTGTCCGCCGCAGACAAATCG 1482**

**B_plicatilis_HSP70-2_AB775785 GAGGTCACATTTGACATTGATGCCAATGGTATTCTGAATGTGTCCGCTGCAGACAAATCG 1482**

**A_brightwelli_HSP70_KX119429 GAGGTCACTTTTGACATCGATGCCAACGGTATTTTGAACGTTAGCGCTTGTGATAAGTCA 1479**

**Wuchereria_AF167352 GAAGTCGACTTTGATATTGATGCCAACGGTATTCTGAATGTTTCCGCCCAGGATAAATCC 1482**

**Setaria_AF079360 GAAGTTACTTTTGATATTGATGCAAACGGTATCTTGAATGTTTCTGCGCAAGACAAATCT 1482**

**C_elegans_HSP70A_M18540 GAAGTCACTTTCGATATTGACGCCAACGGAATCTTGAACGTCTCTGCCACTGACAAGTCC 1485**

**C_elegans_HSP-1_NM_070667 GAAGTCACTTTCGATATTGACGCCAACGGAATCTTGAACGTCTCTGCCACTGACAAGTCC 1485**

**M_nipponense_KC460343 GAAGTAACATTTGATATTGATGCTAATGGTATTCTAAATGTATCTGCTGCTGACAAGTCC 1482**

**M_nipponense_DQ660140 GAAGTAACTTTTGATATTGATGCCAATGGTATCTTGAATGTATCTGCTGCAGACAAGTCA 1482**

**Mytilus_HSP70_AY861684 GAAGTGACCTTTGACATTGATGCCAATGGTATCCTGAATGTATCTGCAGTAGATAAGAGT 1485**

**Perinereis_KU255783 GAGGTTACCTTCGATATTGATGCCAACGGTATCCTGAATGTCACTGCTGCGGACAAGAGC 1479**

**Paralvinella_HSP70-2_EF580993 GAGGTCACATTCGATATCGATGCCAATGGTATCCTTAACGTGTCTGCTGTAGACAAGAGT 1482**

**Haliotis_FJ812176 GAAGTAACCTTTGATATTGATGCCAACGGTATCCTCAATGTATCAGCTGTAGACAAGAGC 1482**

**Oncorhynchus_HSC71_AAB21658 GAGGTCACATTTGACATTGATGCTAACGGCATCATGAACGTGTCTGCTGCTGACAAGAGC 1482**

**X_maculatus_HSC70_AB062115 GAGGTGACATTCGATATCGACGCTAACGGCATCATGAACGTCTCCGCTGTCGACAAGAGC 1482**

**Human_HSPA8_HSC71_NP_006588 GAAGTCACTTTTGACATTGATGCCAATGGTATACTCAATGTCTCTGCTGTGGACAAGAGT 1482**

**Rattus_HSC71_NM_024351 GAGGTTACTTTTGACATTGATGCCAATGGCATCCTCAATGTTTCTGCTGTAGATAAGAGC 1482**

**Pelodiscus_HSC70_HQ219723 GAAGTAACATTTGATATTGATGCCAATGGCATCCTGAATGTGTCTGCTGTGGACAAGAGC 1482**

**Bos_HSP70_U09861 GAGGTGACCTTCGACATCGACGCCAATGGCATCCTGAACGTCACGGCCACGGACAAGAGC 1482**

**Human_HSPA1A_NM_005345 GAGGTGACCTTCGACATCGATGCCAACGGCATCCTGAACGTCACGGCCACGGACAAGAGC 1482**

**Rattus_HSP70_1A_NM_031971 GAGGTGACCTTCGACATCGACGCCAACGGCATCCTGAACGTCACGGCCACTGACAAGAGC 1482**

**X_maculatus_HSP70-1_AB062113 GAAGTTACCTTTGATATCGACGCCAACGGCATCCTAAATGTGTCTGCCGTCGACAAAAGC 1488**

**X_maculatus_HSP70-2_AB062114 GAGGTCACCTTTGACGTGGACGCCAACGGCATCCTGAACGTGTCCGCGGTGGACAAAAGC 1488**

**Human_HSPA1L_NM_005527 GAGGTGACGTTTGACATTGATGCCAATGGTATTCTCAATGTCACAGCCACGGACAAGAGC 1488**

**Human_HSPA6_NP_002146 GAGGTGACTTTTGACATTGATGCTAATGGCATCCTGAGCGTGACAGCCACTGACAGGAGC 1488**

**Alligator_HSP70_AB306279 GAGGTCACTTTCGACATCGACGCCAACGGCATCCTCAACGTGAGCGCGGTCGACAAGAGC 1491**

**** ** ** ** * ** ** ** ** ** * ** ** ****

**Drosophila_Bb_AF295957 ACGGGCAAGGCCAAGAACATCACGATCAAGAACGACAAGGGACGCCTCTCGCAGGCCGAG 1536**

**Drosophila_Aa_AF295933 ACGGGCAAGGCCAAGAACATCACGATCAAGAACGACAAGGGACGGCTCTCGCAGGCCGAG 1536**

**Spodoptera_FJ754276 ACCGGCCGAAGCAAGAACATCGTGATCAAGAACGACAAGGGACGCCTGTCGCAGGCCGAG 1536**

**Bombyx_AB035326 ACCGGCCGCAGCAAGAACATCGTGATCAAGAACGACAAGGGTCGTCTCTCGCAAGCGGAG 1536**

**Paralvinella_EF580992 ACTGGTAGGTCAAGTGATATCCACATCAAGAACGAGAAAGGTCGTTTGTCACAGGCCGAA 1551**

**B_plicatilis_HSP70-1_AB775784 ACTGGCAAGACGAACAAAATCACCATCACCAATGACAAGGGAAGACTTAGCAAAGAGGAG 1542**

**B_plicatilis_HSP70-2_AB775785 ACTGGCAAGACGAACAAAATCACCATCACCAATGACAAGGGAAGACTTAGCAAAGAGGAC 1542**

**A_brightwelli_HSP70_KX119429 ACTGGCAAACAGAACAAGATCACGATCACGAACGACAAAGGAAGACTCAGCAAAGAGGAC 1539**

**Wuchereria_AF167352 ACAGGCAAACAAAACAAAATTACCATTACCAATGATAAAGGACGTTTGTCAAAGGATGAG 1542**

**Setaria_AF079360 ACTGGTAAACAAAATAAGATAACTATTACTAATGACAAGGGAAGGTTATCGAAGGATGAG 1542**

**C_elegans_HSP70A_M18540 ACCGGAAAGGCAAAACAGATCACCATCACCAACGACAAGGATCGCTTTTCCAAGGATGAC 1545**

**C_elegans_HSP-1_NM_070667 ACCGGAAAGCAAAACAAGATCACCATCACCAACGACAAGGGACGTCTTTCCAAGGATGAC 1545**

**M_nipponense_KC460343 ACAGGCAAAGAGAACAAGATCACCATCACTAATGATAAGGGCCGCTTGAGCAAGGAAGAG 1542**

**M_nipponense_DQ660140 ACTGGTAAGGAGAACAAAATCACAATCACAAATGACAAGGGTCGTCTCAGCAAAGAAGAA 1542**

**Mytilus_HSP70_AY861684 ACTGGTAAAGAGAACAAAATCACCATCACTAACGACAAAGGTCGTTTGAGCAAAGAAGAA 1545**

**Perinereis_KU255783 ACCGGCAAGGAGAACAAGATTACCATCACCAACGACAAAGGCCGTCTCAGTAAGGAAGAT 1539**

**Paralvinella_HSP70-2_EF580993 ACTGGTAGAGAGAACAAGATCACTATCACCAATGATAAGGGTCGTCTAAGCAAAGAGGAG 1542**

**Haliotis_FJ812176 ACTATGAAAGAGAACAAGATCACCATCACAAACGACAAAGGTCGCTTGTCGAAGGAAGAG 1542**

**Oncorhynchus_HSC71_AAB21658 ACTGGGAAGGAGAACAAGATCACCATCACAAATGACAAGGGTCGCCTGAGCAAGGAGGAC 1542**

**X_maculatus_HSC70_AB062115 ACCGGGAAGGAGAACAAAATCACCATCACAAACGACAAGGGCCGCTTGAGCAAAGAGGAT 1542**

**Human_HSPA8_HSC71_NP_006588 ACGGGAAAAGAGAACAAGATTACTATCACTAATGACAAGGGCCGTTTGAGCAAGGAAGAC 1542**

**Rattus_HSC71_NM_024351 ACAGGAAAGGAGAACAAGATCACCATCACCAATGACAAGGGCCGCTTGAGTAAGGAGGAT 1542**

**Pelodiscus_HSC70_HQ219723 ACTGGCAAGGAGAACAAGATCACCATTACCAATGACAAAGGGCGATTAAGCAAGGAAGAC 1542**

**Bos_HSP70_U09861 ACGGGCAAGGCCAACAAGATCACCATCACCAACGACAAGGGCCGGCTGAGCAAGGAGGAG 1542**

**Human_HSPA1A_NM_005345 ACCGGCAAGGCCAACAAGATCACCATCACCAACGACAAGGGCCGCCTGAGCAAGGAGGAG 1542**

**Rattus_HSP70_1A_NM_031971 ACCGGCAAGGCCAACAAGATCACCATCACCAACGACAAGGGCCGCCTGAGCAAGGAGGAG 1542**

**X_maculatus_HSP70-1_AB062113 ACGGGCAAAGAAAACAAAATCACAATCACCAACGACAAGGGCCGACTCAGCAAAGATGAG 1548**

**X_maculatus_HSP70-2_AB062114 ACCGGCAAAGAGAACAAGATCACCATCGCCAACGACAAGGGCCGACTGAGCAAAGACGAG 1548**

**Human_HSPA1L_NM_005527 ACCGGCAAGGTGAACAAGATCACCATCACCAATGACAAGGGCCGCCTGAGCAAGGAGGAG 1548**

**Human_HSPA6_NP_002146 ACAGGTAAGGCTAACAAGATCACCATCACCAATGACAAGGGCCGGCTGAGCAAGGAGGAG 1548**

**Alligator_HSP70_AB306279 ACGGGCAAGGAGAACAAGATCACCATCACCAACGACAAGGGCCGGCTCAGCAAGGACGAC 1551**

**** * * ** ** ** ** ** * * * * * ****

**Drosophila_Bb_AF295957 ATTGATCGCATGGTGAACGAGGCTGAGAAGTACGCCGACGAGGACGAAAAGCATCGCCAG 1596**

**Drosophila_Aa_AF295933 ATTGATCGCATGGTGAACGAGGCTGAAAAGTACGCCGACGAGGACGAGAAGCATCGCCAG 1596**

**Spodoptera_FJ754276 ATCGAGCGCATGCTCGCCGAGGCCGAGAAGTACAAGGACGAGGACGAGAAGCAGAGGCAG 1596**

**Bombyx_AB035326 ATCGATCGCATGCTGTCCGAGGCAGAGCGGTACAAGGAGGAAGACGAGAAGCAGAGACAG 1596**

**Paralvinella_EF580992 ATCGACAGGATGTTGGCCGAAGCTGAGAAATATCGCGAAGAAGACGAGAAACAACGAGAA 1611**

**B_plicatilis_HSP70-1_AB775784 ATCGACCGAATGGTCAACGAGGCCGAGAAGTACAAGAAGGATGACGAGGAGCAGAGAGAT 1602**

**B_plicatilis_HSP70-2_AB775785 ATCGATCGAATGGTCAATGAGGCCGAGAAGTACAAGAAAGATGACGAGGAGCAGAGAGAT 1602**

**A_brightwelli_HSP70_KX119429 ATTGAGAGAATGGTCAACGATGCCGAGAAGTATAAAAAAGACGACGAGCAACAAAGAGAG 1599**

**Wuchereria_AF167352 ATCGAGCGGATGGTACAGGAAGCTGAGAAATACAAGGCGGATGATGAAGCACAGAAGGAT 1602**

**Setaria_AF079360 ATCGAACGGATGGTGCAAGAAGCTGAGAAATATAAGGCAGATGATGAAGCTCAGAAAGAC 1602**

**C_elegans_HSP70A_M18540 ATTGAACGCATGGTCAACGAAGCTGAGAAATACAAGGCTGACGATGAGGCCCAAAAGGAC 1605**

**C_elegans_HSP-1_NM_070667 ATTGAACGCATGGTCAACGAAGCTGAGAAATACAAGGCTGACGATGAGGCCCAAAAGGAC 1605**

**M_nipponense_KC460343 ATTGAGCGGATGGTGCAAGAGGCCGAGAAGTACAAAGCTGACGATGAGAAACAGCGGGAG 1602**

**M_nipponense_DQ660140 ATTGAACGTATGGTTCAAGAAGCTGAAAAGTACAAAGCTGATGATGAGAAGCAACGTGAT 1602**

**Mytilus_HSP70_AY861684 ATTGAACGCATGGTCAATGATGCTGAGAAATACAAGGCAGAAGACGAGAAACAGAAGGAC 1605**

**Perinereis_KU255783 ATTGACCGCATGGTTAACGAAGCCGAGCGTCTCAAGGCTGAAGATGATGCGCAGAGAGAA 1599**

**Paralvinella_HSP70-2_EF580993 ATTGAACGTATGGTCAAGGATGCTGAGAGGTACAAGCAAGAAGATGAAAGTCAGAAGGAT 1602**

**Haliotis_FJ812176 ATTGAGCGAATGGTTAATGAGGCAGAGAACTACAAGGCCGAGGATGAGAAGCAGAAGGAT 1602**

**Oncorhynchus_HSC71_AAB21658 ATTGAGCGCATGGTCCAGGAGGCTGAGAAGTACAAGTGTGAGGATGATGTGCAGCGTGAC 1602**

**X_maculatus_HSC70_AB062115 ATTGAGCGCATGGTTCACGAAGCGGAGACATACAGGGCTGAAGACGACTTGCAGCGAGAA 1602**

**Human_HSPA8_HSC71_NP_006588 ATTGAACGTATGGTCCAGGAAGCTGAGAAGTACAAAGCTGAAGATGAGAAGCAGAGGGAC 1602**

**Rattus_HSC71_NM_024351 ATTGAGCGCATGGTCCAAGAAGCTGAGAAGTACAAAGCTGAGGATGAGAAGCAGAGAGAT 1602**

**Pelodiscus_HSC70_HQ219723 ATTGAACGCATGGTGCAGGAAGCAGAAAAATACAAAGCAGAAGATGAGAAACAGCGTGAC 1602**

**Bos_HSP70_U09861 ATCGAGCGCATGGTGCAGGAGGCGGAAAAGTACAAGGCGGAGGACGAGGTGCAGCGCGAG 1602**

**Human_HSPA1A_NM_005345 ATCGAGCGCATGGTGCAGGAGGCGGAGAAGTACAAAGCGGAGGACGAGGTGCAGCGCGAG 1602**

**Rattus_HSP70_1A_NM_031971 ATCGAGCGCATGGTGCAGGAGGCCGAGCGCTACAAGGCGGAGGACGAGGTGCAGCGCGAG 1602**

**X_maculatus_HSP70-1_AB062113 ATCGAGAAGATGGTGCAGGATGCAGACAAGTACAAGGCGGAGGATGACCAGCAGAGAGAA 1608**

**X_maculatus_HSP70-2_AB062114 ATCGAGAGGATGGTGCAGGACGCCGAGAAGTACAAAGCCGAGGACGAGCTGCAGAGGGAC 1608**

**Human_HSPA1L_NM_005527 ATTGAGCGCATGGTTCTGGATGCTGAGAAATATAAAGCTGAAGATGAGGTCCAGAGGGAG 1608**

**Human_HSPA6_NP_002146 GTGGAGAGGATGGTTCATGAAGCCGAGCAGTACAAGGCTGAGGATGAGGCCCAGAGGGAC 1608**

**Alligator_HSP70_AB306279 ATCGACCGCATGGTGCAGGAGGCCGAGAAGTACAAGGCGGAGGACGACGCCAACCGCGAG 1611**

*** ** *** * ** ** ** ** ** ** * ***

**Drosophila_Bb_AF295957 CGCATAACCTCTAGAAATGCTCTGGAGAGCTACGTATTCAACGTAAAGCAGTCCGTGGAG 1656**

**Drosophila_Aa_AF295933 CGAATAACCTCTAGAAATGCCCTGGAGAGCTACGTCTTCAATGTGAAGCAGGCCGTGGAA 1656**

**Spodoptera_FJ754276 CGCGTAGCGTCTCGGAACCAGCTCGAGTCGTACGTGTTCAGCGTGAAGCAGGCGCTGGAC 1656**

**Bombyx_AB035326 CGCGTGGCCGCTCGGAACCAGCTCGAATTGTATTTGTTCAGCGTGAAGCAGGCGCTCGAC 1656**

**Paralvinella_EF580992 CGTATCTCGGCCAGAAACAGCTTGGAACAATACATCTACTCATACAAAGAGGCCGCTACT 1671**

**B_plicatilis_HSP70-1_AB775784 AAGGTGGCCGCCAAGAACTCGCTTGAGTCCTACTGCTTTAACATGAAACAAACGGTGGAG 1662**

**B_plicatilis_HSP70-2_AB775785 AAGGTGGCCGCCAAGAACTCGCTTGAGTCCTACTGCTTTAACATGAAACAAACGGTGGAG 1662**

**A_brightwelli_HSP70_KX119429 AGAATCACTGCCAAGAACTCGTTGGAATCCTACTGCTTCAACATGAAACAAACCATCGAG 1659**

**Wuchereria_AF167352 CGTATTGCGGCAAAAAATGCTCTGGAATCGTACGCATTCAATATGAAACAAACGATCGAG 1662**

**Setaria_AF079360 CGTATTGCGGCAAAGAATGCTCTTGAATCATATGCGTTCAATATGAAGCAAACAATTGAG 1662**

**C_elegans_HSP70A_M18540 CGTATTGGAGCCAAGAACGGACTCGAGTCATACGCCTTCAACCTTAAGCAGACCATTGAG 1665**

**C_elegans_HSP-1_NM_070667 CGTATTGGAGCCAAGAACGGACTCGAGTCATACGCCTTCAACCTTAAGCAGACCATTGAG 1665**

**M_nipponense_KC460343 CGTATCTCTGCCAAGAACAACCTGGAGTCTTACTGTTTCAATATGAAGTCCACTGTTGAA 1662**

**M_nipponense_DQ660140 CGCATTGCAGCAAAGAATAGTTTGGAATCTTACTGCTTCAACATGAAATCCACAGTGGAA 1662**

**Mytilus_HSP70_AY861684 CGTATCACCGCCAAAAATAGTCTAGAAAGCTACTCATTTAACATGAAACAAACAGTTGAA 1665**

**Perinereis_KU255783 CGTATCACAGCCAAGAACCAGCTGGAGAGCTATGCGTTCAACATGAAATCAACTGTCGAG 1659**

**Paralvinella_HSP70-2_EF580993 CGCATCCAAGCCAAGAATGCCCTGGAGAGCTATGCCTTTAACATGAAGTCGACTGTCGAG 1662**

**Haliotis_FJ812176 CGCATCCAGGCCAAGAACGGTTTGGAAAGCTATGCCTTCAACATGAAGTCGACTGTAGAG 1662**

**Oncorhynchus_HSC71_AAB21658 AAGGTCTCTTCTAAGAACTCCCTAGAGTCCTACGCTTTCAACATGAAGTCTACTGTGGAG 1662**

**X_maculatus_HSC70_AB062115 AAGGTGGTGGCCAAGAACAGCCTGGAGTCCTACACTTTCAACATGAGGTCCACCATAGAG 1662**

**Human_HSPA8_HSC71_NP_006588 AAGGTGTCATCCAAGAATTCACTTGAGTCCTATGCCTTCAACATGAAAGCAACTGTTGAA 1662**

**Rattus_HSC71_NM_024351 AAGGTTTCCTCTAAGAACTCGCTGGAGTCTTATGCTTTCAACATGAAAGCAACTGTTGAG 1662**

**Pelodiscus_HSC70_HQ219723 AAAGTTTCCTCTAAGAATTCTCTAGAATCATATGCCTTTAATATGAAGGCTACAGTTGAA 1662**

**Bos_HSP70_U09861 AGGGTGTCTGCCAAGAACGCGCTGGAGTCGTACGCCTTCAACATGAAGAGCGCCGTGGAG 1662**

**Human_HSPA1A_NM_005345 AGGGTGTCAGCCAAGAACGCCCTGGAGTCCTACGCCTTCAACATGAAGAGCGCCGTGGAG 1662**

**Rattus_HSP70_1A_NM_031971 AGGGTGGCTGCCAAGAATGCGCTCGAGTCCTATGCCTTCAACATGAAGAGCGCCGTGGAG 1662**

**X_maculatus_HSP70-1_AB062113 AAGATCGCGGCAAAGAATTCCCTGGAGTCCTACGCTTACCACATGAAGAGCAGCGTCGAA 1668**

**X_maculatus_HSP70-2_AB062114 AAGATAGCTGCCAAGAACTCCCTGGAGTCCTACGCTTTCACCGTGAAGAGCAGCGTGGAG 1668**

**Human_HSPA1L_NM_005527 AAAATTGCTGCAAAGAATGCCTTAGAATCCTATGCTTTTAACATGAAGAGTGTTGTGAGT 1668**

**Human_HSPA6_NP_002146 AGAGTGGCTGCCAAAAACTCGCTGGAGGCCCATGTCTTCCATGTGAAAGGTTCTTTGCAA 1668**

**Alligator_HSP70_AB306279 CGGGTGGCGGCCAAGAACTCGCTCGAGTCCTACGCCTACAACATGAAGCAGACGGTGGAG 1671**

*** * ** * ** * * ***

Drosophila_Bb_AF295957 CAGGCGCC---CGCTGGCAAACTGGACGAGGCCGACAAGAACTCCGTCCTGGACAAGTGC 1713

Drosophila_Aa_AF295933 CAGGCACC---TGCTGGCAAATTGGACGAGGCTGACAAGAACTCCGTCTTGGACAAGTGC 1713

Spodoptera_FJ754276 GAGGCCGG------AGACAAGCTCTCGGAACAGGACAAGAGCACGGCGAGGGACGCGTGC 1710

Bombyx_AB035326 GAGGCCGG------CGACAAACTGAGCGACGCGGACAAGAGCACGGCGCGCGACGCGTGT 1710

Paralvinella_EF580992 GAGGC---CGATAGTTCTAAACTGTCACAGACTGATAAGGATCAAGTGATCAACAAATGT 1728

B_plicatilis_HSP70-1_AB775784 GACGAAAAGCTGGCTGCTAAGATTAGTGCCGACGACAAGAAGAAGATTTTGGACGCGTGT 1722

B_plicatilis_HSP70-2_AB775785 GACGAAAAGCTGGCTGCTAAGATTAGTGCAGATGACAAGAAGAAGATTTTGGACGCGTGT 1722

A_brightwelli_HSP70_KX119429 GACGAGAAATTGGCAGCTAAAGTCTCGGCTGATGAAAAGAAGAAGATTTTGGACGCGTGC 1719

Wuchereria_AF167352 GACGAGAAGTTAAAAGATAAAATTTCGGAAGAGGATAAGAAAAAAATTCAAGAAAAATGC 1722

Setaria_AF079360 GATGAAAAGCTGAGGGACAAACTATCTGAGGAGGATAAGAAGAAGATCCAGGAGAAGTGT 1722

C_elegans_HSP70A_M18540 GACGAGAAGCTCAAGGATAAGATCAGCCCAGAAGACAAGAAGAAGATCGAGGACAAGTGC 1725

C_elegans_HSP-1_NM_070667 GACGAGAAGCTCAAGGATAAGATCAGCCCAGAAGACAAGAAGAAGATCGAGGACAAGTGC 1725

M_nipponense_KC460343 GATGACAAATTCAAGGATAAGGTTCCTGAGGATGATCGCAACAAGATCATGGAAGCCTGT 1722

M_nipponense_DQ660140 GATGACAAGTTTAAGGACAAGGTGCCAGAGGAGGATCGCAACAAGATAATGGAAGCTTGC 1722

Mytilus_HSP70_AY861684 GATGAAAAACTCAAGGATAAAATCAGTGAAAGTGACAAAAAGGAAATCATGGACAAATGT 1725

Perinereis_KU255783 GACGAGAAACTGAAGGACAAGATTTCCGACGAGGACAAGACCAAGATCACTGAGAAATGC 1719

Paralvinella_HSP70-2_EF580993 GACGAGAAATTGAAGGACAAGATCAGTGATGCTGATAAGAAGACCATTACAGACAAATGT 1722

Haliotis_FJ812176 GATGAGAAACTGAAGGACAAGATCAGTGAAGATGACAAGAAAACCATCACTGACAAGTGC 1722

Oncorhynchus_HSC71_AAB21658 GATGAGAAACTGCAGGGGAAAATCAGTGACGAGGACAAGACTAAGATTCTGGAGAAGTGC 1722

X_maculatus_HSC70_AB062115 GACGAGAAGCTGAAGGATAAGATCAGCGAATCGGACAAGAAGAAGGTCCTGGACAAATGC 1722

Human_HSPA8_HSC71_NP_006588 GATGAGAAACTTCAAGGCAAGATTAACGATGAGGACAAACAGAAGATTCTGGACAAGTGT 1722

Rattus_HSC71_NM_024351 GATGAGAAACTTCAAGGCAAGATCAATGATGAAGACAAACAGAAGATTCTTGACAAGTGC 1722

Pelodiscus_HSC70_HQ219723 GATGAGAAGCTCCAGGGCAAGATCAGCAGTGAAGACAAGCAGAAAATCCTAGACAAATGT 1722

Bos_HSP70_U09861 GATGAGGGGCTGAAGGGCAAGATCAGCGAGGCGGACAAGAAGAAGGTGCTGGACAAGTGC 1722

Human_HSPA1A_NM_005345 GATGAGGGGCTCAAGGGCAAGATCAGCGAGGCGGACAAGAAGAAGGTGCTGGACAAGTGT 1722

Rattus_HSP70_1A_NM_031971 GACGAGGGTCTCAAGGGCAAGATCAGCGAGGCTGACAAGAAGAAGGTGCTGGACAAGTGC 1722

X_maculatus_HSP70-1_AB062113 GACGACGCCTTGAAAGGAAAAATCAGTGAGGAGGAGAAAAAGCTGGTTATTGACAAATGC 1728

X_maculatus_HSP70-2_AB062114 GACGAGAGCTTGAAGGGCAAGATCAGCCTTAGAGGACAAGAAGAAGGTGTGAACAAGTGC 1728

Human_HSPA1L_NM_005527 GATGAAGGTTTGAAGGGCAAGATTAGTGAGTCTGATAAAAATAAAATATTGGATAAATGC 1728

Human_HSPA6_NP_002146 GAGGAAAGCCTTAGGGACAAGATTCCCGAAGAGGACAGGCGCAAAATGCAAGACAAGTGT 1728

Alligator_HSP70_AB306279 GACGAGAAGCTGGCGGGCAAGATCGGCGACCAGGACAAGCAGCGTGTGCTCGACAAGTGC 1731

* * ** * * * **

Drosophila_Bb_AF295957 AACGAAACTATTCGATGGCTGGACAGCAACACCACCGCCGAGAAGGAGGAGTTCGACCAC 1773

Drosophila_Aa_AF295933 AACGACACTATCCGGTGGCTGGACAGCAACACCACCGCCGAGAAGGAGGAGTTCGACCAC 1773

Spodoptera_FJ754276 GACGACGCGCTCAAGTGGCTCGACAACAACACGCTGGCGGAGCAGGAGGAGTACGAGCAC 1770

Bombyx_AB035326 GACGAGGCGCTGCGGTGGCTGGACAACAACACTCTCGCTGACCAGGATGAATACGAGCAC 1770

Paralvinella_EF580992 GAGCAGACTGTTAAGTGGTTGGATAACAACACTTTGGCAGAAAAAGAGGAATACGAACAT 1788

B_plicatilis_HSP70-1_AB775784 GAAGAGGCCTTGAAATGGCTGGACTCGAACCAGACCGCTGAGAAGGACGAGTTCGAGCAC 1782

B_plicatilis_HSP70-2_AB775785 GAAGAGGCCTTGAAATGGCTGGACTCGAACCAGACCGCTGAGAAGGACGAGTTCGAGCAC 1782

A_brightwelli_HSP70_KX119429 GAGAACGCGCTCAAATGGCTGGACGCGAATCAAACGGCCGAGAAGGATGAGTTTGAGCAT 1779

Wuchereria_AF167352 GACGAAACAGTTAGGTGGTTGGATGGAAATCAAACAGCTGAAAAAGATGAATTTGAGCAT 1782

Setaria_AF079360 GATGAGACGGTCAGGTGGTTGGATGGGAATCAGACAGCTGAGAAGGATGAGTTTGAGCAT 1782

C_elegans_HSP70A_M18540 GACGAGATCTTGAAGTGGCTCGACAGCAACCAGACCGCAGAGAAGGAGGAGTTCGAGTCA 1785

C_elegans_HSP-1_NM_070667 GACGAGATCTTGAAGTGGCTCGACAGCAACCAGACCGCAGAGAAGGAGGAGTTCGAGCAC 1785

M_nipponense_KC460343 AATGATGCCATCAAGTGGCTCGACACTAATCAGCTTGGGGAGAAGGAAGAATATGAACAC 1782

M_nipponense_DQ660140 AATGATGCCATCAAATGGCTGGATACTAACCAGCTGGGAGAGAAGGAAGAATATGAACAT 1782

Mytilus_HSP70_AY861684 GACGAAATCATTAAATGGTTGGATGCCAACAATCTGGCTGAGAAGGAAGAATTTGAACAC 1785

Perinereis_KU255783 AACGAGGTCATCAGGTGGTTGGACTCCAACCAGACCGCAGAGAAGGACGAGTTTGAACAC 1779

Paralvinella_HSP70-2_EF580993 AACGATATCATAGCTTGGCTGGATGCTAACCAGCTCGCTGAAAAAGATGAATTTGAACAC 1782

Haliotis_FJ812176 AACGATGTTATCAGCTGGTTAGACTCTAACCAGTTGGCTGAGAAGGATGAGTTCGAACAC 1782

Oncorhynchus_HSC71_AAB21658 AACGAGGTCATCGGGTGGCTGGACAAGAACCAGACTGCTGAGAAGGAAGAGTATGAGCAC 1782

X_maculatus_HSC70_AB062115 AACGAGGTCATCAGCTGGCTCGACAAAAATCAGAGTGCAGAGAAAGAGGAGTTTGAGCAC 1782

Human_HSPA8_HSC71_NP_006588 AATGAAATTATCAACTGGCTTGATAAGAATCAGACTGCTGAGAAGGAAGAATTTGAACAT 1782

Rattus_HSC71_NM_024351 AACGAAATCATCAGCTGGCTGGATAAGAACCAGACTGCGGAGAAGGAAGAATTTGAGCAT 1782

Pelodiscus_HSC70_HQ219723 AACGAAATTATCAACTGGCTGGATAAGAATCAGACGGCTGAAAAAGAAGAGTTTGAACAT 1782

Bos_HSP70_U09861 CAGGAGGTGATTTCCTGGCTGGACGCCAACACCTTGGCGGAGAAGGACGAGTTTGAGCAC 1782

Human_HSPA1A_NM_005345 CAAGAGGTCATCTCGTGGCTGGACGCCAACACCTTGGCCGAGAAGGACGAGTTTGAGCAC 1782

Rattus_HSP70_1A_NM_031971 CAGGAGGTCATCTCCTGGCTGGACTCTAACACGCTGGCTGAGAAAGAGGAGTTCGTGCAC 1782

X_maculatus_HSP70-1_AB062113 AACCAGACAATTTCCTGGCTGGAGAACAACCAGCTGGCAGAGAAGGAGGAGTATGAACAT 1788

X_maculatus_HSP70-2_AB062114 CAGGAGACCATCAGCTGGCTGGAGAACAACCAGCTGGCTGAGAAAGACGAGTACCAACAC 1788

Human_HSPA1L_NM_005527 AACGAGCTCCTTTCGTGGCTGGAGGTCAATCAACTGGCAGAGAAAGATGAGTTTGATCAT 1788

Human_HSPA6_NP_002146 CGGGAAGTCCTTGCCTGGCTGGAGCACAACCAGCTGGCAGAGAAGGAGGAGTATGAGCAT 1788

Alligator_HSP70_AB306279 CAGGAGCTGATCGCCTGGCTGGACCGCAACCAGATGGCCGAGAAGGACGAGTACGAGCAC 1791

* * *** * ** ** * ** * ** ** *

Drosophila_Bb_AF295957 AAGATGGAGGAGCTCACTCGCCACTGTTCCCCTATCATGACCAAGATGCATCAGCAGGGA 1833

Drosophila_Aa_AF295933 AAGATGGAGGAGCTCACTCGCCACTGCTCCCCTATCATGACCAAGATGCATCAGCAGGGA 1833

Spodoptera_FJ754276 AAGCTGAAGGACGTGCAGCGCGTCTGCTCGCCCGTCATGGCCAAGATGCATGGGGCGGGC 1830

Bombyx_AB035326 AAGCTAAAGGATGTGCAGCGAGTGTGTTCGCCGGTCATGAGCAAGATGCACGGTGCGGCG 1830

Paralvinella_EF580992 CATTTGGAGGAGTTACACAAGGTCTGTTCTCCGATAATGGCCAAGCTACATGGATCACAG 1848

B_plicatilis_HSP70-1_AB775784 AAGATGAAAGAAGTGGAGAAAATTTGCTCGCCGATTATTACTCAGTTGTATCAGGGCGCT 1842

B_plicatilis_HSP70-2_AB775785 AAGATGAAAGAAGTGGAGAAAATTTGCTCGCCGATTATTACTCAGTTGTATCAGGGCGCT 1842

A_brightwelli_HSP70_KX119429 AAGTTGAAGGAGATCGAGAAGACTTGTTCGCCGATCATTACCAAGTTGTATCAGGGTGGT 1839

Wuchereria_AF167352 CGCCAAAAAGAATTGGAATCTGTTTGCAATCCGATTATTACAAAACTCTACCAGAGCGCT 1842

Setaria_AF079360 CGTCAGAAAGAGTTGGAAGCAGTTTCGAATCCAATCATTACGAAACTGTACCAGAGTGCT 1842

C_elegans_HSP70A_M18540 CAACAGAAGGATTTGGAAGGATTGGCCAAGCCCGATCTTTCCAAGCTTTACCAGAGTGCC 1845

C_elegans_HSP-1_NM_070667 CAACAGAAGGATTTGGAAGGATTGGCCAACCCAATCATTTCCAAGCTTTACCAGAGTGCC 1845

M_nipponense_KC460343 AAACTGAAGGAAATTGAACAGATATGTAATCCTATCATCACCAAAATGTACCAAGCAGCT 1842

M_nipponense_DQ660140 AAGTTGAAGGAAATTGAGCAAATATGTAATCCTATCATCACCAAAATGTACCAAGCAGCT 1842

Mytilus_HSP70_AY861684 AAACAGAAAGAACTTGAGGGAGTGTGTAATCCAATCATCACTAAACTGTACCAGTCTGCT 1845

Perinereis_KU255783 CAACAGAAGGAATTGGAAGGCATCTGCATGCCTATCATCACCAAGTTGTACCAGGCAGGT 1839

Paralvinella_HSP70-2_EF580993 CAACAAAAGGAACTGGAGAAGATCTGTATGCCGATCATTACAAAGCTGTACCAGGCAGGT 1842

Haliotis_FJ812176 AAGCAGAAGGAGCTGGAGGGTGTGTGTAATCCAATCATCACCAAGCTGTACCAGGCAGCT 1842

Oncorhynchus_HSC71_AAB21658 CACCAGAAGGAGTTGGAGAAGGTGTGCAACCCCATCATCACCAAGCTGTACCAGGGTGCT 1842

X_maculatus_HSC70_AB062115 CAGCAGAAGGAGCTTGAGAAGTTGTGCAACCCGATTATGACCAAACTGTACCAGAGTGAA 1842

Human_HSPA8_HSC71_NP_006588 CAACAGAAAGAGCTGGAGAAAGTTTGCAACCCCATCATCACCAAGCTGTACCAGAGTGCA 1842

Rattus_HSC71_NM_024351 CAGCAGAAAGAACTGGAGAAGGTCTGCAACCCTATCATCACCAAGCTGTACCAGAGTGCT 1842

Pelodiscus_HSC70_HQ219723 CAGCAGAAGGAGCTGGAGAAGGTCTGCAATCCCATAATCACAAAGCTGTACCAGAGCGCT 1842

Bos_HSP70_U09861 AAGAGGAAGGAGCTGGAGCAGGTGTGTAACCCCATCATCAGCAGACTGTACCAGGGGGCG 1842

Human_HSPA1A_NM_005345 AAGAGGAAGGAGCTGGAGCAGGTGTGTAACCCCATCATCAGCGGACTGTACCAGGGTGCC 1842

Rattus_HSP70_1A_NM_031971 AAGCGGGAGGAGCTGGAGCGGGTGTGCAACCCGATCATCAGCGGGCTGTATCAGGGTGCG 1842

X_maculatus_HSP70-1_AB062113 CAGCAGAATGAGCTGGAGAGGGTGTGCAAACCGGTCGTGGCAAAGTTGTATCAAGGTGCA 1848

X_maculatus_HSP70-2_AB062114 CAGCAGAAAGAGCTGGAGAAGGTGTGCAACCCCATCATCAGCAACTTGTACCAGGGAGGG 1848

Human_HSPA1L_NM_005527 AAGAGAAAGGAATTGGAGCAGATGTGTAACCCTATCATCACAAAACTCTACCAAGGAGGA 1848

Human_HSPA6_NP_002146 CAGAAGAGGGAGCTGGAGCAAATCTGTCGCCCCATCTTCTCCAGGCTCTATGGGGGGCCT 1848

Alligator_HSP70_AB306279 AAGCAGAAGGAGCTGGAGAAGATTTGCAACCCCATCATCACCAAGCTATACCAAGGCGGC 1851

** * ** * * *

Drosophila_Bb_AF295957 GCGGGAGCAGCTGGGGGTCCGGGA---GCCAACTGTGGCCAACAGGCCGGAGGATTTGGC 1890

Drosophila_Aa_AF295933 GCGGGAGCAGCTGGGGGTCCGGGA---GCCAACTGTGGCCAACAGGCCGGAGGATTTGGC 1890

Spodoptera_FJ754276 GCGCAAGGTGCGGGCGGGATGCCCGGAGGTATGCCTGGTGGCATGCCCGGAGGAATGCCT 1890

Bombyx_AB035326 CCCGGCGGTATG---------CCAGGAGGAATGCCGGGCGGTATGCCGGGAGGATACCAA 1881

Paralvinella_EF580992 TCTGGATCTTGTGGTGGTTCCGGATCACAACAAGGTTGTTACGGATCACAGACTGG---A 1905

B_plicatilis_HSP70-1_AB775784 GGTGGTGCGCCTGGTGGCATGCCTGGTGGTATGC------------CTGGCGGTATGCCT 1890

B_plicatilis_HSP70-2_AB775785 GGTGGTGCGCCTGGTGGTATGCCTGGTGGTATGCCTGGTGGTATGCCGGGCGGTATGCCT 1902

A_brightwelli_HSP70_KX119429 GCGCCGGGCGGTATGCCGGGTGGTATGCCGGGCGGTGCTGGTGC---------------T 1884

Wuchereria_AF167352 GGTGGTATGCCTGGAGGGATGCCGGGTGGCATGCCTAGTGGAGCTCCAGGTGCTGGTTCA 1902

Setaria_AF079360 GGTGGTATGCCCGGAGGAATGCCTGGTGGTATGCCTGGTGGAGCTCCAGGTGGAGGTTCG 1902

C_elegans_HSP70A_M18540 GGAGGAGCCCCACCAGGTGCTGCTCCAGGAGGAGCCGCCGGAGGAGC------------- 1892

C_elegans_HSP-1_NM_070667 GGAGGAGCCCCACCAGGTGCTGCTCCAGGAGGAGCCGCCGGAGGAGC------------- 1892

M_nipponense_KC460343 GGTGGTGCTCCTCCAGGTGGTATGCCAGGAGGTTTTCCAGGTGCCCCAGGGGCTGGTGCT 1902

M_nipponense_DQ660140 GGTGGTGCTCCTCCAGGTGGTATGCCAGGAGGTTTTCCAGGTGCCCCAGGGGCTGGTGCT 1902

Mytilus_HSP70_AY861684 GGTGGAGCCCCAGGTGGTGGTATGCCAAACTTTGGTGGAGCTGGTGGAGCCCCAGGAGGT 1905

Perinereis_KU255783 GGCGCCCCTGCTGGTGGAATGCCCGGTGGAATGCCCGGTGGAATGCCCGGAGGCTTCCCA 1899

Paralvinella_HSP70-2_EF580993 GGTGCTCCACCAGGCAGTATGCCAGGTGGCATGCCCGGTGGTATGCCAGGAGGCGCTGGT 1902

Haliotis_FJ812176 GGCGGTGCTGGTGGTATGCCCAACTTCAACCCAGGTGCTGCTGGTGCTGGCGGTGCAGGA 1902

Oncorhynchus_HSC71_AAB21658 GGTGGGATGCCCGGCGGTATGCCTGAGGGCATGGCTGGCGGATTCCCTGGAGCTGGTGGT 1902

X_maculatus_HSC70_AB062115 GGACAGATGCCTAGAGCTGGTGCGACCGGAACAGGAGGGGCCTCGGGCCCCACCA----- 1897

Human_HSPA8_HSC71_NP_006588 GGAGGCATGCCAGGAGGAATGCCTGGGGGATTTCCTGGTGGTGGAGCTCCTCCCTCTG-- 1900

Rattus_HSC71_NM_024351 GGTGGCATGCCTGGAGGAATGCCTGGTGGCTTCCCTGGTGGAGGAGCTCCTCCATCTG-- 1900

Pelodiscus_HSC70_HQ219723 GGAGGAATGCCTGGTGGCATGCCTGGGGGATTCCCTGGAGGTGGAGCTGCCCCATCTG-- 1900

Bos_HSP70_U09861 GGCGGCCCCGGGGCTGGCGGCTTTGGGGCTCAGGGCCCTAAAGGGGGC---TCTGGGTCT 1899

Human_HSPA1A_NM_005345 GGTGGTCCCGGGCCTGGGGGCTTCGGGGCTCAGGGTCCCAAGGGAGGG---TCTGGGTCA 1899

Rattus_HSP70_1A_NM_031971 GGTGCTCCCGGGGCTGGGGGCTTCGGGGCCCAGGCGCCCAAGGGAGGC---TCTGGGTCG 1899

X_maculatus_HSP70-1_AB062113 GCGGCAGGAAGCTCCAGCAGTCAGGCTGGAGGAGCCACTC------------------AG 1890

X_maculatus_HSP70-2_AB062114 AGGCCCACTGGGAGCTGCAGAGAGCAGGCAGGAGCCCACA---------------GCCAG 1893

Human_HSPA1L_NM_005527 TGCACTGGGCCTGCCTGCGGAACAGGGTATGTGCCTG------GAAGG---CCTGCCACA 1899

Human_HSPA6_NP_002146 GGTGTCCCTGGGGGCAGCAGTTGTGGCACTCAAGCCCGCCAGGGGGAC---CCCAGCACC 1905

Alligator_HSP70_AB306279 GCCCCGCCCGGCGGCCCCGGTCCCGCGTCCGCCGGCT------------------CCGGC 1893

Drosophila_Bb_AF295957 GG------CTACTCTGGACCCACAGTCGAGGAGGTCGACTAA------------------ 1926

Drosophila_Aa_AF295933 GG------CTACTCTGGACCCACAGTCGAGGAGGTCGACTAA------------------ 1926

Spodoptera_FJ754276 GGAGGAATGCCGGGCGGAATGCCTGGTGGCATGCCCGGCGGTATGCCAGGAGGAATGCCT 1950

Bombyx_AB035326 CAAGCGAGGAGTGATGGACCCACAGTTGAGGAAGTCGACTAA------------------ 1923

Paralvinella_EF580992 TATGGAAGT--------------------------------------------------- 1914

B_plicatilis_HSP70-1_AB775784 GGTGGAATG---CCTGGTGCTGGACCTGAGAGTGC------------------------- 1922

B_plicatilis_HSP70-2_AB775785 GGTGGAATG---CCTGGTGCTGGACCTGAGAGCGC------------------------- 1934

A_brightwelli_HSP70_KX119429 GGTGGCGCC---TCCGGAGCCGGA------------------------------------ 1905

Wuchereria_AF167352 ACGGGAGGTGGACC---------------------------------------------- 1916

Setaria_AF079360 GGTGGCAGTGGACC---------------------------------------------- 1916

C_elegans_HSP70A_M18540 -----TGGAGGACC---------------------------------------------- 1901

C_elegans_HSP-1_NM_070667 -----TGGAGGACC---------------------------------------------- 1901

M_nipponense_KC460343 GCTCCTG------GT------------------GG------------------------- 1913

M_nipponense_DQ660140 GCTCCTG------GT------------------GG------------------------- 1913

Mytilus_HSP70_AY861684 GCACCTGGAG---CA---------GGAGGCACTGG------------------------- 1928

Perinereis_KU255783 GGTGGCGCTGCTCCCGGTGGAGACTCCGGTGCTGC------------------------- 1934

Paralvinella_HSP70-2_EF580993 GGTGCTGGCGGGGCAGCTGG------------TTC------------------------- 1925

Haliotis_FJ812176 GGAGCTC---AGACAGG---------------CGG------------------------- 1919

Oncorhynchus_HSC71_AAB21658 GCTGCTCCTGGAGGTGGTGGATC------------------------------------- 1925

X_maculatus_HSC70_AB062115 ------------------------------------------------------------ 1897

Human_HSPA8_HSC71_NP_006588 -------------GTGGTGCTTC------------------------------------- 1910

Rattus_HSC71_NM_024351 -------------GTGGTGCTTC------------------------------------- 1910

Pelodiscus_HSC70_HQ219723 -------------GAGGGGCCTC------------------------------------- 1910

Bos_HSP70_U09861 GGC--------------------------------------------------------- 1902

Human_HSPA1A_NM_005345 GGC--------------------------------------------------------- 1902

Rattus_HSP70_1A_NM_031971 GGG--------------------------------------------------------- 1902

X_maculatus_HSP70-1_AB062113 GGT--------------------------------------------------------- 1893

X_maculatus_HSP70-2_AB062114 GGG--------------------------------------------------------- 1896

Human_HSPA1L_NM_005527 GGC--------------------------------------------------------- 1902

Human_HSPA6_NP_002146 GGC--------------------------------------------------------- 1908

Alligator_HSP70_AB306279 GGC--------------------------------------------------------- 1896

Drosophila_Bb_AF295957 ------------------------------------------------------- 1926

Drosophila_Aa_AF295933 ------------------------------------------------------- 1926

Spodoptera_FJ754276 GGCGGGTACGGAGGAAACCACAACAGCGGACCCACCATTGAAGAAGTTGATTAA- 2004

Bombyx_AB035326 ------------------------------------------------------- 1923

Paralvinella_EF580992 ---------------------CAACAAGGACCAACAGTGGAAGAGGTGGATTAA- 1947

B_plicatilis_HSP70-1_AB775784 -----------------GGGACGCAGTGGACCTACCATTGATGAGGTCGATTAA- 1959

B_plicatilis_HSP70-2_AB775785 -----------------GGGACGCAGTGGACCTACCATTGATGAGGTCGATTAA- 1971

A_brightwelli_HSP70_KX119429 ------------------------------CCTAAAATCGAAGAGGTCGACTAA- 1929

Wuchereria_AF167352 --------------------------------AACAATTGAAGAAGTTGATTAG- 1938

Setaria_AF079360 --------------------------------AACAATTGAAGAAGTTGATTAG- 1938

C_elegans_HSP70A_M18540 --------------------------------AACGATCGAGGAGGTCGACTAA- 1923

C_elegans_HSP-1_NM_070667 --------------------------------AACGATCGAGGAGGTCGACTAA- 1923

M_nipponense_KC460343 -----------------TGGTTCATCAGGCCCCACAATTGAAGAGGTCGATTAA- 1950

M_nipponense_DQ660140 -----------------TGGTTCATCAGGCCCCACAATTGAAGAGGTCGATTAA- 1950

Mytilus_HSP70_AY861684 -----------------TGGAAGTGGTGGACCAACCATTGAAGAGGTCGACTAA- 1965

Perinereis_KU255783 -----------------CCCAGGTGGTGGCCCCACAATCGAAGAGGTCGACTAA- 1971

Paralvinella_HSP70-2_EF580993 -----------------CGGTGGCAGTGGACCCACCATTGAGGAGGTTGATTAAs 1963

Haliotis_FJ812176 -----------------CAGCTCCGGAGGCCCCACCATCGAGGAGGTTGATTAA- 1956

Oncorhynchus_HSC71_AAB21658 -----------------------ATCTGGACCAACCATTGAGGAAGTCGACTAA- 1956

X_maculatus_HSC70_AB062115 -------------------------------------TAGAGGAGGTCGACTAA- 1914

Human_HSPA8_HSC71_NP_006588 -----------------------CTCAGGGCCCACCATTGAAGAGGTTGATTAA- 1941

Rattus_HSC71_NM_024351 -----------------------TTCAGGCCCCACCATTGAAGAGGTCGATTAA- 1941

Pelodiscus_HSC70_HQ219723 -----------------------ATCTGGACCAACCATTGAGGAAGTAGATTAA- 1941

Bos_HSP70_U09861 ------------------------------CCCACCATTGAGGAGGTGGATTAG- 1926

Human_HSPA1A_NM_005345 ------------------------------CCCACCATTGAGGAGGTAGATTAG- 1926

Rattus_HSP70_1A_NM_031971 ------------------------------CCCACCATCGAGGAGGTGGATTAG- 1926

X_maculatus_HSP70-1_AB062113 ------------------------------CCCACTGTCGAGGAGGTCGACTAA- 1917

X_maculatus_HSP70-2_AB062114 ------------------------------CCCACTGTGGAGGAGGTGGACTGA- 1920

Human_HSPA1L_NM_005527 ------------------------------CCCACAATTGAAGAAGTAGATTAA- 1926

Human_HSPA6_NP_002146 ------------------------------CCCATCATTGAGGAGGTTGATTGA- 1932

Alligator_HSP70_AB306279 ------------------------------CCCACCATCGAGGAGGTGGACTAG- 1920

**Supplementary Fig. S4.** Alignment of HSP70 nucleotide sequences used for the calculation of synonymous and nonsynonymous substitution rates. Regions shown in bold were used for the calculation.

**Supplementary Fig. S5.** Synonymous and nonsynonymous substitution rates.

>Amillepora_cA1_XM_029354964

GTTGGAGTATTTCAACACGGCAAAGTCGAGATTATTGCTAACGATCAAGGCAATCGTACAACCCCTAGTTATGTTGCCTTCAACGACACAGAGAGGTTAATCGGAGACGCAGCCAAGAACCAAGCAGCTCTCAACCCAAGTCATACGATATTTGACGCAAAGAGACTCATCGGTAGGAAATTCGACGATCCCACTGTGCAGTCCGACCGAGAGAAATGGCCTTTCCAAGTCATCAATGAATATGGCAAACCAAAAATTCGCGTTCAAAGCAAGGGAGATTGGAAATGGCTTTCACCAGAAGAAGTCAGCTCAATGGTGCTCATTAAGATGAAAGAAACAGCTGAAGCTTACTTGGGACAGACTGTGAAGAATGCGGTGATTACAGTGCCTGCGTATTTTAACGATTCACAAAGACAGGCCACAAAAGACGCCGGTGTGATAGCGGGACTGAACGTCAAACGAATTATCAATGAGCCCACA

>Drosophila_cA1i_AF295933

GTGGGTGTCTACCAGCATGGCAAGGTGGAGATTATCGCCAACGACCAGGGCAACCGCACCACGCCGTCCTACGTGGCTTTCACAGATTCGGAACGCCTCATCGGCGATCCGGCTAAGAACCAGGTGGCCATGAACCCCAGAAACACAGTGTTTGACGCCAAGCGACTCATCGGCCGAAAATACGACGACCCCAAGATCGCAGAGGACATGAAGCACTGGCCTTTCAAGGTGGTAAGCGACGGCGGAAAGCCCAAGATCGGGGTGGAGTATAAGGGTGAGTCCAAGAGATTTGCCCCCGAGGAGATCAGCTCGATGGTGCTGACCAAGATGAAGGAGACGGCGGAGGCATATCTGGGCGAGAGCATCACAGACGCAGTCATCACAGTTCCAGCCTACTTCAACGACTCCCAGCGCCAGGCTACCAAAGACGCCGGTCACATCGCCGGCCTGAATGTGCTCCGCATCATCAATGAGCCCACG

>Drosophila_cA2i_AF295957

GTGGGTGTCTACCAGCATGGCAAGGTTGAGATTATCGCCAATGACCAGGGCAACCGCACCACGCCGTCCTACGTGGCTTTCACAGACTCGGAACGCCTCATTGGTGATCCGGCCAAGAACCAGGTGGCCATGAACCCCAGAAACACAGTGTTTGACGCCAAGCGACTCATCGGCCGAAAATACGACGATCCCAAAATCGCAGAGGACATGAAGCACTGGCCTTTCAAAGTTGTAAGCGACGGCGGAAAGCCCAAGATCGGGGTGGAGTATAAGGGTGAGTCCAAGAGATTTGCTCCCGAGGAGATCAGTTCGATGGTGCTGACCAAGATGAAGGAGACGGCGGAGGCGTATCTGGGCGAGAGCATCACGGATGCAGTCATCACAGTTCCAGCTTACTTCAACGACTCTCAGCGCCAGGCTACCAAAGACGCCGGTCACATCGCCGGCCTGAATGTGCTCCGCATCATCAATGAGCCCACG

>Bombyx_cAli_AB035326

GTTGGAGTATGGCAGCACGGGAACGTGGAGATCATCGCGAACGACCAGGGCAACCGTACCACACCATCGTACGTCGCGTTCACGGACACGGAGCGTCTCATCGGCGACGCAGCCAAGAACCAGGTCGCCTTGAACCCTAACAACACCGTGTTCGACGCGAAGGGGCTGATCGGGAGGAAATTCGACGACCCCAAGATTCAGCAGGACATGAAGCACTGGCCCTTCAAAGTAATCAACGACTGCGGCAAACCGAAAATACAGATCGAGTTCAAAGGTGAGACGAAACGATTTGCGCCAGAAGAAATTAGCAGCATGGTGCTGACAAAAATGAAGGAGACGGCGGAAGCCTATCTGGGAAGTACAGTGCGGGATGCGGTAGTCACAGTTCCGGCATACTTCAACGACTCCCAGCGTCAGGCCACCAAGGACGCCGGAGCCATCGCCGGCCTGAACGTGCTTCGCATCATCAACGAGCCCACA

>Spodoptera_cA1i_FJ754276

GTCGGCGTGTGGCAGCACGGCAACGTGGAGATCATCGCCAACGACCAGGGCAACCGCACCACACCATCCTATGTGGCGTTCACGGACACGGAGCGCCTCATCGGAGACGCAGCCAAGAACCAGGTCGCCCTCAACCCCAACAACACTGTGTTCGACGCCAAGCGACTGATCGGAAGGAAATTCGATGACCCCAAGATCCAGGCAGACATGAAGCACTGGCCCTTCAGGGTGGTCAGCGACTGTGGCAAACCGAAGATCCAAGTGGAGTTCAAGGGTGAAACGAAACGGTTCGCGCCCGAGGAGATCAGCAGCATGGTGCTGACGAAGATGAAGGAGACGGCGGAAGCTTACCTCGGAACGACAGTACGCGACGCAGTGATCACAGTGCCGGCGTACTTCAACGACTCTCAGCGCCAGGCCACCAAGGACGCGGGAGCCATCGCCGGGCTGAACGTGCTCAGGATCATCAACGAGCCCACA

>Paralvinella_cA1i_EF580992

GTTGGTGTCTTCCAACATGGAAAAGTGGAGATTATTGCCAACGACCAAGGAAATAGGACGACACCAAGTTATGTGGCATTTACAGATACCGAGAGACTGATAGGTGATGCGGCAAAGAACCAAGTGGCGCTGAATCCGAGCAACACGGTATTTGATGCGAAGAGGCTGATTGGTCGAAGATTTGATGACGACAATGTCCAGAAGGACATCAAACATTGGCCTTTTAAAGTACTTAACGACGGTGGAAAGCCAAAGATTGACGTAGAATACAAAAGTGAGAGGAAACGTTTTACTCCAGAAGAAATCAGCTCGATGATACTTACCAAGATGAAAGAGACGGCCGAGGCCTATCTCGGTAATAAAGTGTTAGATGCCGTCATAACTGTCCCCGCTTACTTCAACGACTCTCAGAGACAAGCTACAAAAGATGCTGGTCTCATCTCCGGATTGAACGTTCTCCGAGTGATTAACGAGCCAACA

>Lingula_cA1_XM_013527000

GTTGGTGTATACCAGCATGGAAAGGTTGAGATCATCGCCAACGACCAGGGCAACCGAACAACACCGAGCTACGTTGCCTTCACAGACAGCGAGAGACTGATCGGCGATGCGGCTAAAAATCAAGTTGCCCTCAACCCACAGAATACAGTGTTCGACGCAAAGAGACTGATTGGTCGTAAATTTGATGATCCAGCGGTGCAGGCAGACAGCAAACACTGGCCTTTCAAGGTTGTCAATGAAGGTGGTAAACCCAAGGTGCAAGTTGAATACAAAGGTGAGACCAAGAAATGCACTCCTGAAGAAATAAGCTCTATGGTTCTCACTAAGATGAAGGAGACGGCAGAAGCCTACTTGGGTCAGAAGGTCAACGAAGCTGTCATCACTGTGCCAGCCTACTTTAATGACTCACAAAGACAGGCCACTAAAGATGCTGGTGCCATAGCAGGACTGAAGGTACTTCGTATTATCAACGAGCCAACA

>Aplysia_cA1_XM_005103777

GTGGGCGTATTCGAGCACGGAAAAGTGGAGATCATTGCAAACGATCAGGGCAACAGAACCACACCGAGCTACGTGGCTTTCACCGACAGTGAGAGGCTCATTGGTGATGCTGCCAAAAATCAAGCTGCGCTGAATCCGGCCAATACCGTGTTCGATGCCAAACGACTCATTGGGAGAAAGTTTGACGATAAGACAGTTCAGGATGACATGAAGCACTGGCCCTTCAAAGTGGTGGAAGTGGATCATCGGCCCAAAATTCAAGCAGAATACAAAGGTGAGAACAAATTGTTTGCTGCTGAGGAAGTGAGCTCCATGGTTCTGACAAAGATGAAGGAGACCGCGGAGGCGTTTTTGGGGAAGAAAGTGACCGATGCTGTCATCACGGTGCCTGCGTACTTCAACGACTCTCAGAGGCAAGCCACCAAGGATGCCGGCGCTATCGCTGGACTTAACGTGCTGCGCATAATTAATGAGCCCACT

>Aplysia_cA2_XM_005100297

GTGGGGATTTTTCAGCACGGCAAAGTGGAGATCATCGCTAACGACCAGGGCAACAGGACCACGCCCAGCTATGTGGCCTTCACAGACACCGAGAGACTGGTGGGAGACGCGGCCAAAAACCAGGCTGCTCTCAACCCCAGCAACACCATCTTTGACGCCAAGAGACTGATAGGTCGCAAGTTCACCGACAAGTCCGTTCAGTCGGACATGAAGCACTGGCCGTTCCAAGTGGCGGAGGTGGACAGCCGGCCAAAGATTGAGGCCGAGTACAAAGGAGAGAGGAAATTGTTTGCCCCTGAAGAAGTGAGCTCCATGGTTCTGAGCAAAATGAAGGAAACCGCCGAGGCGTACCTGGGCCAGAAAGTGACGGAGGCTGTGATCACAGTCCCCGCTTACTTCAACGACTCCCAGAGACAGGCCACCAAAGACGCCGGTGCCATTGCTGGCCTGAACGTTCTGAGAATGATCAATGAGCCCACA

>Crassostrea_cA1_JH818426

GTCGGTGTATTTCAACATGGAAAAGTGGAAATCATCGCCAACGACCAAGGAAACAGAACGACGCCCAGCTACGTCGCCTTCACAGACACAGAAAGACTGATAGGAGATGCGGCTAAAAACCAGGTAGCCATGAACGCCAACAATACAATCTTTGACGCCAAGAGGCTGATAGGCCGCAAGTTCAACGACGACAGTGTACAGTCCGACATGAAACATTGGCCGTTCACGGTGATCAATGATGGAGGAAAACCCAAGCTAGAAGTGGAATTCAAGAACGAGAAAAAGAGATTTACCCCCGAAGAAATCAGCTCAATGGTGCTGACCAAAATGAAGGAGACAGCAGAAGCTTACTTGGGACAAACTGTCCGAGACGCAGTCGTCACCGTTCCTGCCTACTTCAACAACGCCCAGAGAGAGGCCACTAAAGACGCCGGAGTGATAGCCGGTCTCAATGTTCTCAGGATAGTAAATGAACCCACA

>Lottia_cA1_XM_009053468

GTCGGAGTTTTCCAACATGGTAAAGTAGAGATCATCGCCAACGACCAAGGAAACAGAACGACACCAAGTTATGTGGCGTTTACAGATTCTGAGCGATTGATCGGAGACGCAGCGAAGAACCAAGTAGCATTAAATCCACAGAACACCATATTCGATGCAAAACGTCTGATTGGAAGAAAATTCACCGATGATACGGTCCAGAAGGACATGAAACACTGGCCGTTTACAGTGATCAATGATGGCGGCAAACCGAAATTCCAGGTGGACTACAAAAATGAAAAGAAAGTATTTCCACCCGAGGAAATCAGCTCCATGGTTTTAACGAAAATGAAGGAGACAGCTGAAGCCTATTTAGGACATAAAGTAAACGACGCAGTCGTCACAGTCCCAGCTTATTTCAACGATTCACAAAGATTAGCCACCAAAGACGCTGGGACTATTGCTGGATTAAACGTTCTGAGAATCATCAACGAACCAACA

>Lottia_cA3_XM_009053469

GTCGGAGTTTTCCAACATGGCAAAGTTGAGATCATTGCCAACGACCAAGGAAACAGAACGACACCAAGTTATGTGGCGTTTACAGATTCTGAGCGATTGATCGGAGACGCAGCGAAGAACCAAGTAGCATTGAATCCAGAAAACACCATATTCGATGCAAAACGTCTGATTGGAAGAAAATACACCGATGATACGGTCCAGAAGGACATGAAACACTGGCCGTTTACAGTGATCAATGATGGCGGCAAACCGAAATTCCAAGTGGACTACAAAAATGAAAAGAAAGTATTTCCACCGGAAGAAATTAGCTCCATGGTTTTAACGAAAATGAAGGAGACAGCTGAAGCGTATTTAGGTCAGAAGGTAAAGGACGCAGTCATCACAGTCCCAGCTTATTTCAACGATTCACAAAGATTGGCCACAAAAGACGCTGGGACTATTGCTGGATTAAACGTTCTGAGAATCATCAATGAACCAACA

>Lottia_cA2_XM_009047345

GTCGGAGTGTTTCAACATGGAAAAGTCGAGATTATCGCCAACGATCAAGGCAACAGAACGACACCAAGTTACGTTGCGTTTACAGACTCTGAACGTTTGATCGGAGACGCAGCTAAAAATCAAGTTGCCTTGAATCCACACAATACAATTTTTGATGCGAAACGTTTGATCGGAAGGAAGTTTACAGATGATACGGTGCAGAAGGACATGAAACATTGGCCGTTTACGGTCATCAACGATGGCGAAAAACCAAAGTTCCAGGTCGAGTACAAAAATGAGAAGAAAGTGTTTCCTCCAGAGGAAATCAGCTCCATGGTTTTGACAAAAATGAAGGAAACAGCCGAAGCTTACCTTGGCGAGAAAATCACTAACGCAGTTATTACCGTTCCGGCCTACTTCAACGATTCACAAAGATTAGCGACCAAAGATGCTGGTACGATCGCCGGATTAAATGTGTTGCGGATCATCAACGAACCGACA

>Lottia_cA4_XM_009058212

GTGGGTGTGTTCCAACATGGAGCAGTTGAAATTATAGCCAACGATCAAGGAAACAGAACCACGCCAAGTTATGTGGCTTTTACAGATTCGGAAAGATTAATTGGTGACAGCGCGAAAAACCAAGTGGCTTTAAATCCATCCAACACAATATTTGATGCCAAGCGTCTGATCGGAAGAAAGTTTGATGATGCAACAATTCAGTCTGATATGAAACACTGGCCATTTAAAGTGGTGAAGGACGGAGACAAGCCAAAGTTACAAGCGGAATTCAAGAACGAATTGAAGACATTTTCACCAGAAGAAGTTAGTGCTATGGTATTGACTAAAATGAAAGAGACAGCTGAAGCCTATCTTGGTGAGAATGTTACAAATGCTGTAATTACGGTACCAGCTTATTTTACTGATAGTCAAAGATCAGCAACTAAGGACGCTGGGGTGATCGCTGGTTTAAACGTCTTGAGAATCATTAATGAACCTACT

>Priapulus_cA1_XM_014826376

GTCGCCGTATTCCAGCACGGCAAGGCGGAGGTGATCGCTAACGAGCAGGGCAACCGCACGACGCCCAGCCACGTCGCTTTCACCGACAGCGAGCGTCTGATAGGCGACGCCGCCAAGCAGCAGGTGGCACGCAATCCGCACAACTCCGTGTTCGACGTGAAGCGACTCATCGGCCGACGCTACGACGACGCCGTCGTGCAGGCCGACGTCAAGCTGTGGCCGTTCGCCGTCGTCGGCCGCGACAACAAGCCGCACGTGCAGGTGCAGCACGCCGGCGAGACGAAGCTGTTCAGCGCCGAGGAGATCAGCTCGATGGTGCTCGGCAAGATGAAGGCGACCGCCGAGGCGTACCTGGGCAAGCAGGTAACCGACGCCGTCGTCACCGTGCCGGCCTACTTCAACGACTCGCAGCGGCAGGCGACGAAGGACGCCGGACGCATCGCCGGCCTCAACGTGCTGCGCATCGTCAACGAGCCGACG

>Nvectensis_cA1_XM_001636543

GTAGGGGTTTTCCAAAACGGCAAAGTGGAGATTATCGCCAACGATCAAGGTAACAGAACAACTCCGAGTTACGTTGGCTTCACCGACACCGAGCGATTAGTCGGCGATGCAGCGAAAAACCAAGTGGCTTTGAATCCAGAGAACACAATCTTCGACGCCAAGAGGCTGATAGGCCGCCGATATGACGATGCTACTGTGCAGTCTGACATGAAACTGTGGCCATTCAAGATTATCAGTGACAACAACAAACCGAAGATCCAAGTGGAGTACAAGGGCGAGCGCAAGACTTTTGCTGCCGAAGAAATCAGCTCCATGGTACTGGGCAAGATGAAGGAAACAGCCGAAGCCTACCTCGGACAAAAAGTCACCAGCGCAGTGATTACAGTCCCTGCATACTTTAACGACTCCCAGAGGCAAGCAACGAAAGATGCAGGGACCATTGCCGGACTGAACGTGTTGAGAGTAATTAATGAGCCGACT

>Schistosoma_cB1_L02415

GTTGGTGTTTTCCAGCATGGTAAAGTGGAGATAATTGCCAATGACCAGGGTAACAGAACGACACCGAGTTATGTGGCGTTCACAGACTCTGAGCGTTTAATTGGTGATGGAGCGAAGAACCAAGTGGCGATGAACCCAACAAATACAGTGTTTGATGCGAAGCGTCTAATCGGTCGTCGGTTCGATGATCCATCAGTGCAGAGTGATATGAAGCATTGGCCATTCGAGGTGACTCAAGTCGGTGGGAAGCTGAAGATTTGTGTTGAGTATAAGGGTGAGAAAAAGATGTTTTCCGCTGAGGAGATTTCGTCAATGGTGTTGACGAAGATGAAGGAGGTTGCTGAAAGTTATTTGGGCAGGACGGTGAGTGACGCTGTTATAACGGTTCCTGCTTACTTCAATGACAGTCAACGTCAAGCAACGAAAGATGCAGGTGCTATAGCTGGTCTTAATGTGTTGAGAATCATTAACGAGCCGACA

>Ofelineus_cB1_SJOL01002354_RC

GTAGGTGTATTTCAAAACAACAAGGTGGAAATCATTGCCAACGACCAAGGAAACCGCACAACTCCGAGTTATGTGGCCTTTACTGAAACCGAACGTTTGATTGGTGATGCGGCTAGGAACCAGGTTGCTATGAATCCAGCTAACACGGTCTTCGACGCCAAGCGCCTGATCGGTCGTAGGTATGATGACCCTACTGTCCAAGAGGATCGTAAAATGTGGCCATTCACGCTGATTGAAGAAAATGGCAAGCCGAAAATTCAGGTCGAATACAAAGGCCAAACGAAAACGTTCTTTGCTGAGGAGATATCCTCGATGGTATTGACAAAGATGAAGGAAATTGCAGAGGCCTATTTGGGAAAGACCGTCAGTGAGGCAGTCATCACTGTGCCGGCTTACTTCAATGATAGTCAACGGCAAGCCACGAAGGATGCAGGGACTATTGCCGGTCTAAACGTTCTACGGATTATCAATGAACCAACA

>Dugesia_cB1_EU380241

GTAGGAGTTTTTCAACATGGAAAAGTGGAAATAATTGCCAATGATCAAGGAAATAGAACAACTCCATCTTATGTTGCGTTTACTGATACAGAAAGATTGATAGGAGATGCAGCAAAGAATCAAGTTGCAATGAACCCGAGTAACACAGTGTTCGATGCTAAACGATTAATCGGAAGACGATTTGATGATGCAACTGTGCAAAGTGACATGAAACACTGGTCCTTTGATGTCGTTTGTGAAGGCGGCAGACCTAAAATTCAAGTCAATTATAAAGGTGAGTTGAAAAAATTTTTTCCAGAAGAAATCTCTTCAATGGTCTTATTGAAAATGAAAGAAACTGCCGAAGGATATTTGGGTAGACCTGTAACTGATGCAGTCGTAACAGTTCCTGCGTATTTCAATGACAGTCAACGTCAAGCAACAAAGGATGCCGGAGCAATTAGCGGTTTGAATGTGCTGAGGATCATTAACGAACCTACT

>Ofelineus_cB2_SJOL01002354

GTCGGGGTGTTCCAGCATGGAAAAGTGGAGATCATTGCCAACGACCAAGGAAACCGAACAACTCCGAGTTATGTAGCGTTTACCGACACCGAGCGCCTCATCGGTGATGCAGCTAAAAACCAGGTTGCAATGAATCCAACCAACACCGTTTTCGATGCCAAGCGATTGATCGGGCGCCGATTCGATGATGCTTCGGTGCAATCCGACATGAAACACTGGCCCTTCAATGTCGTTGACGACAAAGGCAAGCCGAAGATTCAGGTTGAGTACAGAGGCGAGACAAAGACATTCTCCCCCGAAGAGATATCGGCCATGGTTTTGGGGAAAATGAAGGAGATTGCCGAAGCCTACTTGGGCACCACGGTGAAGGATGCAGTCGTTACTGTGCCTGCCTACTTCAATGACAGTCAACGTCAAGCTACGAAAGATGCGGGTCGTATCGCCAACTTGAACGTTCAGCGGATTATCAACGAGCCGACG

>Ofelineus_cB3_SJOL01007550_RC

GTTGGAGTGTTCCAGCATGGAAAAGTGGAAATAATCGCCAACGATCAAGGAAATCGCACGACACCCAGCTATGTTGCATTTACGGATGCCGAACGACTGATTGGTGATGCCGCTAAAAATCAAGTCGCAATGAATCCAAACAACACCGTGTTTGATGTCAAGAGACTGATCGGTCGGCGATTCGATGATCCATCAGTGACAGCCGATCGTAAACACTGGCCTTTCGAAGTAGTCCAAGAAGGTGGTAAGCCAAAGGTTCGTGTGGAGTACAAAGGTGAGGTCAAGACGTTCTCGCCAGAGGAGATATCGTCTATGGTGTTGACGAAGATGAAGGAGACAGCCGAGGCGTATCTGGGCACGAAAGTGAAGGATGCTGTGGTTACTGTTCCGGCATATTTCAATGATAGTCAGCGTCAAGCGACGAAGGATGCGGGTGCCATTGCTGGCCTGAACGTTTTGCGTATCATCAACGAACCGACA

>Egranulosus_cB1_LK028702_RC

GTTGGTGTGTTTCAACACGGCAAGGTGGAGATAATCGCGAATGACCAAGGCAACCGCACGACGCCGAGTTATGTGGCGTTCACGGATACGGAGCGTCTGATTGGGGATGCGGCGAAGAACCAGGTGGCCATGAACCCGACGAATACAGTGTTCGATGCGAAGCGTCTGATCGGACGTCGTTTCGACGACAGGGCAGTGCAGGACGACATGAAGCACTGGCCGTTCAAGGTGATAAATGCGGGAGGCAAGCCGAAGATCGAGGTGGAGTACCGTGGTGAGACGAAGTGTTTCAGTGCGGAGGAGATATCATCGATGGTGCTGTTGAAGATGAAGGAGACGGCGGAGGCGTATTTGGGGAAGAAGGTGAGCGATGCAGTGATCACGGTGCCGGCGTACTTCAACGACAGTCAACGTCAGGCGACGAAGGATGCGGGTACAATATCGGGATTGAATGTGCTGCGGATAATCAATGAGCCGACG

>Egranulosus_cB2_XM_024498112

GTTGGTGTATTTCAACACGGCAAGGTGGAGATTATTGCCAATGATCAAGGCAATCGTACCACCCCGAGTTATGTCGCTTTTACGGATACGGAGCGTCTTATTGGTGATGCTGCCAAGAATCAGGTGGCTATGAATCCCAGCAACACGGTATTTGATGCCAAGCGATTGATAGGACGTCGTTTTGACGATAAGGCAGTGCAAGATGATATGAAGCATTGGCCTTTCAAGGTGATCAACTCAGGAGGCAAGCCAAAGATCGAAGTTGAGTATCGTGGTGAGACAAAGCAGTTCAGCGCAGAGGAGATCTCATCCATGGTACTGTCAAAGATGAAGGAAACTGCAGAAGCGTACTTGGGCAAGAAGGTATCAGATGCGGTTGTCACAGTGCCGGCTTACTTCAATGATAGTCAACGCCAGGCAACGAAGGATGCGGGTGCGATATCGGGTCTGAATGTATTACGTATTATCAACGAACCGACA

>Abrightwelli_cB1_KX119429

GTGGGCGTGTTCCAACATGGTAAAGTTGAGATCATTGCCAACGACCAAGGTAATAGAACAACGCCGAGTTACGTGGCGTTCACTGATACTGAACGTTTGATCGGAGACGCGGCCAAGAACCAAGTGGCCATGAATCCGACGAACACAGTGTTCGACGCGAAACGTCTCATCGGACGCAGATTCGACGACTCCACTGTCCAGTCCGACATGAAACACTGGCCGTTCACTGTCGTCAATGAAGGCGGAAAACCAAAAATCCAAGTCGAGTTCAAAGGCGAGAAAAAGACCTTCTTCCCTGAAGAAATCTCATCAATGGTCTTAATTAAAATGAAAGAGATCGCCGAGGCTTATTTGGGAAAGAAAGTAAACGACGCCGTGATCACAGTCCCGGCCTATTTCAACGACTCTCAAAGACAGGCCACAAAAGACGCCGGCGCCATTTCAGGTCTGAACGTGTTACGTATCATAAACGAGCCAACA

>Bplicatilis_cB1i_AB775784

GTGGGCGTATTCCAGCACGGCAAAGTGGAAATAATCGCCAACGACCAAGGCAACCGCACAACCCCCTCCTACGTAGCGTTCACCGACACCGAGCGCTTGATCGGCGACGCGGCCAAAAACCAAGTGGCCATGAACCCCAATAACACGGTGTTCGACGCCAAGCGTCTCATCGGCCGCAAGTTCGACGACCTGACCGTCCAGGCCGACATGAAGCACTGGCCCTTCACGGTGATCAGCGACAGCGGCCGGCCCAAAATCCAGGTCGAGTTCAAAGGCGAGACCAAGAGCTTCTACCCGGAAGAAGTCTCATCCATGGTACTGACCAAAATGAAGGAGACGGCCGAGGCCTACCTGGGCAAAAAGGTCACGGACGCAGTGGTGACCGTGCCCGCCTACTTCAACGACTCGCAGCGCCAAGCGACCAAGGACGCGGGCGCCATCGCCGGCCTCAACGTGCTGCGCATCATCAACGAGCCCACT

>Bplicatilis_cB2i_AB775785

GTGGGCGTATTCCAGCACGGCAAAGTGGAAATAATCGCCAACGACCAAGGCAACCGCACAACCCCCTCCTACGTAGCGTTCACCGACACCGAGCGCTTGATCGGCGACGCGGCCAAAAACCAAGTGGCCATGAACCCCAATAACACGGTGTTCGACGCCAAGCGTCTCATCGGCCGCAAGTTCGACGACCCGACCGTCCAGGCCGACATGAAGCACTGGCCCTTCACGGTGATCAGCGACAGCGGCCGGCCCAAAATCCAGGTCGAGTTCAAAGGCGAGACCAAGAGCTTCTACCCGGAAGAAGTCTCATCCATGGTACTGACCAAAATGAAGGAGACGGCCGAGGCCTACCTGGGCAAAAAGGTCACGGACGCAGTGGTGACCGTGCCCGCCTACTTCAACGACTCGCAGCGCCAAGCGACCAAGGACGCGGGCGCCATCGCCGGCCTCAACGTGCTGCGCATCATCAACGAGCCCACT

>Bmanjavacas_cB2_BmTi01_isotig03034_2025_165_2189_r_2_3

GTGGGCGTCTTCCAGCACGGCAAAGTGGAAATAATCGCCAACGACCAGGGCAACCGCACAACGCCCTCGTACGTCGCGTTCACCGACACCGAGCGCTTGATCGGCGACGCGGCCAAAAACCAAGTGGCCATGAACCCGAACAACACGGTGTTTGACGCCAAGCGGCTCATCGGCCGCAAGTTCGACGACCCGACCGTCCAGGCGGACATGAAGCACTGGCCCTTTACGGTGATCAGCGACGCGGGCCGCCCCAAGATCCAAGTGGAGTTCAAGGCCGAGACGAAGACGTTCTACCCGGAAGAAGTGTCGTCCATGGTGCTGACCAAGATGAAGGAGACGGCCGAGGCCTATCTGGGCAAAAAGGTGACGGACGCGGTGGTGACCGTGCCGGCCTACTTCAACGACTCGCAGCGCCAGGCGACCAAGGACGCGGGCGCCATCGCCGGCCTCAACGTGCTGCGCATCATCAACGAGCCGACC

>Bmanjavacas_cB1_BmTi01_isotig01884_1998_175_2172_f_0_5

GTGGGCATATTCCAGCACGGCAAAGTGGAAATAATCGCCAACGACCAGGGCAACCGCACAACACCCTCCTACGTGGCCTTCACCGACTCGGAGCGCTTGATCGGCGACGCGGCCAAAAACCAAGTGGCCATGAACCCGAACAACACGGTGTTCGACGCCAAGCGCCTCATCGGCCGCAAGTTCACGGACGCCGAGGTCCAGTCGGACATGAAGCACTGGCCGTTCACGGTGATCAGCGACGCGGGCCGCCCCAAGATCCAAGTGGAGTTCAAGGGCGAAACGAAGACTTTCTCGGCCGAGGAGGTCTCGTCCATGGTGCTGACCAAGATGAAGGAGACGGCCGAGGCCTATCTGGGCAAAAAGGTCACGGACGCAGTGGTGACCGTGCCGGCCTACTTCAACGACTCGCAGCGCCAGGCGACCAAGGACGCGGGCGCCATCGCCGGCCTCAACGTGCTGCGCATCATCAACGAGCCGACC

>Bcalyciflorus_cB2_NTPY01000792

GTTGGTGTATTTCAACATGGTAAAGTTGAAATCATTGCCAATGATCAAGGTAATCGTACAACACCATCATATGTTGCTTTTACAGATACTGAGCGTATGATTGGCGATGCTGCTAAGAATCAAGTAGCCATGAATCCAAACAATACTGTATTTGATGCCAAACGTTTAATCGGTCGTAAATTTGACGATCCGACTGTTCAAGCCGATATGAAACATTGGCCATTTACAGTTATCAATGAAAATGGTAGACCTAAAATTCAAGTTGAATTTAAAGGCGAGGTTAAGACTTTTTATCCAGAGGAAATTTCTTCAATGGTCTTGACCAAAATGAAAGAAACAGCTGAGGCTTATTTGGGTAAAAAAGTAAATGATGCTGTTGTTACAGTACCAGCTTATTTCAATGATTCACAACGTCAAGCTACCAAAGACGCTGGTGCCATTGCTGGTCTTAATGTCTTACGTATTATTAACGAACCAACT

>Bcalyciflorus_cB3_NTPY01000899

GTCGGTGTATTTCAACACGGTAAAGTTGAAATCATTGCCAATGATCAAGGTAATCGTACAACACCATCATATGTTGCTTTCACCGATAGTGAACGTTTAATTGGTGATTCGGCTAAGAATCAAGTAGCCATGAATCCAAACAATACTGTTTTTGATGCCAAGCGTTTAATCGGTCGTAAATTTACCGATACCGAAGTTCAATCTGATATGAAACACTGGCCTTTTACAGTCATTAATGAAAATTCAAAACCAAAAATTCAAGTTGAATTTAAAGGTGAGACTAAGACTTTTAATGCCGAGGAAATTTCATCAATGGTCTTGACCAAAATGAAAGACACAGCTGAGGCTTATTTGGGCAAAAAAGTAACTGATGCCGTCGTTACTGTACCAGCTTATTTCAATGATTCACAACGTCAAGCCACCAAGGACGCTGGTGCCATTGCTGGTCTTAATGTCTTACGTATTATTAACGAACCAACT

>Bcalyciflorus_cB1_NTPY01000620

GTTGGTGTTTTTCAACATGGTAAAGTTGAAATAATAGCTAATGATCAAGGCAATCGTACAACACCATCATATATTGCATTCACCGAAACAGAACGTTTAATTGGTGATGCTGCTAAAAATCAAGTCGCAATGAATCCTTTAAACACGGTTTTTGACGCCAAGCGTTTGATTGGTCGTAAATACGATGAGCCAGTGATTCAATCGGACATGAAACATTGGCCTTTTAAAGTAATAAACGATAATTCTAAGCCGAAAATTCAAGTCGATTTCAAAGGAGAAACAAAAACATTTTACCCAGAGGAAATTTCTTCAATGGTTTTGGTTAAAATGAAAGAAATAGCCGAGGCTTATTTAGGCAAAAAAGTAACTGATGCAGTTATAACTGTTCCTGCTTATTTCAATGACTCCCAACGTCAAGCAACTAAAGACGCAGGTGCAATAGCCGGTCTAAATGTGCTTAGAATAATTAATGAGCCAACT

>Amillepora_cB1_XM_029350702

GTTGGGGTTTTTCAGCATGGCAAGGTGGAAATCATCGCAAACGACCAAGGAAACCGTACGACACCCAGTTATGTGGCCTTCACCGAAAACGAAAGATTGATTGGGGATGCTGCAAAAAATCAGGTGGCTATGAATCCCACGAACACGATTTTTGACGCCAAGCGCCTGATCGGCCGAAGGTTTGACGATCGTACTGTACAAAGTGACATGAAACACTGGCCATTCGAGGTGATAAATGATTCAGGAAGACCAAAGATACGCGTGGAATATAAAGGAGAAAGAAAAACATTTTACGCTGAAGAGATTTCCTCAATGGTCTTAACAAAAATGAAAGAAACAGCAGAAGGTTACCTGGGGAAAACAATAACAGATGCTGTTATCACTGTACCTGCTTACTTTAATGATTCCCAGCGTCAGGCCACTAAAGATGCAGGTGTTATTGCCGGCTTGAATGTCTTGCGCATCATCAATGAACCAACG

>Aqueenslandica_cB1_XM_011405906

GTCGGTGTGTTCCAGCACGGGAAAGTAGAAATCATCCCCAACGACCAGGGGAACAGGACTACCCCTAGCTATGTCGCCTTCACTGACAAGGAGCGTCTCATTGGAGATGCAGCAAAGAACTGTATCACCATGAATTCGATTAATACGGTCTTTGGTGCCAAGCGACTCATTGGAAGGAAGTTCAACGATCCTAAAGTAACCGCTGACTGCAAGCACTGGCCATTTGCTGTCGTTAACACTGATGGCCGCCCTAAGATCTTAGTAGAATACTTGGGGAAGACAAAGACCTTCTACGCTGAAGAGGTGTCCTCTATGGTCCTCACTAAAATGAGGGAGACAGCTGAGGCTTACCTTGGGAAGACTGTCACTGATGTAGTCATTACAGTCCCTGCTTACTTCAATGACTCACAACGTCAGGCAACAAAAGACGCCGGAACCATAGCTGGTCTCAACGTCCTGCGTATCATCAACGAGCCCACA

>Aqueenslandica_cB2_XM_011405896

GTCGGTGTGTTCCAGCATGGGAAAGTAGAAATCATCCCCAACGACCGGGGGAACAGGACTACCCCTAGCTATGTCGCCTTCACTGACACAGAGTTTCTCGTTGGAGATGCAGCAAAGAACTGTATCACCATGAATTCGATTAACACGGTCTTTGGTGCCAAGCGACTCATTGGAAGGAAGTACAACGATCCTACAGTAGCCGCTGACCGCAAGCACTGGCCATTCTCTGTAGTTAACCTTGATGGCCACCCAAAGATCTTGGTAGACTACTTGGGGGAGACAAAGGCCTTCTACGCTGAAGAGATTTCCTCTGTGGTCCTCACTAAAATGAGGGAGACAGCTGAGGCTTACCTTGGGAAGACTGTCACTGATGTAGTCATTACAGTTCCTGCTTACTTCAATATTTCACAACGTCAAGCAACAAAAGATGCTGGAATTATTGCTGGTCTCAACGTCCTACGTATCATCAACGAGCCCACG

>Amillepora_cB2_XM_029339022

GTTGGCGTTTTCCAGCACGGCAAAGTCGAAATTATCGCAAATGATCAAGGAAACAGAACAACTCCCAGTTATGTTGCCTTCAATGATACGGAGAGACTCATCGGTGATTCAGCAAAAAATCAAGTCGCTCTCAATCCATCAAATACTGTTTTTGATGCGAAGCGTCTCATCGGGCGAAAGTTTGACGAAGAGTCTGTTCAATCAGATATGAAACATTGGCCTTTTAAGGTCGTAAGCGAAGGTGGGAGACCGAAGATTAGGGTGGACCACAAAGGAGAGACAAAATCTTTCTTTGCAGAGGAGATTTCTTCTATGGTTCTTACGAAAATGAAAGAAACTGCCGAGGCTTATCTGGGAGAGAAGGTCACCGACGCTGTTGTCACTGTGCCGGCATATTTCAACGATTCTCAGAGGCAAGCAACCAAAGATGCCGGCATCATTGCTGGACTTAATGTTCTTCGTATTATCAATGAACCCACC

>Nvectensis_cB1_XM_001622373

GTTGGAGTTTTTCAGCACGGCAAGGTCGAAATCATCGCAAACGACCAAGGAAATCGTACCACGCCAAGCTATGTTGCCTTCACGGATGAAGAGCGCTTGGTTGGCGATGCCGCGAAAAACCAAGTCGCAATGAATCCTACTAATACTATTTTCGACGCTAAGCGTTTGATCGGTCGTCGTTTCGATGATCCTGGGGTAAAGGACGATATGAAGCACTGGTCTTTCGAGGTAGTGAACGAGGCAGGTCGACCAAAAGTGAAAGTGGAGTACAAAGGAGAAACGAAAACCTTTTTCGCCGAGGAGATCTCTTCTATGGTTCTAAACAAGATGAAAGAGACAGCGGAAGCTTACCTCGGTTGCAAAGTGACAGACGCTGTTGTCACTGTCCCTGCGTACTTCAACGACTCCCAGAGACAGGCCACTAAAGATGCAGGTGTTATTTCTGGTTTGAATGTGCTCCGAATCATCAATGAGCCAACA

>Nvectensis_cB2_XM_001629293

GTAGGTGTTTTCCAACACGGAAAGGTCGAAATTATTGCGAACGATCAAGGAAATCGCACTACTCCAAGCTATGTAGCTTTCTCTGACACAGAGCGACTCATCGGCGATGCCGCTAAAAATCAGGTCGCAATGAACCCCGAGAACACAGTTTTTGATGCCAAGAGGCTGATCGGCCGAAAATTCGATGACCCCGTTGTTGCTCGTGATATGACCCATTGGCCGTTCCATGTCATTAGGGAAGGCGAGCGACCTAAAATTCAAGTCGAATTCAAAGGAGAAAAGAAAAGCTTCTTTCCCGAAGAGATTTCGTCCATGGTTTTGACCAAAATGAAGGAAACGGCAGAAGCTTATCTAGGGGCAAAAGTAACCGATGCCGTAGTCACAGTTCCGGCCTACTTCAACGATTCACAGCGTCAGGCTACCAAAGATGCAGGAACCATTGCTGGTCTCAATGTCCTCCGCATCATTAACGAGCCTACA

>Hvulgaris_cB1_NM_001309695

GTCGGAGTTTTCCAACACGGAAAAGTTGAAGTAATTGCTAACGAGCAAGGGAATCGCACCACTCCAAGTTACGTTGCATTTACCGACACAGAAAGATTAATTGGAGATGCAGCAAAAAATCAAGTTGCTATGAACCCTTCAAACACAGTGTTTGATGCGAAACGATTGATTGGTAGAAAATTTAACGATCCATCGGTCACTTCAGATAGAAAACATTGGCCATTTAATGTTATAGATGATGGATCCAGGCCAAAGATTCAAGTTGAGTTTAAAGGAGAAACGAAATCATTCTACCCAGAGGAGATATCCTCCATGGTGCTGCTAAAGATGAAGGAAATAGCGGATGCGTATCTTGGTAAAAAAGTCACAGATGTTGTCATAACTGTACCTGCTTACTTTAATGATTCTCAACGTCAAGCAACTAAAGATGCGGGAGTTATTGCTGGGTTGAATGTTTTGCGAATTATCAATGAACCGACT

>Trichuris_cB1_KL363195

GTTGGTGTGTTCCAGCATGGCAAGGTGGAGATAATAGCCAACGACCAGGGCAACCGCACCACGCCAAGCTATGTGGCTTTTACGGACACGGAGAGACTGATCGGCGATGCCGCCAAGAACCAAGTCGCCTTGAATCCTCACAACACCGTTTTCGACGCAAAGCGACTGATCGGGCGAAGATACGATGACGCTGCTGTGCAGTCTGACATGAAGCATTGGCCTTTCAAGGTGGTCAGTGACGGAGGCAAGCCAAAGATTCAGGTGGAATACAAAGGCGAGACCAAGATGTTTACTCCCGAAGAGGTCAGCGCTATGGTATTGGTTAAAATGAAAGAGACGGCAGAAGCTTATTTGGGTAAAACCGTGAAGGACGCCGTTATCACTGTTCCGGCCTACTTCAATGATTCTCAGAGGCAGGCAACGAAAGACGCAGGTACCATTTCTGGATTGAATGTCCTTCGAATCATCAATGAGCCGACC

>Daphnia_cB1_LRGB01002121

GTAGGTGTATTCCAGCATGGCAAAGTGGAGATCATTGCAAACGATCAGGGTAATCGTACAACACCATCTTACGTTGCGTTTACCGACACAGAACGTCTAATCGGAGATGCAGCCAAAAACCAAGTAGCCATGAATCCTATCAACACAGTTTTTGATGCAAAACGTTTGATTGGCCGTCGATTTGATGACGCCACTGTCCAGAGTGATATGAAGCATTGGCCATTCAAAGTTATAAGCGATGGAGGAAAACCGAAAATTCAAGTAGATTACAAAGGGGAAACGAAAACGTTTTCTCCCGAAGAGATCTCTTCTATGGTGCTTGTCAAGATGAAGGAGACGGCTGAAGCTTACCTAGGGCAAAAAGTCACTGATGCCGTGATTACTGTTCCTGCATATTTCAACGATTCCCAACGTCAAGCAACAAAGGATGCAGGAACCATCTCGGGATTGAACGTCCTTCGTATTATTAACGAGCCGACA

>Lingula_cB1_XM_013525464

GTAGGTGTGTTTCAACATGGCAAGGTGGAGATCATCGCTAACGACCAGGGTAATCGCACCACCCCCAGCTATGTGGCGTTCACCGACTCTGAGCGTCTGATTGGAGATGCTGCCATGAACCAGGTGGCGATGAACCCTAGTAACACTGTGTTTGATGCCAAGCGTCTCATTGGCCGAAAGTTTGACGACCCCTCTGTCGCCGCAGACATGAAGCATTTCTCCTTCAAGGTCATCAATGAGGGCGGCAAACCAAAGATCCAGGTGGAATACAAGGGAGAACAGAAAACTTTCTATCCTGAGGAGATCTCTTCCATGGTGTTGGTGAAGATGAAGGAGGTGGCTGAGGCTTATCTTGGCAAAAAAGTGACAGACTCCGTGATCACAGTTCCAGCTTACTTTAATGATTCCCAGCGCCAGGCCACCAAAGATGCCGGCACTATCTCTGGCATGAACGTGATGCGTATCATCAATGAGCCCACT

>Crassostrea_cB1_AB122064

GTTGGAGTTTTCCAGCATGGGAAGGTGGAAATCATCGCCAACGATCAGGGTAACCGAACCACCCCCAGTTATGTAGCGTTCACAGACACAGAAAGACTGGTCGGCGACGCAGCCAAAAACCAAGTCGCCATGAACCCCAACAACACAATTTTTGATGCCAAGCGTCTGATCGGCAGAAAATTCAACGATGCTTCAGTACAATCCGACATGAAACATTGGCCATTCACAGTGATCAATCAAGCAAGTAAACCCATGATCAAAGTCGAGTACAAAGGGGAAGAAAAGACCTTCTCTGCTGAGGAAGTCTCATCCATGGTCCTCAATAAAATGAAGGAAACTGCAGAAGCATATCTTGGCAAGACAATTAACAATGCCGTCGTCACAGTCCCAGCTTATTTCAATGATTCCCAGCGACAGGCTACCAAGGACGCTGGTACTATCTCAGGATTAAATGTACTACGTATCATCAACGAGCCAACA

>Mytilus_cB1i_AY861684

GTTGGAGTATTCCAGCATGGCAAAGTTGAAATCATTGCCAATGATCAAGGAAACAGAACAACCCCAAGCTATGTCGCCTTCACAGACACCGAAAGATTAATAGGTGATGCTGCCAAGAACCAAGTGGCAATGAACCCAGTCAACACGGTTTTTGATGCCAAGAGATTAATTGGTAGAAAGTTTGATGACGCTACAGTACAATCAGACATGAAGCATTGGCCTTTCACTGTTGTCAATGATGCCTCCAAACCTAAAGTTACGGTAGACTACAAAGGAGAAACAAAAACATTTTTCCCTGAGGAAATTTCATCAATGGTGCTGGTAAAAATGAAAGAAACTGCAGAAGCATATCTAGGAAAGTTAGTCAACAACAGTGTCATTACAGTCCCAGCTTATTTTAATGATTCACAAGGACAAGCAACAAAGGATGCTGGTACCATCTCTGGAATGAATGTTCTACGTATTATCAATGAGCCAACT

>Mytilus_cB2_AJ783714

GTTGGAGTATTCCAGCATGGCAAAGTTGAAATCATTGCCAATGATCAAGGAAACAGAACAACCCCAAGCTATGTCGCCTTCACAGACACCGAAAGATTAATAGGTGATGCTGCCAAGAACCAAGTGGCAATGAACCCAGTCAACACAGTTTTTGATGCCAAGAGATTAATTGGTAGAAAATTTGATGACGCTACAGTACAATCAGACATGAAGCATTGGCCTTTCACTGTTGTCAATGATGCCTCCAAACCTAAAATTACGGTAGACTACAAAGGAGAAACAAAAACATTTTTCCCTGAGGAAATTTCATCAATGGTGCTGGTAAAAATGAAAGAAACTGCAGAAGCATATCTAGGAAAGTTAGTCAACAACAGTGTCATTACAGTCCCAGCTTATTTTAATGATTCACAAAGACAAGCAACAAAGGATGCTGGTACCATCTCTGGAATGAATGTTCTACGTATTATCAATGAGCCAACT

>Daphnia_cB2_LRGB01001774

GTTGGTGTTTTCCAGCATGGAAAGGTAGAAATCATTGCTAATGACCAGGGTAATCGAACCACACCTTCTTACGTGGCATTTACCGACACCGAACGTCTTATCGGTGATGCTGCCAAGAACCAAGTCGCAATGAATCCTACCAATACAGTATTTGACGCTAAGCGATTGATCGGACGCAGATTCGAAGATTCTGTTGTCCAGGCTGACATGAAACATTGGCCATTTGAAGTAGTTAGTGATGGAGGGAAACCAAAGATCCAGGTTGATTACAAGAATGAAACCAAAACATTCTTCCCAGAAGAGATTTCCTCCATGGTGTTGCTCAAAATGAAAGAAACTGCCGAAGCTTACCTTGGCAAAACTGTTGGTAATGCTGTTGTAACCGTCCCTGCCTATTTTAATGATTCCCAGCGACAAGCCACTAAAGATGCTGGAACAATTGCTGGTCTAAATGTTCTTCGTATCATTAATGAGCCTACT

>Mnipponense_cB1i_KC460343

GTAGGTGTGTTCCAGCATGGAAAGGTAGAAATCATTGCCAATGATCAGGGCAACCGAACCACTCCATCATATGTTGCTTTCACCGATACAGAGCGTCTTATTGGTGATGCTGCCAAGAACCAAGTTGCTATGAATCCCAACAACACTGTATTTGATGCCAAGAGGCTCATTGGTCGTAAGTTTGAAGATCATGTAGTGCAGTCCGACATGAAACATTGGCCCTTTACAGTCATTAATGACAGCACGAAGCCCAAAATTCAAGTTGATTACAAAGGAGAGACCAAAACCTTTTTCCCAGAAGAGATCTCCTCAATGGTGCTTATTAAGATGAAAGAAACTGCAGAAGCCTTTTTGGGTGGTACTGTAAAGGATGCTGTCGTCACAGTCCCTGCCTATTTCAATGACTCCCAGCGTCAGGCCACCAAAGATGCTGGTACAATCTCTGGTCTCAATGTGCTCCGTATCATCAATGAGCCCACT

>Mnipponense_cB2i_DQ660140

GTAGGTGTGTTCCAGCATGGAAAGGTAGAAATCATTGCCAATGATCAGGGCAACCGAACCACTCCATCATATGTTGCTTTCACCGACACAGAGCGTCTTATTGGTGATGCTGCCAAGAACCAAGTTGCTATGAATCCCAACAACACTGTATTTGATGCCAAGAGGCTCATTGGTCGTAAGTTTGAAGATCATGTAGTGCAGTCCGACATGAAACATTGGCCCTTTACAGTCATTAATGACAGCACGAAGCCCAAAATTCAAGTTGATTACAAAGGAGAGACCAAAACCTTTTTCCCAGAAGAGATCTCCTCAATGGTGCTTATTAAGATGAAAGAAACTGCAGAAGCCTTTTTGGGTGGTACTGTAAAGGATGCTGTCGTCACAGTCCCTGCCTATTTCAATGACTCCCAGCGTCAGGCCACCAAAGATGCTGGTACAATCTCTGGTCTCAATGTGCTCCGTATCATCAATGAGCCCACT

>Priapulus_cB1_XM_014812613

GTCGGAGTATTTCAGCATGGCAAAGTTGAAATCATTTTCAACGATATGGGCCACAACACCACACCCAGCTATGTATCATTTACGGATGAAGAAACACTCATCGGTGATGCAGCAAAAACCCAGGCTGTAAAGAACCCGATCAACACTGTCTATGATGCTAAGCGATTGATTGGAAGGCGTTTCGAAGACACAACCGTCCAGCAGGATATGAAACACTGGCCTTTCACAGTCGTCAGCGATGCAGGCAAGCCCAAGATCCGCATAGACTACAAGGGTGAGAACAAACTGTTCTTCCCAGAAGAAGTCTCGTCCATGGTACTAAACAAGATGAAGGAAACCGCTGAGGCCTACCTCGGAAGGACTGTGGCAAACGCTGTAGTAACAGTACCAGCCTATTTCAATGACAGTCAGCGGCAGGCAACGAAAGACGCCGGCACCATTTCTGGTCTCAATGTCCTACGTATTATCAACGAACCAACA

>Priapulus_cB2_XM_014817626

GTGGGCGTATTCCAGCACGGCAAGGTGGAGATCATCGCCAACGATCAGGGAAATCGGACGACGCCCAGCTACGTCGCGTTCACCGACACCGAGCGATTGATCGGCGACGCCGCCAAGAACCAGGTGGCGCTCAACCCCAACAACACCGTGTTCGATGCGAAGCGTCTCATCGGCCGGCGATTCGACGACCCGACCGTGCAGATGGACATGAAGATGTGGCCTTTCCAGGTGGTGAGCGACGCGGGAAAGCCGCGGGTGCGTGTCGATTACAAGGGCGAGACGAAGACGTTCTATGCCGAGGAGATCTCCTCGATGGTTCTGACGAAGATGCGCGAGACCGCCGAGGCCTTCCTCGGCAAGAGCGTGACGGATGCTGTCGTCACGGTGCCGGCGTACTTCAATGACTCGCAGCGGCAGGCAACGAAAGATGCAGGGGTCATCGCCGGACTCAACGTCCTCCGCATCATCAACGAGCCGACG

>Limulus_cB1_XM_013923214

GTGGGAGTGTTCCAGCATGGAAAAGTAGAAATAATAGCTAATGATCAAGGAAACCGCACTACTCCCAGTTATGTAGCATTTACAGATACAGAAAGACTTATTGGGGATGCAGCTAAAAACCAGGTTGCAATGAACCCTAATAATACTGTATTTGATGCAAAACGCCTCATTGGCAGAAAGTGGGATGACCCAACAGTTGCACAGGACATGAAACACTGGCCATTTGAAGTAATCAATGATGGAGGAAAACCTAAGATCAGGGTAGAATACAAAGGGGAGGTCAAATCATTTTACCCTGAGGAAATTTCTTCTATGGTTTTAACTAAGATGAAGGAAATTGCAGAGGCATATTTAGGGAAGACAATCTCAAATGCTGTAATCACTGTACCTGCCTACTTCAATGACTCCCAGCGGCAGGCTACGAAGGATGCTGGAACCATTTCTGGAATGAATGTACTTCGTATAATTAATGAACCTACA

>Bos_cB2i_U09861

GTAGGGGTGTTCCAGCACGGCAAGGTGGAGATCATCGCCAACGACCAGGGCAACCGCACCACCCCCAGCTACGTGGCCTTCACCGATACCGAGCGGCTCATCGGCGATGCGGCCAAGAACCAGGTGGCGCTGAACCCGCAGAACACGGTGTTCGACGCGAAGCGGCTGATCGGCCGCAAGTTCGGAGACCCGGTGGTGCAGTCGGACATGAAGGAGTGGCCTTTCCGCGTCATCAACGACGGAGACAAGCCTAAGGTGCAGGTGAGCTACAAAGGGGAGACCAAGGCGTTCTACCCGGAGGAGATCTCGTCGATGGTGCTGACCAAGATGAAGGAGATCGCCGAGGCGTACCTGGGCCACCCGGTGACCAACGCGGTGATCACCGTGCCGGCCTACTTCAACGACTCGCAGCGGCAGGCCACCAAGGACGCGGGGGTGATCGCGGGGCTGAACGTGCTGAGGATCATCAACGAGCCCACG

>Human_cB2i_NM_005346

GTGGGGGTGTTCCAACACGGCAAGGTGGAGATCATCGCCAACGACCAGGGCAACCGCACCACCCCCAGCTACGTGGCCTTCACGGACACCGAGCGGCTCATCGGGGATGCGGCCAAGAACCAGGTGGCGCTGAACCCGCAGAACACCGTGTTTGACGCGAAGCGGCTGATTGGCCGCAAGTTCGGCGACCCGGTGGTGCAGTCGGACATGAAGCACTGGCCTTTCCAGGTGATCAACGACGGAGACAAGCCCAAGGTGCAGGTGAGCTACAAGGGGGAGACCAAGGCATTCTACCCCGAGGAGATCTCGTCCATGGTGCTGACCAAGATGAAGGAGATCGCCGAGGCGTACCTGGGCTACCCGGTGACCAACGCGGTGATCACCGTGCCGGCCTACTTCAACGACTCGCAGCGCCAGGCCACCAAGGATGCGGGTGTGATCGCGGGGCTCAACGTGCTGCGGATCATCAACGAGCCCACG

>Rat_cB2i_NM_031971

GTGGGCGTGTTCCAGCACGGCAAGGTGGAGATCATCGCCAACGACCAGGGCAACCGCACGACCCCCAGCTACGTGGCCTTCACCGACACCGAGCGGCTCATCGGGGACGCCGCCAAGAACCAGGTGGCGCTGAACCCGCAGAACACCGTGTTCGACGCGAAGCGGCTGATCGGCCGCAAGTTCGGCGACCCGGTGGTGCAGTCGGACATGAAGCACTGGCCCTTCCAGGTGGTGAACGACGGCGACAAGCCCAAGGTGCAGGTGAACTACAAGGGCGAGAACCGGTCGTTCTACCCGGAGGAGATCTCGTCCATGGTGCTGACCAAGATGAAGGAGATCGCCGAGGCGTACCTGGGCCACCCGGTGACCAACGCGGTGATCACCGTGCCCGCCTACTTCAACGACTCGCAGCGGCAGGCCACCAAGGACGCGGGCGTGATCGCGGGTCTGAACGTGCTGCGGATCATCAACGAGCCCACG

>Human_cB3c_NM_005527

GTGGGGGTGTTCCAGCACGGCAAGGTGGAGATCATCGCCAACGACCAGGGCAACCGCACCACCCCCAGCTACGTGGCCTTCACAGACACCGAGCGGCTCATTGGGGATGCGGCCAAGAACCAGGTAGCAATGAATCCCCAGAACACTGTTTTTGATGCTAAACGTCTGATCGGCAGGAAATTTAATGATCCTGTTGTACAAGCAGATATGAAACTTTGGCCTTTTCAAGTGATTAATGAAGGAGGCAAGCCCAAAGTCCTTGTGTCCTACAAAGGGGAGAATAAAGCTTTCTACCCTGAGGAAATCTCTTCGATGGTATTGACTAAGTTGAAGGAGACTGCTGAGGCCTTTTTGGGCCACCCTGTCACCAATGCAGTGATTACCGTGCCAGCCTATTTCAATGACTCTCAACGTCAGGCTACTAAGGATGCAGGTGTGATTGCTGGACTTAATGTGCTAAGAATCATCAATGAGCCCACG

>Human_cB5i_NM_002155

GTGGGCGTGTTTCAGCAGGGCCGCGTGGAGATCCTGGCCAACGACCAGGGCAACCGCACCACGCCCAGCTACGTGGCCTTCACCGACACCGAGCGGCTGGTCGGGGACGCGGCCAAGAGCCAGGCGGCCCTGAACCCCCACAACACCGTGTTCGATGCCAAGCGGCTGATCGGGCGCAAGTTCGCGGACACCACGGTGCAGTCGGACATGAAGCACTGGCCCTTCCGGGTGGTGAGCGAGGGCGGCAAGCCCAAGGTGCGCGTATGCTACCGCGGGGAGGACAAGACGTTCTACCCCGAGGAGATCTCGTCCATGGTGCTGAGCAAGATGAAGGAGACGGCCGAGGCGTACCTGGGCCAGCCCGTGAAGCACGCAGTGATCACCGTGCCCGCCTATTTCAATGACTCGCAGCGCCAGGCCACCAAGGACGCGGGGGCCATCGCGGGGCTCAACGTGTTGCGGATCATCAATGAGCCCACG

>Xmaculatus_cB1i_AB062113

GTCGGGGTCTTCCAGCATGGAAAAGTGGAAATTATCGCCAATGATCAAGGCAACAGGACAACTCCCAGCTACGTGGCCTTTACCGACACAGAGAGGCTCATCGGAGATGCTGCCAAGAACCAAGTTGCCATGAACCCCACCAACACGATCTTTGATGCCAAACGGCTCATTGGAAGAAGGTTTGATGACCTGGTGGTCCAGTCTGACATGAAACTCTGGCCGTTCAAGGTGATCAACGACAACGGAAAGCCCAAAGTCCAGGTGGAATATAAAGGAGAAATTAAGACATTCTGTCCTGAAGAGATTTCCTCGATGGTCCTGGTTAAAATGAGAGAAATAGCTGAGGCCTTTCTGGGACAGAGGGTGTCGAATGCGGTCATCACAGTGCCAGCTTACTTTAATGATTCCCAAAGGCAAGCCACAAAGGATGCTGGAGTGATCTCCGGACTAAATGTTCTCCGCATCATAAATGAGCCGACA

>Xmaculatus_cB2i_AB062114

GTGGGGGTTTTCCAGCACGGAAAAGTGGAAATCATCGCCAACGACCAGGGCAACAGGACCACCCCCAGCTACGTGGCCTTCACCGACACCGAGAGGCTGATCGGGGACGCGGCCAAGAACCAGGTGGCCCTGAACCCCAGCAACACGGTGTTTGACGCCAAGAGGCTGATCGGCAGAAAGTTTGAAGAGCCAGTGGTGCAGGCCGACATGAAGCACTGGCCCTTCGAGGTGCTTTCAGACGGAGGCAGGCCCAAAATTCAGGTGGAGTACAAAGGGGAGAACAAAGCCTTCTTCCCTGAGGAGATCTCCTCCATGGTCCTGGTGAAGATGAAGGAAATCGCCGAGGCCTACCTGGGCCACAAGGTGTCCAACGCCGTGATCACGGTCCCCGCCTATTTCAACGACTCCCAGCGACAGGCCACCAAAGACGCAGGCGTCATTGCGGGACTCAACGTCTTGAGGATCATCAACGAGCCCACG

>Human_cB4c_NM_021979

GTCGGGGTCTTCCAACATGGCAAGGTGGAGATCATCGCCAACGACCAGGGCAATCGCACCACCCCCAGCTACGTGGCCTTCACGGACACCGAGCGCCTCATCGGCGACGCCGCCAAGAACCAGGTGGCCATGAACCCCACCAACACCATCTTCGACGCCAAGAGGCTGATTGGACGGAAATTCGAGGATGCCACAGTGCAGTCGGATATGAAACACTGGCCGTTCCGGGTGGTGAGCGAGGGAGGCAAGCCCAAAGTGCAAGTAGAGTACAAGGGGGAGACCAAGACCTTCTTCCCAGAGGAGATATCCTCCATGGTCCTCACGAAGATGAAGGAGATCGCGGAAGCCTACCTGGGGGGCAAGGTGCACAGCGCGGTCATAACGGTCCCGGCCTATTTCAACGACTCGCAGCGCCAGGCCACCAAGGACGCAGGCACCATCACGGGGCTCAATGTGCTGCGCATCATCAACGAGCCCACG

>Alligator_cB1_AB306279

GTGGGCGTCTTCCAGCATGGCAAGGTGGAGATCATTGCCAACGACCAGGGCAACCGCACCACGCCCAGCTACGTGGCCTTCACGGACACGGAGCGCCTCATCGGCGACGCGGCCAAGAACCAGGTGGCCATGAACCCCACCAACACCATCTTCGACGCCAAGCGCCTCATCGGCCGCAAGTACGACGACCCCACGGTGCAGGCGGACATGAAGCACTGGCCCTTCCACGTAGTGTCCGAGGGCGGCAAGCCCAAGGTGCAGGTGGAGTATAAGGGCGAGGCCAAGACCTTCTTCCCCGAAGAGATCAGCTCCATGGTGCTGACTAAGATGAAGGAGATCGCCGAAGCCTACCTGGGCCGCAAGGTGCAGAACGCCGTCATCGCGGTGCCCGCCTACTTCAACGACTCGCAGCGCCAGGCCACCAAGGACGCGGGCACCATCACGGGCCTCAACGTGATGCGCATCATCAACGAGCCCACG

>Oncorhynchus_cB1c_S85730

GTGGGTGTGTTCCAGCATGGCAAGGTTGAAATCATTGCCAACGACCAAGGCAACAGGACCACTCCAAGCTACGTTGCCTTCACTGACTCTGAGAGGCTCATCGGTGATGCTGCCAAGAATCAGGTTGCCATGAACCCCTGCAACACAGTATTCGATGCTAAGAGACTGATTGGCCGCAGGTTTGATGATGGAGTTGTTCAATCGGACATGAAGCATTGGCCCTTTGAAGTTATCAATGATTCTACTCGGCCTAAGCTCCAAGTTGAATACAAAGGAGAGACTAAGTCCTTCTACCCAGAAGAAATTTCATCTATGGTTCTGGTCAAGATGAAGGAGATTGCTGAGGCCTACCTTGGGAAAACTGTCAACAATGCTGTTGTTACCGTACCTGCCTACTTCAATGACTCCCAGCGCCAGGCAACCAAAGATGCTGGTACCATCTCGGGGCTGAATGTGCTGCGTATCATCAATGAGCCAACT

>Xmaculatus_cB3c_AB062115

GTGGGAGTGTTTCAGCACGGGAAAGTTGAGATCATTGCTAATGACCAGGGGAACAGGACCACGCCCAGTTATGTGGCCTTTACTGACACAGAGAGGCTGATTGGAGATGCAGCCAAGAACCAGGTGGCCCTCAACCCCAACAACACCGTTTTTGATGCAAAGCGGCTTATTGGGCGTCGTTTTGACGATAGTATTGTCCAATCAGACATGAAACATTGGCCCTTCACCATCATCAATGATAGCTCGAGGCCCAAAGTGAAAGTGGAGTACAAAGGGGAGACGAAGACCTTCTACCCAGAGGAGATCTCCTCCATGGTGCTGCTGAAGATGAAGGAGATTGCTGAGGCATACCTTGGCAAGACTATAACAAATGCCGTGGTGACTGTACCTGCCTACTTCAATGACTCTCAGCGTACGGCCACCAAAGACGCTGGGACCATTTCTGGGCTCAATGTTCTGCGTATCATTAACGAGCCAACG

>Human_cB6c_NM_006597

GTGGGTGTTTTCCAGCACGGAAAAGTCGAGATAATTGCCAATGATCAGGGAAACCGAACCACTCCAAGCTATGTCGCCTTTACGGACACTGAACGGTTGATCGGTGATGCCGCAAAGAATCAAGTTGCAATGAACCCCACCAACACAGTTTTTGATGCCAAACGTCTGATTGGACGCAGATTTGATGATGCTGTTGTCCAGTCTGATATGAAACATTGGCCCTTTATGGTGGTGAATGATGCTGGCAGGCCCAAGGTCCAAGTAGAATACAAGGGAGAGACCAAAAGCTTCTATCCAGAGGAGGTGTCTTCTATGGTTCTGACAAAGATGAAGGAAATTGCAGAAGCCTACCTTGGGAAGACTGTTACCAATGCTGTGGTCACAGTGCCAGCTTACTTTAATGACTCTCAGCGTCAGGCTACCAAAGATGCTGGAACTATTGCTGGTCTCAATGTACTTAGAATTATTAATGAGCCAACT

>Bos_cB1_NM_174345

GTGGGTGTCTTCCAGCACGGAAAGGTGGAAATAATTGCCAATGATCAGGGGAACCGAACCACCCCAAGCTATGTCGCCTTTACTGATACCGAACGGTTAATCGGTGATGCAGCAAAGAACCAAGTCGCAATGAATCCCACCAACACGGTTTTTGATGCCAAACGACTTATTGGACGAAGATTTGATGATGCTGTTGTCCAGTCTGATATGAAACATTGGCCCTTCATGGTGGTGAATGATGCTGGCAGGCCTAAGGTTCAAGTAGAATACAAGGGAGAGACAAAGAGTTTTTACCCAGAGGAGGTGTCATCCATGGTCCTGACAAAGATGAAGGAAATCGCAGAAGCCTACCTTGGGAAGACGGTTACCAACGCTGTCGTCACAGTACCTGCCTATTTTAATGACTCTCAGCGTCAGGCTACCAAAGATGCTGGAACTATTGCTGGTCTCAACGTACTTCGAATCATCAATGAGCCAACT

>Rat_cB1c_NM_024351

GTGGGTGTCTTCCAGCATGGAAAGGTGGAAATAATTGCCAATGACCAGGGTAACCGCACCACGCCGAGCTATGTTGCTTTCACCGACACAGAACGATTAATTGGGGATGCGGCCAAGAATCAGGTTGCAATGAACCCCACCAACACAGTTTTTGATGCCAAACGTCTGATCGGACGTAGGTTCGATGATGCTGTTGTTCAGTCTGACATGAAGCACTGGCCCTTCATGGTGGTGAACGATGCAGGCAGGCCCAAGGTCCAAGTCGAATACAAAGGGGAGACAAAAAGTTTCTATCCTGAGGAAGTGTCTTCAATGGTTCTGACAAAAATGAAGGAAATTGCAGAAGCTTACCTTGGAAAGACTGTTACCAATGCCGTGGTCACCGTGCCAGCTTACTTCAATGACTCTCAGCGACAGGCAACAAAAGATGCTGGAACTATTGCTGGCCTCAACGTACTTCGAATTATCAATGAGCCAACT

>Pelodiscus_cB1i_HQ219723

GTTGGGGTCTTCCAACATGGCAAGGTGGAGATCATTGCCAATGATCAGGGCAACAGGACCACGCCAAGTTACGTTGCCTTCACAGATACAGAGAGGTTGATCGGTGATGCTGCAAAGAACCAAGTTGCAATGAATCCTACCAACACGGTTTTTGATGCAAAGCGGCTGATCGGTCGTAGATTTGATGATGCTGTTGTCCAGTCGGACATGAAACATTGGCCATTCACTGTGGTGAATGATGCTGGCAGGCCGAAAGTCCAGGTTGAGTACAAAGGGGAGACCAAGAGCTTCTATCCAGAGGAAATATCTTCCATGGTTTTGACAAAGATGAAAGAGATAGCAGAAGCATATCTTGGGAAGACTGTTACTAATGCAGTGGTCACGGTACCAGCCTACTTCAATGACTCCCAGCGCCAGGCCACAAAAGATGCTGGAACCATTGCAGGTCTTAATGTGCTCAGGATCATCAATGAACCAACT

>Tevnia_cB1_FN860147

GTGGGTGTCTTCCAGCACGGCAAGGTGGAGATCATCGCCAACGACCAGGGCAACCGTACGACGCCCAGCTATGTGGCGTTCACAGACTCTGAGCGTCTCATTGGAGACGCAGCCAAGAACCAAGTGGCCATGAATCCCAGCAACACGGTGTTCGATGCGAAGCGACTGATTGGGCGTCGCTTCGACGAACACGCCGTCCAGTCGGACATCAAGCACTGGCCGTTCCACGTCGTAAACGAGAGCGGCAAGCCGAAGATCCACGTGGAATACAAGGGAGAGAAGAAGTTGTTCTTCCCAGAAGAAATATCGTCCATGGTACTCAACAAGATGAAAGAAACGGCCGAAGCTTACCTTGGCATGACTGTGACCGACGCGGTGGTGACAGTACCTGCCTACTTCAACGACTCTCAGCGTCAGGCGACCAAGGATGCCGGAACCATCTCAGGCCTCAACGTCCTACGAATCATCAACGAGCCGACG

>Tevnia_cB2_FN860148

GTGGGCGTCTTCCAGCACGGCAAGGTGGAGATCATTGCCAACGACCGGGGCAACCGGACGACGCCCAGCTATGTGGCCTTCACAGACTCCGAGCGTCTCATCGGAGACGCCGCCAAGAACCAGGTGGCCATGAACCCCAGCAACACAGTGTTTGATGCCAAGCGACTGATCGGACGCCGCTTCGACGAGCAGGCCGTCCAGTCGGACATCAAGCACTGGCCGTTTGAAGTTACCAACGAGACCGGCAAGCCAAAGATCCGTGTAGAATACAAGGGAGAGCAGAAGTTGTTCTTCCCCGAGGAAATCTCGTCCATGGTTCTGAACAAGATGAAGGAAACGGCAGAAGCCTACCTCGGCATGACTGTGAAGGACGCGGTGGTGACAGTGCCCGCCTACTTCAACGACTCCCAGCGCCAGGCGACCAAGGACGCCGGAACCATCTCGGGCCTCAACGTCCTGCGAATCATCAACGAGCCGACG

>Perinereis_cB1i_KU255783

GTTGGGGTTTTCCAGCACGGGAAAGTGGAAATCATTGCAAACGACCAGGGTAACAGGACCACGCCCAGTTATGTGGCATTCACAGACTCCGAGCGTCTCATTGGAGATGCCGCAAAGAACCAAGTGGCCATGAACCCCGAGAACACAGTCTTCGACGCCAAACGTTTGATCGGACGCAAGTTTGACGACTCAGCCGTTCAGTCAGACAAAAAGCATTGGCCATTCGATGTGGTCAGTGAGGGTGGCAAACCAAAGATCTCTGTTGATTACAAGGGGGAGAAGAAATCATTTTACCCAGAAGAGATCTCCTCCATGGTATTGGTTAAGATGAAGGAAACAGCCGAGGCTTACATCGGCAAGACTGTTCTAAATGCCGTCGTAACAGTGCCTGCCTACTTCAACGATTCTCAGCGCCAAGCAACCAAAGATGCCGGAACTATCTCTGGCTTGAATGTATTACGTATCATCAATGAACCAACG

>Perinereis_cB2_HQ449186

GTTGGGGTTTTCCAGCACGGGAAAGTGGAAATCATTGCAAACGACCAGGGTAACAGGACCACGCCCAGTTATGTGGCATTCACAGACTCCGAGCGTCTCATTGGAGATGCCGCAAAGAACCAAGTGGCCATGAACCCCGAGAACACAGTCTTCGACGCCAAACGTTTGATCGGACGCAAGTTTGACGACTCAGCCGTTCAGTCAGACAAAAAGCATTGGCCATTCGATGTGGTCAGTGAGGGTGGCAAACCAAAGATCTCTGTTGATTACAAGGGGGAGAAGAAATCATTTTACCCAGAAGAGATCTCCTCCATGGTATTGGTTAAGATGAAGGAAACAGCCGAGGCTTACATCGGCAAGACTGTTCTAAATGCCGTCGTAACAGTGCCTGCCTACTTCAACGATTCTCAGCGCCAAGCAACCAAAGATGCCGGAACTATCTCTGGCTTGAATGTATTACGTATCATCAATGAACCAACG

>Paralvinella_cB1i_EF580993

GTTGGTGTATTCCAGCATGGCAAGGTGGAAATCGTAGCTAATGATCAGGGAAACCGAACGACGCCAAGTTATGTGGCGTTTACGGATTCCGAACGTATTATTGGAGATGGAGCCAAGAACCAAGTGGCCATGAACCCCAGTAACACGGTGTTCGATGCGAAACGTCTCATTGGTCGTAGGCTCGATGATTCGTCCGTCCAATCAGATATGAAGTTCTGGCCCTTCAAGGTCATCAGTGAGAATGGCAAGCCAAAAATCCAAGTGGAGTACAAGGGGGAGTTGAAGACCTTCTATCCCGAAGAAATTTCCTCCATGGTACTTTTGAAAATGAAAGAAACTGCTGAAGCCTATCTTGGAAAGAACGTACAGAGCGCTGTGATCACAGTACCAGCCTATTTCAATGACAGCCAGCGTCAGGCCACCAAAGATGCTGGTACCATCTCTGGTATGAATGTGCTGCGTATCATCAATGAGCCCACA

>Helobdella_cB1_XM_009023904

GTTGGAATTTTTCAGAATGGGAAAGTGGAAATAATTGCGAACGATCAAGGAAACAGAACCACACCAAGCTACGTCGCTTTCACAGACGATGAAAGATTGATAGGTGATCCGGCTAAAAATCAGGTTGCGATAAATCCAGGAAACACAGTTTTTGACGCAAAAAGGCTCATTGGTAGAAAGTACGATGATCCTTCCGTTCAGTCAGATATCAAATTGTGGCCGTTTAAAGTGATTAACGACAAGGGAAAGCCGAAAATTCTAGTGGAGTATAAAGGAGAGAAAAAATCTTTTTTCCCGGAAGAAATTTCGTCAATGGTTTTGATAAAAATGAAGGAGGTTGCTGAATCATTCATTGGAAAGCAAGTGAACGATGCAGTGATAACGGTGCCAGCGTACTTCAACGATTCGCAGCGACAAGCGACAAAAGACGCCGGCACGATAGCTGGATTGAACGTCCTTCGAATTATAAACGAACCAACT

>Helobdella_cB2_XM_009031197

GTGGGTATCTTTCAACATGGAAAGGTCGAGATCATCGCAAACGATCAAGGCAATAGGACGACTCCCAGTTACGTGGCTTTCACTGACTCAGAACGTCTCATTGGTGATGCTGCCAGAAATCAGGTAGCCATGAACCCCACCAATTCAGTTTTCGATGCCAAACGTTTGATTGGTAGAAAATTTGATGATCCCTCCGTCCAATCTGATATGAAGCACTGGCCATTCACGGTAATAAGCGATGGTGGAAAACCCAAGATACAAGTTGAATACAAAGGCGAAACTAAAACGTTTTATCCAGAGGAGATTTCATCTATGGTTTTGCTTAAAATGAAAGAAACTGCTGAGGCATATCTTGGTAAAACTGTCTCAGATGCTGTAGTAACAGTGCCTGCATATTTCAATGATTCCCAGCGTCAGGCTACTAAAGATGCAGGAACAATCTCTGGTTTAAATGTTTTAAGAATCATTAATGAACCCACT

>Lottia_cB1_XM_009048116

GTGGGTGTTTTTCAACATGGAAAAGTTGAAATTATCGCCAATGATCAGGGTAACAGAACCACCCCTAGTTATGTTGCTTTCACAGACACCGAACGATTGATTGGTGATGCTGCAAAAAATCAAGTTGCTATGAACCCAGAAAACACAGTATTTGATGCTAAGAGACTAATTGGTAGAAAGTTTGACGATCAACACGTACAAAATGACATCAAACATTGGCCATTTACTGTTGTAAATGATAACACCAAACCAAAAATTCAAGTACAATACAAAAATGAATTGAAAACATTTGCAGCAGAAGAAGTCTCATCAATGGTGTTGACTAAAATGAAGGAAACCGCTGAAGCTTATCTGGGAAAGAAAGTATCGGATGCTGTTGTCACAGTTCCTGCCTATTTCAACGATTCTCAAAGACAAGCCACTAAAGATGCTGGTACCATTGCTGGTTTAAATGTTTTGCGTATTATCAACGAGCCTACA

>Octopus_cB1_XM_014927588

GTAGGTGTCTTTCAACATGGCAAGGTTGAAATCATTGCCAATGACCAAGGAAACCGTACCACTCCCAGCTATGTGGCATTTACTGATACCGAGCGTCTCATCGGAGATGCTGCTAAGAACCAAGTCGCCATGAACCCAGAGAGCACCGTTTTTGATGCAAAACGTCTAATCGGTCGTCGATTTGATGACAAATCAGTCCAGAGTGACATGAAACACTGGCCGTTTACGGTGACGAATGAAAACACTAAACCAAAACTGCGGGTTATGTTCAAAGGAGAAGAGAAGACCTTCTTTCCTGAGGAAATCTCTTCTATGGTTTTAACCAAGATGAAGGAAACAGCTGAAGCCTATCTGGGCAGGACTGTAACAAACGTGGTGGTCACTGTCCCCGCCTACTTCAACGACTCCCAGAGACAGGCTACCAAAGATGCCGGTACTATTGCCGGTCTAAATGTCTTGAGAATCATTAACGAACCCACC

>Octopus_cB2_XM_014929288

GTAGGTGTTTTTCAACATGGTAAAGTCGAAATCATTGCCAACGATCAGGGTAATCGCACTACACCAAGCTATGTTGCTTTCACTGATACAGAACGTCTTATTGGCGATGCTGCCAAAAATCAAGTTGCTATGAATCCCGAAAACTCCATATTTGATGCTAAGAGATTAATAGGTAGAAGATTTACTGACGAGGTCGTGGTGGCGGATGCAGCTCTCTGGCCTTTCAAAGTTATCAATGATAATGGCAAGCCTAAAGTAAGAGTTAACTTTAAGGGAGAAGCAAAGACGTTTTCTCCAGAAGAAATTTCCTCCATGGTTCTTACCAAAATGAAAGAAACTGCTGAGGCATATTTAGGAAAGACTGTAACCAATGCTGTAGTTACTGTTCCTGCATACTTCAATGACTCTCAGAGACAGGCCACTAAAGATGCTGGTACCATTGCTGGTCTGAATGTTTTGCGTATAATTAATGAACCTACA

>Aplysia_cB1_XM_005097955

GTAGGCGTTTTCCAGCATGGCAAAGTTGAGATCATTGCCAACGATCAGGGAAACAGAACAACCCCAAGCTATGTAGCCTTCACAGACAATGAGCGTCTCATTGGAGATGCTGCCAAGAACCAGGTGGCCATGAATCCCGAAAACACAGTCTTTGATGCCAAGAGGCTCATTGGTCGCAAATGGGATGAACCATGTGTGGCATCTGACATGAAACATTGGCCATTCAATGTCATCAATGAAGGTGGAAAGCCAAAGATCAGTGTGGACTACAAGGGTGAATCCAAACTGTTCTTCCCTGAAGAAATCTCTTCCATGGTGCTAACAAAAATGAAGGAAACCGCAGAAGCCTATCTTGGCAAGACGGTCACAGATGCTGTGGTCACAGTGCCTGCCTACTTCAACGATTCTCAACGTCAAGCAACAAAAGACGCTGGAACCATCTCAGGCCTCAACGTTTTGAGAATCATCAATGAACCCACA

>Haliotis_cB1_FJ812176

GTAGGGGTATTTCAACACGGAAAGGTAGAAATCATTGCCAATGACCAAGGAAACAGAACAACACCCAGCTATGTTGCCTTCACCGACACAGAGCGTCTCATTGGTGACGCTGCAAAGAACCAGGTTGCCATGAACCCAGAGAACACTATCTTCGATGCCAAACGTCTGATTGGTAGAAGATTTGATGAGACAAATGTTCAATCAGACATGAAGCACTGGCCATTCAATGTGTTGAGTGATGGAGGCAAACCCAAGATCCAAGTAAATTACAAAGATGAACCAAAAACTTTCTACCCTGAAGAGATTTCCTCCATGGTGCTAACAAAGATGAAGGAAACTGCAGAACAGTATTTGGGAAAGACTATAACAGACGCTGTTGTAACAGTCCCAGCTTACTTCAACGACTCTCAGCGACAGGCCACTAAAGATGCAGGGACAATCTCTGGTCTCAATGTTTTACGTATCATCAATGAGCCTACT

**Supplementary Fig. S6.** Sequences used to calculate Ka/Ks values in Fig. S7. We included 77 cytosolic genes. Shorter regions were used compared to the analysis in Fig. 4 because no gaps were allowed for calculation of Ka/Ks values.

**A**

**B**

**Supplementary Fig. S7**. Bee swarm boxplots of Ka/Ks values for each clade (A) and p values in the multiple comparison (B). Only Ka/Ks values calculated from inter-cluster pairs were averaged. Statistical differences were calculated by Kruskal-Wallis test (chi-squared = 200.45, df = 5, p-value < 2.2e-16) followed by the non-parametric post-hoc tests (pairwise Wilcox test with P value adjustment by the Holm method).
